# Peripheral Transcriptomic Signatures Reveal Convergent Neuroinflammatory, Metabolic, and miRNA Dysregulation in Major Psychiatric Disorders

**DOI:** 10.3390/biology15090673

**Published:** 2026-04-24

**Authors:** Ron Jacob B. Avila, Jhyme Lou O. De La Cerna, Lemmuel L. Tayo

**Affiliations:** 1 School of Chemical, Biological, and Materials Engineering and Sciences, Mapúa University, Manila 1002, Philippines; rjbavila@mymail.mapua.edu.ph (R.J.B.A.); jlodelacerna@mymail.mapua.edu.ph (J.L.O.D.L.C.); 2 School of Graduate Studies, Mapúa University, Manila 1002, Philippines; 3 Department of Biology, School of Health Sciences, Mapúa University, Makati 1203, Philippines

**Keywords:** neuropsychiatric disorders, WGCNA, hub genes, neuroinflammation, microRNA, dendritic pruning

## Abstract

**Background/Objectives**: Although clinically distinct, bipolar disorder (BP), schizophrenia (SZ), major depressive disorder (MDD), and social anxiety disorder (SAD) share fundamental biology. We mapped these transdiagnostic systemic mechanisms. **Methods**: Weighted Gene Co-Expression Network Analysis (WGCNA) of peripheral blood RNA-Seq datasets evaluated module preservation, hub gene disruption, and microRNA (miRNA) networks. **Results**: Seven modules showed robust cross-disease preservation. Overall, 56 of 105 candidate hub genes exhibited altered expression, with 22 passing the false discovery rate (FDR) correction. Hubs like IL1B, TLR2, and MMP9 dominated networks linked to altered inflammatory signaling and structural remodeling. Downregulated ribosomal hubs characterized systemic metabolic stress. **Discussion**: These signatures capture extensive systemic dysregulation. Inflammation and metabolic shifts correlate strongly with pathways regulating chronic neuroinflammation, epigenetic control, and dendritic pruning. Computational models suggest these cascades evade miRNA controls, potentially compromising structural neural plasticity. **Conclusions**: This shared transcriptomic architecture challenges rigid diagnostic boundaries. Identifying systemic immune dysregulation and translational alterations as core pathogenic denominators provides a rationale for transdiagnostic therapies targeting upstream systemic networks to mitigate neural vulnerabilities.

## Simple Summary

Historically, several mental health conditions like schizophrenia, bipolar disorder, major depressive disorder, and social anxiety disorder have been treated as separate brain diseases. To explore their shared biology, underlying physical overlaps across these illnesses were computationally investigated by mapping broad gene activity patterns from publicly available blood sample datasets. Through the resulting computational analysis, a shared foundation of an overactive immune system and altered metabolism was observed. Furthermore, a dysregulation of the body’s natural ability to control inflammation was identified, by which protective brain structures were predicted to gradually degrade over time. Thus, it was demonstrated that these psychiatric disorders might be deeply interconnected whole-body illnesses rather than isolated neurological defects. This conclusion computationally supported a shift in how mental health care should be approached. Because long-term cognitive decline was frequently not prevented by standard medications that merely adjust local brain chemicals, a broader therapeutic direction was suggested. Hence, if the body’s immune and metabolic systems are targeted by future treatments, patients’ memory and cognitive skills can be better protected, offering a highly effective path forward for psychiatric care.

## 1. Introduction

Neuropsychiatric disorders (NPDs) sit at the complex intersection of neurology and psychiatry. These conditions manifest as a multidimensional overlap of cognitive, affective, and motor symptoms rooted in shared genetic predispositions [1]. The resulting global health burden is staggering. NPDs currently account for 15.6% of all years lived with disability. By 2030, they are projected to cost the global economy an estimated USD 6 trillion, exceeding the economic burden of cancer and chronic respiratory diseases [2]. Isolated localized defects cannot fully explain this pathogenesis. Instead, multiple interacting systemic mechanisms appear to drive the core pathology, including chronic low-grade neuroinflammation, circadian misalignment, and systemic neurotransmitter dysregulation [3,4,5,6]. These cascading alterations ultimately contribute to bioenergetic impairment. Synaptic plasticity degrades, and regional neuroanatomical volume drops in metabolically demanding areas like the prefrontal cortex and hippocampus. This is hypothesized to be driven by mitochondrial dysfunction, exacerbated oxidative stress, and hypothalamic–pituitary–adrenal (HPA) axis overactivity [7]. Genetic pleiotropy compounds this physical vulnerability. Significant neurotrophic deficits, particularly the loss of brain-derived neurotrophic factor (BDNF), putatively suppress neurogenesis and amplify a patient’s vulnerability to environmental stressors [8].

Diagnostically, SZ, BP, MDD, and SAD maintain distinct clinical classifications driven by well-documented differences in their primary symptomatology and classical localized neurobiology. SZ is characterized by psychotic episodes and cognitive decline, which are classically linked to striatal dopamine hyperactivity and cortical N-methyl-D-aspartate receptor (NMDAR) hypofunction [9]. BP presents with cyclical shifts between mania and depression, frequently associated with generalized glutamatergic hyperexcitability and unique monoamine fluctuations [10]. In contrast, Major Depressive Disorder is defined by persistent low mood and anhedonia, historically attributed to severe serotonergic and GABAergic deficits within the forebrain [11,12]. Furthermore, SAD is driven by context-specific fear responses that are heavily modulated by localized neuropeptide imbalances and amygdala hyperactivity [13].

Despite these distinct clinical boundaries and divergent primary neurotransmitter targets, these conditions share an underlying structural and molecular overlap. While their canonical pathways differ significantly, they correlate with parallel neuroanatomical changes. Progressive cortical thinning occurs across these conditions. Patients consistently exhibit compromised white matter integrity and reduced gray matter volume in the prefrontal cortex [9,10,11]. This physical restructuring manifests clinically as severe cognitive impairment, apathy, anhedonia, and affective instability [4,5,6,14,15,16]. Looking closer, the underlying molecular drivers are highly interconnected. The specific pro-inflammatory cytokines associated with synaptic loss in MDD, such as TNF-α and IL-6 [17], mirror the chronic neuroinflammation and mitochondrial dysfunction central to BP [15]. Complex polygenic risk architectures interact heavily with these inflammatory cascades. Susceptibility involves a broad landscape of canonical neurodevelopmental and synaptic risk loci. For instance, genetic vulnerability in SZ encompasses established synaptic coordinators like DTNBP1 and NRG1 [18]. Notably, this vulnerability extends to master regulators of synaptic vesicle exocytosis and calcium signaling, specifically DISC1 and CACNA1C. Recent evidence highlights that DISC1 directly modulates the transport and function of voltage-gated calcium channels like CACNA1C, forming a tightly coupled regulatory axis that mediates hippocampal neuron survival and presynaptic transmission [19,20,21]. This complex synaptic network is concurrently modulated by critical neurotransmitter-regulating enzymes like COMT. Moreover, this shared susceptibility simultaneously involves epigenetic alterations targeting HPA axis hyperactivity in SAD [22].

Comprehensive systemic transcriptome profiling offers the only viable way to uncover the shared molecular signatures across these diverse clinical phenotypes. This study aimed to identify conserved transcriptional patterns and co-expression modules. We utilized publicly available whole-blood RNA-sequencing datasets for SZ, MDD, BP, and SAD. We applied WGCNA to assemble gene co-expression networks. This isolated stable modules strongly preserved across these completely distinct psychiatric conditions. Establishing the biological significance of these networks required a highly integrated analytical framework. We first identified intramodular hub genes through protein–protein interaction (PPI) networks. Independent differential expression analyses then validated the actual transcriptional dysregulation of these specific hubs within their original disease cohorts. To highlight key post-transcriptional control axes, we computationally mapped putative miRNA regulators. We ultimately integrated the identified hub genes with genome-wide association study (GWAS) traits. This final step systematically classified disease associations to pinpoint the highly pleiotropic master regulators driving the transdiagnostic neuropsychiatric spectrum.

## 2. Materials and Methods

### 2.1. Dataset Acquisition

Transcriptomic datasets were retrieved from the publicly available Gene Expression Omnibus (https://www.ncbi.nlm.nih.gov/geo/ (accessed on 3 January 2026) (GEO) database [23]. To ensure cross-data comparability, all collections were screened using explicit inclusion parameters. Eligible datasets required (1) high-throughput RNA-sequencing platforms, (2) human peripheral blood-derived samples, and (3) a minimum sample size of *n* > 20 per diagnostic group. Table 1 details the specific cohorts encompassing BP, SZ, MDD, and SAD. Demographic breakdowns for each cohort including the number of disease group, controls, age and sex distributions are provided in Supplementary Table S1.

### 2.2. Preprocessing and Quality Control

Expression profiles across the BP, SZ, MDD, and SAD datasets underwent initial filtering to isolate genes present in at least 20% of the samples. Expression variance was stabilized and technical noise reduced by applying a variance stabilizing transformation (VST) via the DESeq2 package (version 1.51.6) [28]. Available age and sex metadata were integrated into the normalization design to mitigate confounding variables. A ~1 design matrix was utilized when this metadata was absent. Residual age and sex effects were subsequently regressed out utilizing the removeBatchEffect function from limma [29,30]. This step harmonizes expression signatures across the heterogeneous cohorts. Quality control was executed using the goodSamplesGenes function. Analysis was strictly restricted to genes shared across all four datasets for cross-data comparability. Following the standard WGCNA preprocessing framework [31], a median absolute deviation (MAD) filter was applied to isolate the 5000 most highly variable genes. This variance-based approach was explicitly chosen over differential expression filtering to prevent a priori statistical bias. Restricting the input eliminates uninformative background noise and spurious correlations, optimizing both the signal-to-noise ratio and the computational efficiency required to establish a reliable scale-free topology.

BP I (*n* = 226) and BP II (*n* = 14) participants from the GSE124326 dataset were merged into a single analytical group. While these subtypes present distinct clinical trajectories regarding mood elevation, they exist on established biological and genetic continuum. Previous genomic analyses confirm significant overlap in susceptibility loci between BP I and BP II, which is often associated with neurodevelopmental function [32]. Large-scale genetic studies also report a highly significant genetic correlation (r_g_ > 0.80) between the subtypes, indicating extensive shared genetic architecture rather than discrete molecular etiologies [33]. Furthermore, combining these samples ensures the robust construction of co-expression networks. Given the sample distribution, integrating the cohorts maximizes statistical power and maintains topological stability, allowing the predominant bipolar molecular signature to be accurately captured without introducing prohibitive biological heterogeneity.

### 2.3. WGCNA

Co-expression modules and conserved transcriptional patterns across the psychiatric conditions were identified using RStudio (version 4.4.1) and WGCNA Bioconductor packages (version 3.22). Network assembly and module detection occurred independently for the BP, SZ, MDD, and SAD datasets to facilitate direct cross-disease comparison. Prior to network construction, whole-blood composition was rigorously evaluated. Global leukocyte proportions were estimated via deconvolution using the CIBERSORT algorithm integrated with the ABIS matrix, confirming stable immune architecture across cohorts. To further ensure downstream modules represented true leukocyte biology rather than non-leukocyte whole-blood artifacts, a targeted sensitivity analysis was conducted. Utilizing the limma package (removeBatchEffect), empirical proxy scores for erythrocyte and platelet abundance (derived from lineage-exclusive MSigDB marker sets), alongside demographic covariates, were explicitly regressed out of the normalized expression matrices.

#### 2.3.1. Network Construction and Scale-Free Topology Estimation

Normalized expression matrices underwent scale-free topology compliance testing. The pickSoftThreshold function evaluated soft-thresholding powers within a signed network framework to calculate the scale-free topology model fit (R^2^). Optimal powers were selected to amplify robust gene-gene correlations and penalize false connections. This mathematical stringency promotes biologically accurate network architecture. The dataset exhibiting the highest scale-free topology fit served as the reference network for subsequent module preservation mapping [34,35].

#### 2.3.2. Module Detection and Topological Overlap-Based Clustering

Gene co-expression networks were generated by calculating Pearson correlation-derived adjacency matrices. These matrices were transformed into topological overlap matrices (TOMs). This transformation provides a highly robust connectivity estimate by evaluating both direct and shared neighbor interactions. Genes were hierarchically clustered via average linkage based on TOM dissimilarity. The dynamic tree cut algorithm identified distinct co-expression modules, utilizing a minimum module size of 30 genes and a deep split value of 2. Module eigengenes representing the first principal component of each module’s expression matrix were computed. Intramodular connectivity was quantified via module membership (kME), calculated as the Pearson correlation between individual genes and their respective eigengenes. This metric ranks genes by connectivity and pinpoints central hub genes driving the underlying biological networks.

#### 2.3.3. Module Preservation Evaluation

The modulePreservation function assessed the cross-disease stability of the identified networks [36]. Preservation values were initially calculated utilizing 100 permutations and re-evaluated at 1000 permutations to guarantee statistical stability. The composite Z summary statistic quantified module stability by integrating network density and connectivity metrics. Modules were stratified by preservation strength (Table 2). Only strongly preserved networks (Z summary > 10) advanced to downstream functional analysis. These high-scoring networks represent core, conserved biological pathways shared across the distinct psychiatric disorders rather than dataset-specific transcriptomic artifacts.

### 2.4. Function Annotation, Pathway Analysis, and Network Characterization of Modules

Central hub genes were identified through an integrative network-based methodology. Genes within highly preserved networks were prioritized by their module membership (kME) scores. The STRING database (https://string-db.org (accessed on 15 January 2026)) was queried to construct protein-protein interaction (PPI) networks, applying a strict medium confidence cutoff of 0.4 [37]. Network topology was subsequently evaluated using Cytoscape (v3.10.4). The CytoHubba plugin calculated degree centrality to quantify the number of functional connections per node, isolating the most highly interconnected targets [38]. The top 15 nodes per conserved biological pathway were officially designated as the core regulatory hub genes. This strict threshold serves as an established network heuristic to capture the most critical biological regulators while actively filtering out lower-degree peripheral noise, an approach consistent with recent systemic transcriptomic evaluations of neurodegenerative and cancer networks [30,39].

### 2.5. Targeted Pathway Enrichment for Microbiota–Gut–Brain Axis Interactions

To assess the functional alignment of these 105 hub genes with the microbiota–gut–brain axis, targeted Gene Ontology (GO) and KEGG pathway enrichment analyses were performed using the clusterProfiler R package (FDR < 0.05). Enriched terms were specifically filtered to isolate host immune responses to microbial triggers, such as lipopolysaccharide (LPS) exposure and Toll-like/NOD-like receptor cascades.

### 2.6. Differential Expression Analysis of Module Derived Hub Genes

Independent differential expression analyses were executed for the BP, SZ, MDD, and SAD cohorts using DESeq2. Cohort-specific design formulas were applied to control for underlying technical and biological confounding variables. To establish a standardized and dimensionally equivalent transcriptomic profile across all four disorders for downstream cross-referencing, the top 3000 genes from each dataset were extracted based on their nominal statistical ranking (lowest *p*-values). The 105 total hub genes derived from the network mapping were then directly cross-referenced against these expanded cohort-specific expression profiles. A tiered evidence framework (Table 3) subsequently quantified the clinical disease relevance of these targets.

This framework guarantees candidate hub genes maintain topological centrality while exhibiting consistent transcriptional dysregulation within their original biological context [44]. Analyzing datasets independently actively preserves data integrity and mitigates cross-cohort batch effects. A threshold of |log2FC| ≥ 0.5 was applied to capture moderate but highly significant expression shifts [24,35,40,41]. While oncological studies often employ a ≥1.0 cutoff, polygenic psychiatric conditions rarely exhibit massive fold changes due to systemic regulatory buffers and intense cellular heterogeneity. Therefore, 0.5 serves as an empirically validated threshold in psychiatric transcriptomics to exclude background noise while successfully capturing subtle, actionable network-level alterations.

### 2.7. miRNA-Module Regulatory Determination and Enrichment Analysiss

Putative miRNA regulators of the conserved biological networks were computationally inferred utilizing the multiMiR package. This tool integrates experimentally validated and predictive miRNA-target databases. Highly connected intramodular hub genes (kME ≥ 0.6) served as prioritized candidate targets. Enrichment for specific miRNAs was statistically evaluated using Fisher’s exact test with false discovery rate correction against a background of all preserved network genes. Candidate regulators were subsequently ranked using a composite metric weighting statistical significance, experimental validation, computational support, and direct hub targeting. This generated ranked miRNA lists and predictive regulatory networks highlighting core post-transcriptional mechanisms.

### 2.8. Integration of Hub Genes with GWAS-Identified Traits

Central hub genes derived from the network analyses were systematically annotated for established disease and trait associations using the Open Targets Platform (https://platform.opentargets.org/ (accessed on 19 January 2026)) [45]. The genes were mapped directly back to their respective functional networks, and their associated clinical phenotypes were classified into neuropsychiatric, neurodegenerative, neuroinflammatory, and systemic categories. Subsequent analyses quantified the total disease associations per gene to pinpoint highly pleiotropic master regulators. This systematic integration provides a structured overview of shared genetic architecture. Also, it explicitly highlighted central genes driving primary BP, SZ, MDD, and SAD phenotypes.

## 3. Results

### 3.1. WGCNA

#### 3.1.1. Data Pre-Processing and Estimation of Scale-Free Network

**Figure 1:**
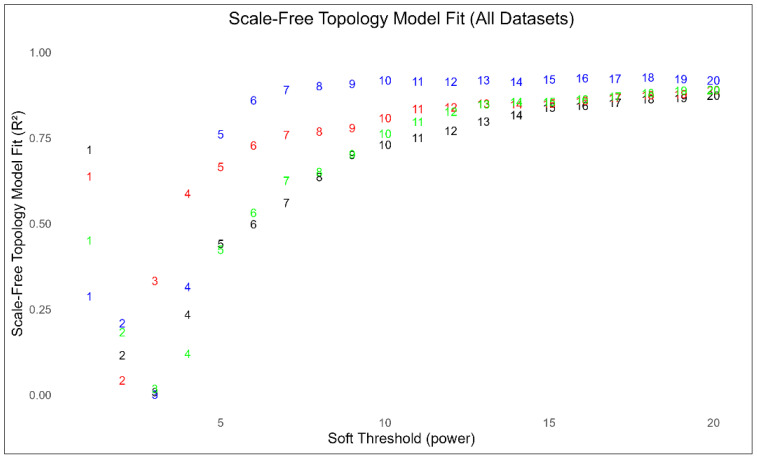
Psychiatric disorder scale-free topology model: SAD (blue), MDD (green), SZ (red), and BP (black).

Initial quality control and normalization prepared the four datasets for comparative analysis (Figure 1). VST corrected the mean-variance relationship inherent in count-based expression data. Missing values were imputed while strictly preserving original gene and sample counts. Extracting intersecting genes across all cohorts isolated 15,098 shared features. The MAD filter ranked these features by expression variability, retaining only the top 5000 genes. This parameter strips away technical noise. It isolates the robust biological signals required to construct informative molecular networks for BP, SZ, SAD, and MDD. In addition dispersion estimates can bee seen in Figure S1 as part of the data quality assurance.

**Figure 2:**
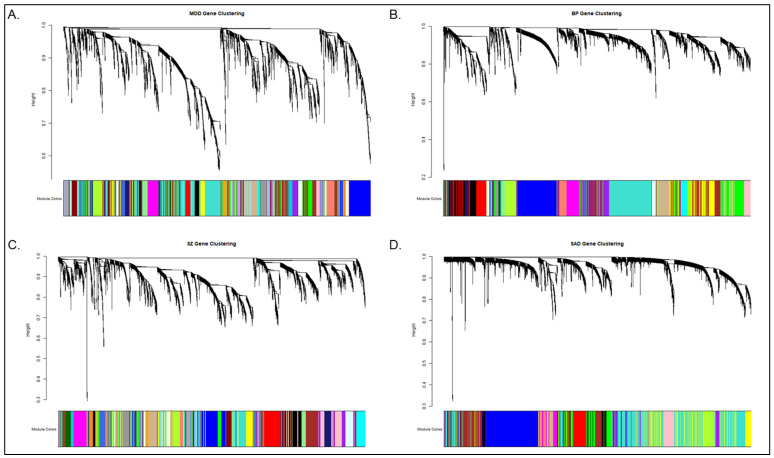
Gene clustering graph of the psychiatric disorders: (**A**) MDD, (**B**) BP, (**C**) SZ, and (**D**) SAD. The lower bar represents the module colors associated with their respective dataset.

Scale-free topology tests evaluated these filtered matrices to ensure suitability for downstream analysis (Figure 2). Biological gene networks characteristically display non-random, hierarchical structures. All four clinical cohorts met this topological requirement at moderate soft-thresholding powers.

The BP and SZ networks exhibited highly comparable architectures. BP reached a scale-free fit index (SFT.R^2^) of 0.84 at a power of 16. SZ achieved an SFT.R^2^ of 0.85 at a power of 15, displaying slightly higher overall connectivity. The SAD cohort demonstrated a distinctly dense wiring pattern. It achieved a high model fit (SFT.R^2^ = 0.86) at a substantially lower power of 6. The MDD network maintained scale-free properties (SFT.R^2^ > 0.85) across powers 13 to 15. This reflects a calculated balance between network connectivity and mathematical model fit. 

#### 3.1.2. Gene Co-Expression Module Detection Using TOM Similarity

The SAD dataset exhibited the strongest association with scale-free topology. It was selected as the reference framework for TOM-based co-expression networks. Hierarchical clustering of TOM dissimilarity exposed distinct modular architectures across the four clinical cohorts. Dynamic tree cutting optimized these primary module assignments. This adaptive algorithm facilitates module detection without requiring a rigid dendrogram cut height.

The approach yielded dataset-specific dendrogram height ranges, as observed on the *y*-axis of the clustering trees in Figure 2, that reflect the underlying differences in network density: MDD (0.5–1.0), BP (0.2–1.0), SZ (0.3–1.0), and SAD (0.3–1.0). These ranges were determined directly by the distinct hierarchical clustering structure of the topological overlap matrix (TOM) dissimilarity within each cohort, allowing the algorithm to dynamically detect modules based on the shape of the branches rather than a pre-set static threshold. Smaller cut values generate detailed divisions into smaller modules. Larger values produce fewer, highly integrated clusters. The SAD and BP networks formed large, densely integrated modules indicative of highly connected co-expression structures. MDD and SZ produced dispersed module distributions. Rather than strictly reflecting biological transcriptional diversity, this topological fragmentation indicates a more modular statistical architecture characterized by distinct, independent clusters of co-expressed genes within these specific datasets.

### 3.2. Module Preservation Analysis

**Figure 3:**
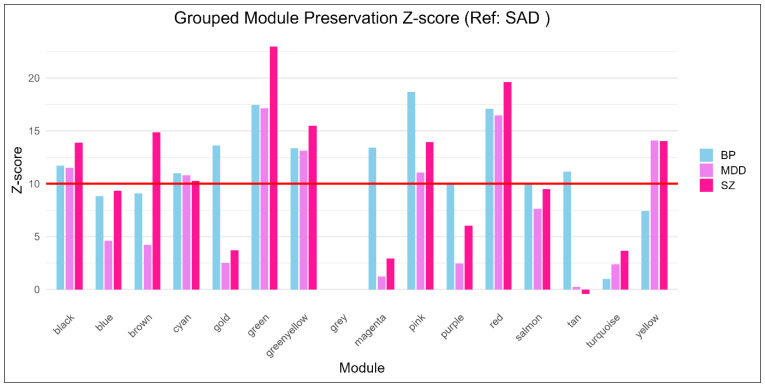
Preservation module bar plots based on Z-score. SAD served as the reference.

SAD served as the reference dataset to evaluate network stability across MDD, SZ, and BP (Figure 3, Table 4). The Z-summary statistic quantified this structural preservation. Scores exceeding 10 indicated robust network stability. Seven modules achieved strong preservation across at least two clinical cohorts: green (307 genes), green-yellow (150 genes), red (210 genes), pink (186 genes), cyan (41 genes), yellow (570 genes), and black (193 genes). These clusters exhibited topological overlap and deeply conserved co-expression patterns. This could be seen in Dataset S1.

The red module registered exceptionally high Z-scores across MDD, SZ, and BP despite its moderate size. This highlights a rigid, highly stable transcriptional program governing core cellular processes shared universally across these conditions. The green and green-yellow modules displayed identical high-level preservation. This structural integrity points to conserved molecular pathways underlying common neurobiological features in both mood and psychotic phenotypes. The yellow module encompasses the largest conserved gene set. It maintained rigid preservation in BP and MDD but broke down significantly in SZ. This topological fragmentation suggests a divergence in the correlation structure of these genes within the SZ cohort, rather than a definitive biological spatial rearrangement. The smaller cyan and black modules exhibited highly selective preservation. Their relevance may differ between disorders.

### 3.3. Validation of the Leukocyte Origin of Transdiagnostic Networks

Initial in silico deconvolution via the CIBERSORT algorithm (ABIS matrix) confirmed a stable baseline immune architecture, with the mean proportions of 17 distinct leukocyte subpopulations remaining highly consistent between cases and controls across all cohorts (Figure S24). Subsequent evaluation of non-leukocyte transcripts indicated a uniform distribution of background noise across the MDD, SAD, and SZ datasets for both erythrocyte (MDD: *p* = 0.68; SAD: *p* = 0.57; SZ: *p* = 0.17) and platelet populations (MDD: *p* = 0.32; SAD: *p* = 0.53; SZ: *p* = 0.35). The BP dataset, however, presented a distinct statistical imbalance in erythrocyte abundance (*p* = 0.0013), while platelet estimates remained stable (*p* = 0.51) (Figure S25).

Subsequent comparisons between the primary and covariate-adjusted networks demonstrated extensive topological conservation, confirming that this BP-specific compositional variance did not drive the underlying module architecture. The central biological drivers remained intact; cross-referencing the top 15 master regulatory hub genes revealed that the primary green, red, and yellow modules mapped seamlessly to the adjusted green, magenta, and yellow modules. These conserved networks retained their primary neuro-immune regulators, including MMP9, TLR2, and IL1B. Protein–protein interaction networks detailing the top 15 hubs for the seven topologically stable modules (black, green, magenta, pink, purple, tan, and yellow) are available in Figures S26–S32. The overlap between the original and filtered networks validates the authentic leukocyte origin of these transdiagnostic signatures.

### 3.4. DEA, Functional Annotation, and PPI Network of Preserved Gene Co-Expression Modules

The modules that were carried forward in the analysis were chosen for their robust preservation across the datasets. This is indicative of a conserved co-expression network. Additionally, these modules form the basis for finding hub genes and then using them as targets, since the genes of interest are encoded in a conserved and reproducible transcriptional network rather than being based on a data-driven signal [46].

Differential expression analysis was performed on 105 candidate hub genes (15 genes per module), and functional annotation was carried out using the GeneCards database and literature review to assess the disease relevance of the prominent regulatory drivers [47]. Recognizing that disease-associated transcriptome alterations may be subtle, a tiered differential expression approach (Table 3) was used, as moderate and consistent fold changes may also indicate biologically significant alterations. By integrating module preservation information and the tiered differential expression approach, we were able to rank genes according to their role in conserved co-expression modules and the disruption of transcriptional modules across diseases [48]. Using this tiered system, 56 of the 105 candidate hub genes were identified as exhibiting altered expression. Importantly, 22 of these genes successfully passed strict FDR correction (Tier 1), while the remaining targets met nominal p-value or fold-change criteria (Tiers 2 and 3) to capture more subtle but consistent biological trends. Among the diseases examined, the greatest number of disrupted genes was found in BP with 22 genes, and the lowest number was found in SAD with 17 genes.

Consequently, the top 15 hub genes for each preserved module were obtained using CytoHubba degree-based network analysis, and these genes represent the key regulators in their respective co-expression modules. These hub genes represent the basic constituents of their modules because of their high degree of connectivity, which places them at the center of protein–protein interaction (PPI) networks. The connectivity of nodes in these networks is used as a proxy for module interactions, and nodes with high connectivity are indicative of regulatory roles. These hub genes together form a list of potential key regulators of immune function, transcription, and stress response that have been implicated in NPDs [27,29,49,50,51,52,53,54].

#### 3.4.1. Module Results Analysis

WGCNA identified seven distinct modules associated with the transcriptomic landscape of the studied cohorts. The functional themes, enriched biological processes, and key signaling pathways for each module are summarized in Table 5. Network topology analysis was subsequently utilized to identify highly interconnected hub genes within each module, revealing how these regulatory units drive specific pathophysiological mechanisms. Moreover, the full differential expression profiles and functional annotation can be seen in Table S2 and Tables S3–S9, respectively.

#### 3.4.2. Neuroimmune and Inflammatory Response Networks

**Figure 4:**
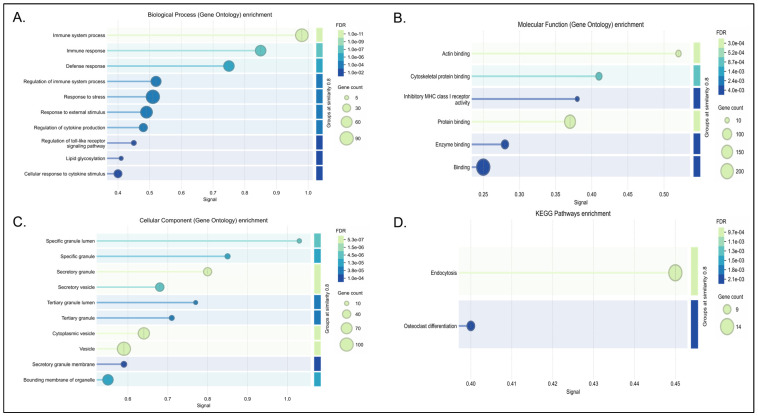
Green module functional annotation and GO enrichment analysis. (**A**) GO BP, (**B**) GO MF, (**C**) GO CC and (**D**) KEGG.

Five of the identified modules (Green, Red, Yellow, Cyan, and Green-yellow) converge on distinct but overlapping facets of neuroinflammation, innate immune signaling, and microglial activation. The green and red modules primarily regulate immune cell migration, phagosome-related debris clearance, and inflammatory cascades which was also mentioned in Figure 4 and Figures S2, S4 and S5 [55,58]. Within the green module, hub genes such as MMP9 are strongly associated with structural brain changes; MMP9 putatively remodels the extracellular matrix to facilitate immune cell migration and influence synaptic plasticity through the BDNF/TrkB pathway [58,91]. This is computationally coordinated with innate immune signaling adapters, including TYROBP and TNFRSF1A, which are linked to microglial activation and apoptosis [56,57,58,92,93,94]. Similarly, the red module amplifies these processes via a coordinated unit centered on TLR2 and SPI1 [60,95]. TLR2 correlates with MyD88-dependent inflammatory cascades that are typically exacerbated under chronic stress [96,97].

**Figure 5:**
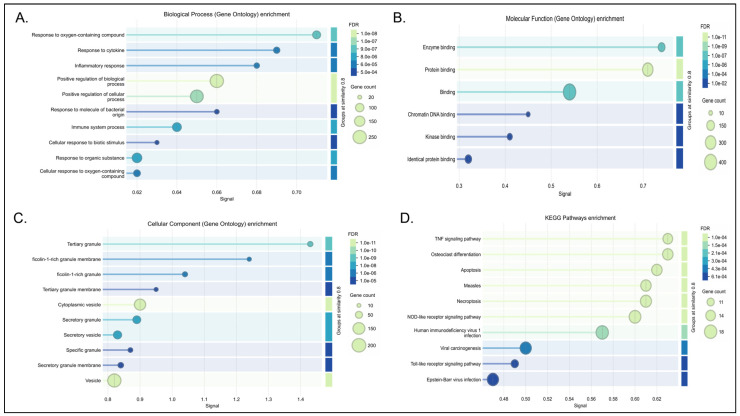
Yellow module functional annotation and GO enrichment analysis. (**A**) GO BP, (**B**) GO MF, (**C**) GO CC and (**D**) KEGG.

The yellow module uniquely highlights brain responses to oxidative stress and inflammation via the TNF signaling pathway as seen in Figure 5 and Figure S8. At the network level, this module suggests a delicate balance between AKT1, a master regulator of cell survival [82,83,98], and IL1B, a pro-inflammatory cytokine known to promote apoptosis and inhibit long-term potentiation [80]. The structural correlates of this signaling are mediated by RHOA and the AP-1 transcription factor complex (JUN/FOS), computationally linking acute stress signals to long-term changes in neural plasticity and actin cytoskeleton reorganization [79,80,81].

**Figure 6:**
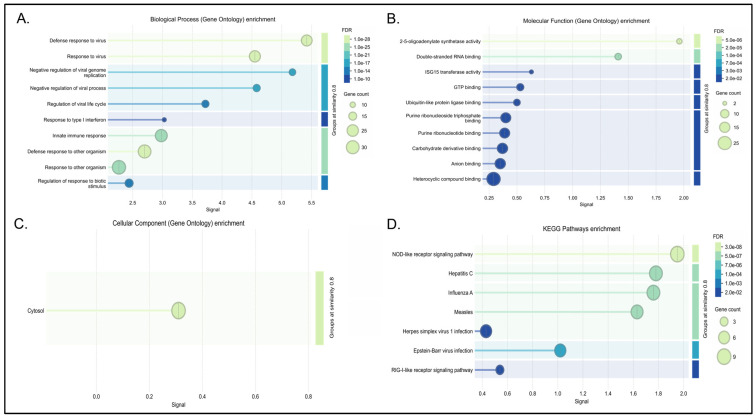
Cyan module functional annotation and GO enrichment analysis. (**A**) GO BP, (**B**) GO MF, (**C**) GO CC and (**D**) KEGG.

Conversely, the cyan module functions as a core interferon-response module (IRM) as seen in Figure 6 and Figure S9. It utilizes antiviral hubs like OAS1 and RSAD2 to putatively amplify TLR4/NF-κB microglial signaling [86,87,88,89,90]. Bridging these diverse immune reactions to neuronal health is the green-yellow module (Figures S3 and S7), which focuses entirely on chemokine signaling. Specifically, hubs such as HCK are associated with inflammasome activation and mitochondrial dysfunction [69,70], while PXN links these immune signals to the alternative splicing of synaptic factors [71].

#### 3.4.3. Dysregulation of Cellular Metabolism and Protein Translation

**Figure 7:**
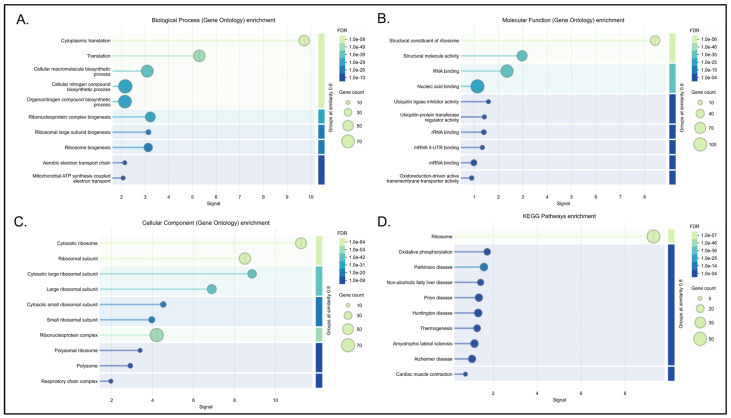
Pink module functional annotation and GO enrichment analysis. (**A**) GO BP, (**B**) GO MF, (**C**) GO CC and (**D**) KEGG.

The pink module highlights a strong mechanistic link between translational machinery and cellular energy metabolism, demonstrating robust enrichment for protein translation and the aerobic electron transport chain (Figure 7 and Figure S6). At the network level, the topology suggests how ribosomal function putatively influences neurodevelopment and neuroinflammation. The translational elongation factor EEF1B2 emerges as a computationally critical node for neurodevelopment [67]. Furthermore, core ribosomal hubs (including RPS27A, RPL9, and RPS3) are computationally linked to the modulation of immune responses; collectively, these hubs are associated with limiting proinflammatory infiltration via the NF-κB axis, suppressing TNF-α production, and providing neuroprotection against reactive oxygen species and lipid peroxidation [64,65,66].

#### 3.4.4. Disruption of Neurodevelopment and Synaptic Plasticity

**Figure 8:**
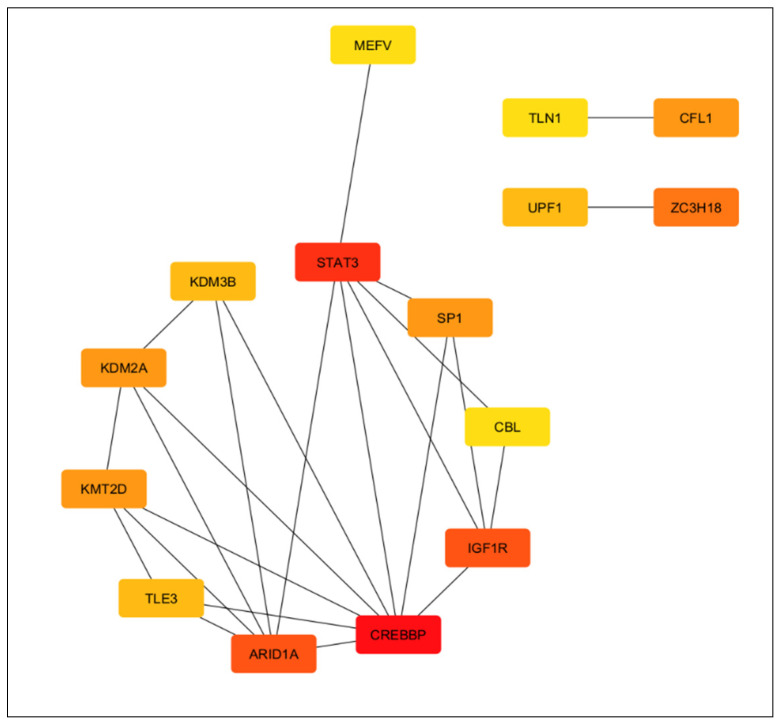
Top 15 hub genes in the Black module.

Unlike the other networks, the black module acts as an integrative, pleiotropic network that lacks significant enrichment for specific Gene Ontology pathways but computationally converges on neurodevelopment and synaptic plasticity. Network topology analysis identifies highly interconnected hub genes that putatively govern brain plasticity, neurogenesis, and complex glial-neuronal interactions (Figure 8). Within this modeled network, ARID1A is associated with the regulation of microglial polarization; its disruption correlates with an inflammatory surge of cytokines such as IL-6 [76]. his modeled surge computationally links to the activation of STAT3, suggesting a theoretical pathway by which the inflammatory response translates into altered neurodevelopmental trajectories [73,77,99,100]. Concurrently, this inflammatory state is associated with a suppression of the IGF1R-mediated neuroprotective environment [75,78]. These combined network disruptions are theoretically linked to deficits in synaptic plasticity and memory which are processes centrally associated with the hub gene CREBBP [74].

#### 3.4.5. Disorder-Specific Transcriptomic Signatures and Module Dysregulation

Differential expression analysis of the module hub genes identified distinct transcriptomic signatures that differentiate the pathophysiological mechanisms of SZ, BP, MDD, and SAD. While neuroinflammation is a shared characteristic, the specific nature of the immune dysregulation varies across cohorts. Hub genes within the myeloid and chemokine signaling modules demonstrate broad cross-disease upregulation. For instance, the complement receptor C5AR1 is upregulated across BP, MDD, and SAD. However, the interferon-response network (cyan module) exhibits a highly divergent signature: core antiviral hubs (including OAS1 and GBP5) are distinctly downregulated in BP, contrasting with the upregulation of RSAD2 observed in SAD. Similarly, the yellow module highlights disorder-specific stress responses, characterized by the upregulation of pro-inflammatory mediators like IL1B in BP, compared to the downregulation of the critical survival kinase AKT1 in SAD.

Moreover, cross-disease divergence occurred within the metabolic and translational network (pink module). A widespread, highly significant downregulation of core translational hub genes (such as EEF1B2 and multiple ribosomal subunits) suggests impaired protein synthesis is a major transcriptomic feature of SZ. In contrast, BP exhibits a putatively compensatory or hyperactive translational signature, marked by the significant upregulation of a distinct subset of ribosomal genes.

Finally, the neurodevelopmental and plasticity network (black module) reveals vulnerabilities tied to specific psychiatric phenotypes. Hub genes governing chromatin remodeling and synaptic integrity, such as ARID1A and CBL, are uniquely downregulated in SAD. Meanwhile, KMT2D is upregulated in SZ, and the actin-modulating factor CFL1 exhibits opposing regulation across disorders (upregulated in MDD, downregulated in SAD). This underscores how disruptions in shared neuroplasticity networks computationally manifest as distinct clinical pathologies.

#### 3.4.6. Enrichment for Host-Microbiome Interaction Pathways

To evaluate the potential systemic influence of the microbiota–gut–brain axis, targeted pathway enrichment of the 105 identified hub genes was performed. Gene Ontology analysis revealed a significant overrepresentation of gene sets associated with host immune responses to microbial triggers, most notably “response to lipopolysaccharide” and “cellular response to molecule of bacterial origin” (Figure S22). Concurrently, KEGG analysis highlighted significant overlap with the “NOD-like receptor signaling pathway,” “Toll-like receptor signaling pathway,” and “Inflammatory bowel disease” (Figure S23). These in-silico transcriptomic alignments suggest that the identified peripheral immune networks putatively overlap with pathways known to be responsive to circulating microbial components and gut-barrier permeability, offering theoretical support for gut-derived systemic signaling.

### 3.5. miRNA–Gene Regulatory Interaction Networks

#### 3.5.1. Interaction Landscape: Validated vs. Predicted Network

While our multiMiR analysis maps extensive post-transcriptional networks, a substantial proportion of these miRNA-gene interactions are computationally inferred rather than empirically validated. Consequently, these networks largely represent theoretical regulatory models. The proportion of experimentally validated versus computationally predicted miRNA-gene interactions varied considerably across the network modules.

**Figure 9:**
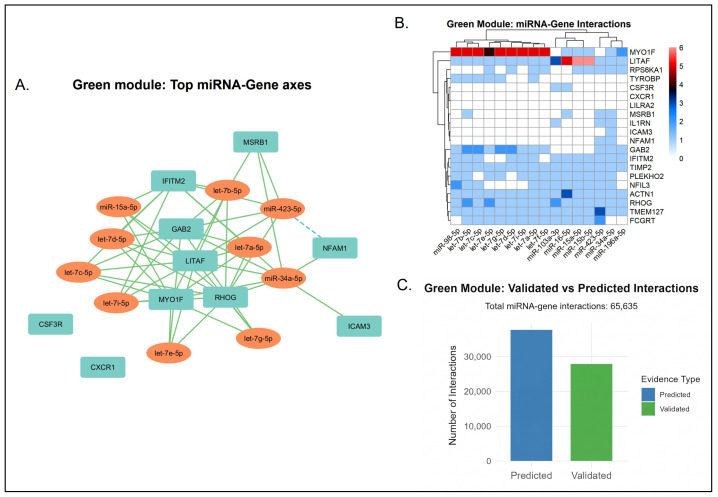
miRNA–gene interaction profile of the green module: (**A**) Network visualization depicting the top miRNA–gene regulatory axes. To explicitly separate evidence types, experimentally validated interactions are denoted by solid green edges, while computationally predicted interactions are denoted by dashed blue edges. (**B**) Heatmap of the top-ranked miRNAs and their interactions with green module genes, illustrating relative connectivity patterns across the module. (**C**) Distribution of validated versus predicted interactions, depciting the relative contributions of experimentally supported and computationally inferred edges. Note: Interactions denoted as predicted (blue edges) are derived computationally from the multiMiR database and currently lack direct experimental validation; these axes should be interpreted with appropriate caution as inferred post-transcriptional models.

Networks mapping to highly conserved processes exhibited a more balanced distribution of experimental evidence. For instance, the green module identified 65,635 total interactions, of which 27,929 (42.6%) were experimentally validated (Figure 9). Similarly, the pink module showed a 44.8% validation rate across its 47,230 interactions. Specific transcripts within these networks demonstrated complete experimental backing; all identified targets for miR-100-5p in the pink module and miR-21-3p in the cyan module were 100% validated (Figures S13 and S14).

Conversely, the interaction landscapes of the yellow, red, green-yellow, and black modules were dominated by computational predictions (Figures S10–S12). The yellow module represented the largest regulatory network, comprising 161,637 total interactions. However, it is crucial to note that 98,549 of these interactions (61%) are computationally predicted, representing hypotheses rather than confirmed mechanisms. This network was heavily influenced by the miR-548 family, where prediction rates exceeded 90%.

A similar pattern was observed in the red module, which contained 59,288 total interactions. Within this module, 36,088 interactions (60.9%) were predicted, with specific miRNAs like miR-4307 relying almost entirely (98.57%) on computational inference. These data indicate a substantial volume of uncharacterized regulatory space within these specific functional networks, which must be interpreted with caution as putative models pending future empirical validation. The specifics of the data can be seen in Datasets S2 and S3.

#### 3.5.2. Core miRNA Signatures with Established Regulatory Targets

Our analysis identified several miRNAs that serve as central nodes across multiple functional modules. To ensure mechanistic accuracy, we distinguish between those with empirical evidence and those representing computational hypotheses. Focusing on experimentally validated interactions, miR-34a-5p, miR-15a-5p, miR-17-5p, and members of the let-7 family represent the most prominent cross-module regulators. Their functional scopes extend beyond isolated biological domains. Based on established literature, miR-34a-5p acts as a major p53 effector, influencing neuronal growth and modulating dopamine catabolism via COMT regulation [101,102]. The let-7 family, particularly let-7b-5p, is known to regulate cell differentiation while exerting both pro- and anti-inflammatory effects [103]. miR-15a-5p has been shown to modulate BDNF expression to control neuronal proliferation [104], and miR-17-5p operates as a neuroprotectant by modifying inflammatory apoptosis [105]. Table 6 outlines the validated and predicted miRNA-gene regulatory axes for each module. The integration of these post-transcriptional interactions proposes a theoretical framework for the regulatory mechanisms underlying the main pathophysiological themes. Moreover, the miRNA-gene interaction per module can be seen in Figures S15–S19.

#### 3.5.3. Evidence-Supported Regulatory Networks in Neuroimmune Responses

Microglial activation and innate immunity are regulated post-transcriptionally within the green, red, yellow, cyan, and green-yellow networks. Based on empirically validated interactions, extracellular matrix remodeling and immune cell migration in the green and red modules are controlled by targeting MMP9, TLR2, and SPI1. miR-16-5p, which influences serotonergic and stress response pathways [106,107], interacts with MMP9 alongside miR-34a-5p to regulate synaptic stability and blood–brain barrier permeability. The red module integrates miR-136-5p to suppress inflammatory responses by targeting key mediators of toll-like receptor and cytokine signaling pathways [108].

Within the yellow module, transcripts such as miR-103a-3p target HMGB1 to attenuate oxidative stress and neuroinflammation in microglial regulation Concurrently, miR-424-5p targets KIF23, linking cytoskeletal remodeling to cellular invasiveness [109]. These miRNAs interact with AKT1, IL1B, and the AP-1 complex (JUN/FOS) to coordinate activity-dependent transcription and cellular survival pathways. The cyan and green-yellow networks illustrate direct regulation of interferon-driven antiviral defenses and microglial quiescence. The brain-enriched transcript miR-124-3p targets the kinase HCK to maintain inflammatory balance [110] while miR-27a-5p modulates NF-κB-mediated signaling by targeting the interferon effectors OAS1 and IFI35 [111].

#### 3.5.4. Validated Interaction Nodes in Cellular Metabolism and Protein Translation

Post-transcriptional control of protein synthesis and energy homeostasis is localized within the pink module. The core validated regulatory miRNAs collaborate with transcripts possessing highly specific metabolic functions. miR-20b-5p targets mechanisms related to calcium homeostasis and downregulates APP [112]. miR-26a-5p attenuates microglial activation by targeting the TREM1-TLR4/MyD88/NF-κB inflammatory axis [113].

These miRNAs exert precise control over components of the translational machinery, targeting the elongation factor EEF1B2 and ribosomal proteins RPL27, RPS27A, RPS20, and RPS3. Notably, RPS27A and RPS20 are targeted simultaneously by miR-34a-5p, miR-20b-5p, miR-26a-5p, and miR-17-5p, placing them at the center of this regulatory network. RPS3 also functions in DNA repair, indicating that this miRNA network integrates ribosomal assembly with direct neuroprotective responses [66].

#### 3.5.5. Established Post-Transcriptional Networks Associated with Neurodevelopment

**Figure 10:**
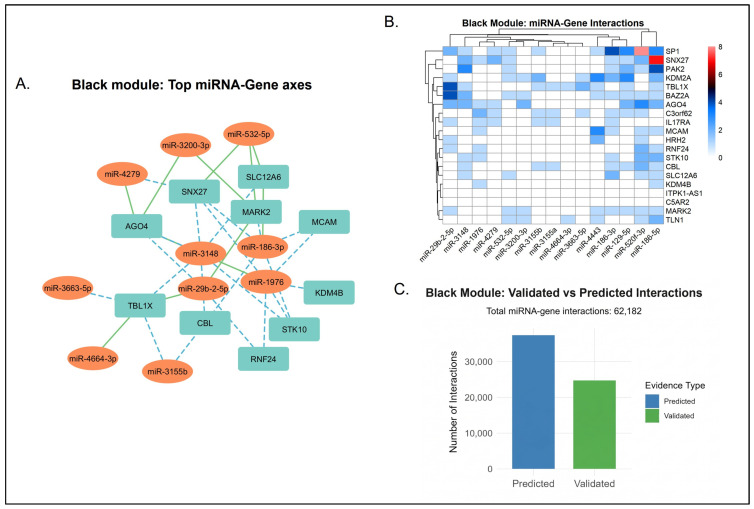
miRNA–gene interaction profile of the black module: (**A**) Network visualization depicting the top miRNA–gene regulatory axes. To explicitly separate evidence types, experimentally validated interactions are denoted by solid green edges, while computationally predicted interactions are denoted by dashed blue edges. (**B**) Heatmap of the top-ranked miRNAs and their interactions with black module genes, illustrating relative connectivity patterns across the module. (**C**) Distribution of validated versus predicted interactions, depicting the relative contributions of experimentally supported and computationally inferred edges. Note: Interactions denoted as predicted (blue edges) are derived computationally from the multiMiR database and currently lack direct experimental validation; these axes should be interpreted with appropriate caution as inferred post-transcriptional models.

The black module characterizes the epigenetic regulation required for long-term brain plasticity as seen in Figure 10. The experimentally validated master miRNAs within this network interact with interconnected hubs that convert inflammatory signals into developmental alterations. let-7b-5p targets IGF1R, providing regulatory control over trophic support, developmental timing, and astrocyte maturation [78,114].

Furthermore, miR-34a-5p and miR-17-5p interact with CREBBP and STAT3. This specific interaction modifies epigenetic plasticity and mediates the downstream effects of cytokine signaling on neuronal survival [115,116]. let-7b-5p and miR-15a-5p target ARID1A, directly linking miRNA expression to the proliferation of neural stem and progenitor cells [117]. Alterations in this miRNA-gene circuit compromise the integration of epigenetic modulation and neuroimmune function, providing a mechanistic basis for cognitive symptoms in psychiatric conditions.

#### 3.5.6. Hypothetical Axes from Computationally Predicted Interactions

Computationally predicted interactions suggest a theoretical secondary layer of post-transcriptional control that may help balance immune responses with neuronal survival. Across the immune-driven networks (green, yellow, green-yellow, red, and cyan), these inferred models propose links between microglial inflammasome activity to ongoing structural maintenance. For example, the green module suggests miR-2113 targets MMP9 potentially modulate extracellular matrix turnover [118,119]. This structural regulation is predicted to overlap with signaling events where miR-548c-3p and miR-340-5p map to inflammatory mediators and cytoskeletal hubs like IL1B, HCK, RHOA, and PXN [120,121]. Phagocytic and interferon responses are hypothesized to undergo similar post-transcriptional modulation. Transcripts such as miR-186-5p and miR-522-3p are computationally predicted to target microglial receptors and antiviral effectors, specifically TLR2, SPI1, OAS1, and GBP1 [122,123].

Beyond strict immune function, the pink module localizes putative metabolic and translational checkpoints. Recurring regulators like miR-590-3p, miR-7-5p, and miR-214-5p target extensive ribosomal machinery, which includes EEF1B2 and RPS3, potentially serving to support protein synthesis during inflammatory stress [124,125,126]. A separate epigenetic architecture theoretically emerges within the black module. Putatively modulated by miR-548c-3p, miR-590-3p, miR-524-5p, and miR-520d-5p, this network converges on central developmental hubs. By targeting STAT3, ARID1A, CREBBP, and IGF1R, these miRNAs computationally connect chromatin remodeling directly to growth-factor cascades [121,125,127,128]. This entire predicted regulatory network is hypothesized to act as a molecular buffer against cytokine-driven damage, though these axes require future experimental validation to confirm their biological efficacy.

### 3.6. In Silico Validation of Hub Genes with GWAS Traits

**Figure 11:**
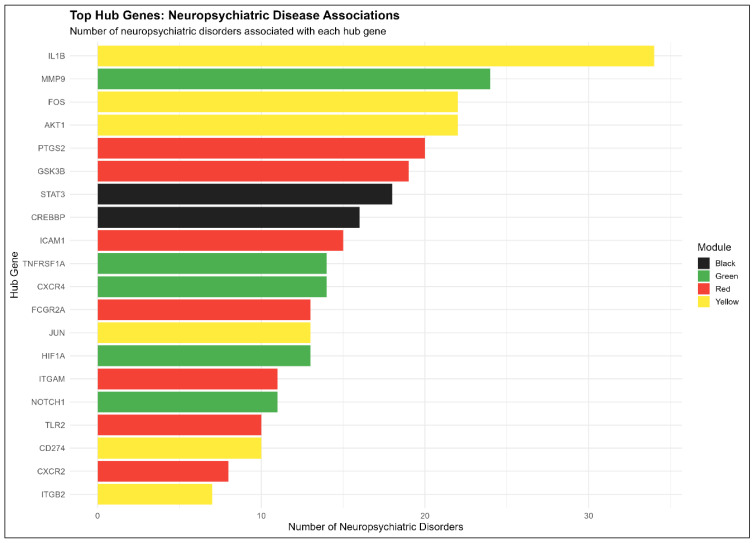
Top 20 neuropsychiatric associations among hub genes.

The analysis of 104 hub genes using OpenTargets identified 69,885 disease-related associations across 7663 unique disease conditions. The full detail can be seen in Dataset S4. Most of these genes, especially the top 20, showed strong pleiotropy, with a strong enrichment in primary NPDs; for instance, SZ and MDD were associated with 69 and 67 hub genes, respectively, reflecting shared genetic architecture. Notably, genes such as IL1B, MMP9, AKT1, and PTGS2 exhibited the highest total number of disease associations, linking to over 2000 unique conditions (Figure 9). While this reflects their broad roles in systemic inflammation, extracellular matrix remodeling, and intracellular signaling, these high association counts are inherently amplified by historical research intensity, as these are canonically well-studied targets. Therefore, these OpenTargets metrics highlight their general biological plausibility and transdiagnostic importance, rather than serving as direct causal evidence that these specific genes autonomously drive the transcriptomic networks observed in our cohorts. At the module level, the yellow module contributed most to these associations as IL1B and AKT1 part of it that integrate immune-inflammatory responses with neuronal survival as seen in Figure 11. Alternatively, the red and green modules represent a more specialized process. They are involved with prostaglandin synthesis and synaptic plasticity. These observations support that network-defined modules represent effective frameworks to encapsulate complex multi-systemic molecular profiles that define both broad neuropsychiatric phenotypes and specific diseases.

## 4. Discussion

Psychiatric research has historically interpreted the clinical symptoms of SZ, BP, MDD, and SAD as evidence of discrete and localized brain diseases [129,130]. However, recent shifts in systems biology suggest that the pathology driving these disorders may possess substantial transdiagnostic overlap [131,132]. Our transcriptomic analysis of peripheral blood networks aligns with this perspective, revealing a shared architecture of systemic biological dysregulation connecting all four conditions. This overlap is characterized by signatures of innate immune activation, metabolic stress, and structural remodeling. We identified conserved gene networks anchoring inflammatory effectors like IL1B and TLR2 alongside tissue remodelers like MMP9. Detecting these specific signatures in systemic circulation is notable because it offers a potential mechanistic link between chronic peripheral inflammation and central neurodevelopmental vulnerability. Our computational data suggest that the diverse behavioral symptoms observed clinically may diverge from a set of shared, upstream immune and metabolic disruptions.

This transdiagnostic framework is supported by its biological concordance with network analyses performed in the original, single-disorder cohorts. For example, the WGCNA conducted on the BP cohort identified distinct modules driven by Toll-like receptor innate immune signaling and ribosomal protein translation [24]. Similarly, the SZ investigation highlighted networks enriched for neutrophil degranulation and translational machinery [25]. Our pipeline independently clustered comparable biological axes across the combined datasets, specifically through our Red/Green (innate immunity) and Pink (metabolism/translation) modules. Furthermore, while the MDD and SAD studies primarily utilized discrete biomarker panels and standard differential expression rather than scale-free co-expression networking [26,27]. Our application of WGCNA organized their underlying inflammatory and stress-response signatures into these same measurable topological networks. While prior studies appropriately interpreted these disruptions within the context of their specific cohorts, our cross-disorder analysis suggests that these immune and translational modules represent a broader systemic signature spanning the psychiatric spectrum.

Building on this transcriptomic overlap, this study integrates these gene networks with miRNA regulatory data to further explore the shared biology underlying SZ, BP, MDD, and SAD. By identifying miRNAs that converge on the same immune, metabolic, and structural pathways disrupted in our gene-level networks, our work provides a potential mechanistic layer explaining how these systemic shifts might be coordinated. This regulatory alignment provides additional evidence for transdiagnostic biology: similar miRNA families appear to regulate systemic immune activation, metabolic stress responses, and structural dysregulation across disorders historically viewed as distinct. Ultimately, linking these peripheral gene networks with their miRNA controllers offers a more integrated model of psychiatric disease pathology, which may gradually inform future diagnostic frameworks and highlight new transdiagnostic therapeutic targets.

### 4.1. Transdiagnostic Immune Signaling and the Loss of Neuroprotective Integrity

The shared pathology across SZ, BP, MDD, and SAD appears to begin with an inflammatory shift that overwhelms the brain’s innate repair mechanisms. Network analysis explicitly maps this systemic immune hyperactivation to the red, yellow, and green modules. Within these networks, hub genes like TLR2 and IL1B act as central biological alarms. Pathway enrichment indicates they putatively shift microglia away from protective roles and toward a neurotoxic, activated state. This persistent peripheral activation predicts a severe degradation of brain architecture, mirroring the regional volume loss and synaptic pruning classically observed in the neuroimaging of severe SZ and BP [8,133,134]. The molecular correlates of this structural remodeling are linked to hubs like MMP9 and RHOA. While MMP9 normally facilitates careful tissue remodeling, its systemic upregulation points to a destructive mechanism that compromises the blood–brain barrier and degrades perineuronal nets [55,58,91]. Simultaneously, RHOA activation within the yellow module suggests an imbalance that aligns with the rapid shedding of dendritic spines, offering a molecular correlation for the memory decay and cognitive rigidity frequently observed in these patients [79,135]. Furthermore, chronic innate immune responses captured within the Cyan module implicate ongoing damage to local microvasculature and oligodendrocyte myelination. This suggests a direct link between peripheral interferon signaling and the white matter tract abnormalities detected in mood disorders [87,89,136].

This systemic inflammatory profile computationally aligns with disruptions in the brain’s ability to maintain neurogenesis, synaptic plasticity, and neuroimmune homeostasis. We isolated this specific functional failure within the Black module. Under healthy conditions, Black module hubs like the chromatin remodeler ARID1A and the growth factor receptor IGF1R act as a biological buffer to facilitate synaptic growth and protect neural pathways [75,114,117]. The transcriptomic data suggest the current inflammatory state compromises this protective barrier. Elevated circulating cytokines are likely linked to the over-activation of STAT3, which in turn suppresses the IGF1R-mediated neuroprotective environment. Clinically, this specific STAT3 over-activation is an established marker for the severe social withdrawal and depressive phenotypes characteristic of MDD and [73,77]. Ultimately, neuronal survival in these disorders hinge on a delicate molecular tug-of-war captured within the yellow module. This network houses both IL1B, which putatively drives apoptosis and blocks long-term potentiation [85], and AKT1, a master survival kinase whose reduced expression is an established genetic risk factor for SZ and BP [82,98]. When the transcriptomic balance shifts toward inflammatory signaling over protective pathways, it theoretically correlates with a reduced capacity to form stable synaptic connections.

### 4.2. Post-Transcriptional Dysregulation and Translational Alterations

The brain’s vulnerability to prolonged immune attack theoretically stems from alterations in post-transcriptional regulation. Computational network models suggest that miRNAs normally act as molecular governors. They keep hub gene activity within safe, homeostatic bounds. The consistent cross-disease upregulation of highly pleiotropic inflammatory hubs points strongly to a putative, broad-spectrum miRNA regulatory dysregulation. When these self-limiting biological brakes are impaired, acute systemic stressors may escalate into persistent dysregulation. For example, the computationally inferred loss of key miRNAs like miR-26a-5p and miR-34a-5p theoretically removes critical suppression over major inflammatory axes [113]. This post-transcriptional disinhibition permits master regulators like IL1B and AKT1 to operate with reduced constraint, potentially sustaining the maladaptive systemic inflammation described previously [82,83,84,98,137,138].

These inflammatory and oxidative signatures strongly correlate with pathways indicative of an energetic dysfunction in the central nervous system. Network analysis explicitly maps this systemic metabolic stress to the pink module. Pathway enrichment reveals this specific network governs protein translation, biosynthesis, and mitochondrial energy homeostasis. Under normal conditions, pink module ribosomal hubs like RPS27A, RPL9, and RPS3 actively protect the brain. They limit neuroinflammation and mitigate cellular damage during high stress [64,65,66]. Faced with metabolic exhaustion, cells theoretically execute a protective triage. They downregulate highly energy-intensive processes like protein synthesis to conserve their rapidly depleting energy reserves. The widespread suppression of elongation factors like EEF1B2 and structural ribosomal subunits leaves the central nervous system highly vulnerable and computationally lacks its intrinsic resilience [67]. This specific translational dysregulation acts as a primary molecular discriminator. SZ uniquely exhibits a systemic impairment of these biosynthetic pathways. This alteration in protein synthesis is mechanistically coupled to mitochondrial energy deficits [68]. This peripheral blood signature closely aligns with known central nervous system pathology. Single-nucleus RNA sequencing studies of postmortem SZ brains identify parallel, severe dysregulations in protein synthesis pathways within deep-layer excitatory neurons [134]. Similar glial energy disturbances linked to mitochondrial dysfunction are clinically observed in SAD [139].

Recognizing this metabolic and regulatory dysregulation exposes a limitation in conventional psychiatric care. Current pharmacotherapies primarily modulate downstream neurotransmission to manage acute symptoms [140,141,142,143,144]. These interventions often struggle to prevent progressive structural brain alterations and cognitive deterioration. Conventional treatments simply cannot repair a compromised systemic architecture. Systemic energetic dysfunction provides a clear biological explanation for the inability of standard receptor antagonists to correct persistent cognitive deficits in SZ [145]. Addressing psychiatric cognitive decline requires stabilizing this underlying metabolic machinery rather than exclusively adjusting synaptic neurotransmitters. Computationally predicted miRNA networks present upstream targets theoretically capable of modifying the disease course [143,144]. Modulating these missing biological brakes offers a strategy to bridge the gap between systemic immune activation and progressive neuronal damage.

### 4.3. Integration with GWAS

Matching our blood-derived expression networks to established GWAS traits serves primarily as a complementary validation layer for these predictive models. Peripheral biomarker research constantly struggles to separate actual pathology from background systemic noise. The pleiotropy of our identified central regulators cuts through that noise to suggest that dynamic RNA changes are deeply anchored in static genetic vulnerabilities. The transcriptomic signatures captured in the blood reflect systemic molecular changes tied directly to known genetic risk loci across broad psychiatric phenotypes. This overlap highlights how innate genetic risks potentially converge into interacting disease pathways. Hubs with high GWAS overlap scores like IL1B and AKT1 act as functional pivots balancing stress-induced neuroinflammation against metabolic resilience. It is important to note that these high association counts are naturally amplified by historical research intensity. Since canonical inflammatory targets are heavily investigated across numerous medical conditions, their database metrics reflect a combination of true biological pleiotropy and established literature bias. The translational dysregulation defined by ribosomal hubs overlaps with specific SZ risk traits, putatively supporting its role as a potential clinical discriminator. Moving beyond basic survival signaling, inflammatory effectors like MMP9 and PTGS2 exhibit lower direct genetic overlap but theoretically facilitate a coordinated cascade of neural damage in response to those primary genetic risks.

Epigenetic regulators like STAT3 and ARID1A show moderate genetic overlap and serve a completely different purpose. Their underlying genetic architecture exists to maintain neurodevelopmental integrity rather than mount acute immune responses. When these developmental pathways break down, the structural plasticity of the brain is severely compromised. Even nominal transcriptional shifts like the slight ARID1A downregulation captured in the SAD cohort can hypothetically suppress the brain’s neuroprotective environment. Pinpointing these master regulators highlights how peripheral molecular shifts collectively influence the biological pathways governing overall brain health.

### 4.4. The Microbiota–Gut–Brain Axis as a Potential Systemic Trigger

The systemic modules identified in this study feature an enrichment of innate immune and metabolic pathways, specifically Toll-like receptor signaling and matrix metalloproteinase activity. To computationally evaluate this potential link, targeted pathway enrichment of the core hub genes was performed. This in silico analysis revealed a significant overrepresentation of gene sets associated with host immune responses to microbial triggers alongside KEGG pathways including the “NOD-like receptor signaling pathway” and “Inflammatory bowel disease”. Consequently, this transcriptomic profile closely parallels the downstream inflammatory cascades known to be modulated by the microbiota–gut–brain axis. These alignments suggest that gut-derived systemic interactions may contribute to, rather than exclusively drive, the broader pathophysiological landscape of these disorders.

Healthy intestinal barriers normally keep microbial debris confined. In conditions such as SZ and MDD, this barrier can degrade, leading to intestinal hyper-permeability [146,147]. The consequences of this systemic endotoxemia align conceptually with our targeted enrichment results. Escaping gut-derived endotoxins (such as LPS) are hypothesized to bind directly to peripheral Toll-like receptors, theoretically explaining the systemic immune surge and specific IL1B upregulation observed in the BP cohort. This gut-induced response putatively triggers lipid-mediated inflammatory cascades across the peripheral immune system [148,149]. Escaping gut-derived endotoxins are hypothesized to bind peripheral Toll-like receptors, offering a theoretical framework for the systemic immune surge and specific IL1B upregulation observed in the BP cohort. This response putatively influences lipid-mediated inflammatory cascades across the peripheral immune system [150]. Beyond contributing to structural changes, microbial imbalance can also affect basic cellular metabolism. The translational decline we observed in the SZ group may reflect reduced energy supply linked to gut dysfunction. During pronounced dysbiosis, host tryptophan can shift away from serotonin production toward the kynurenine pathway, generating metabolites that increase oxidative stress in neural tissue [151]. This metabolic shunting exposes the nervous system to neurotoxic metabolites, further contributing to intracellular oxidative stress [152]. Concurrently, depleted beneficial bacteria produce fewer short-chain fatty acids. This reduction deprives cortical neurons of essential mitochondrial fuel and endogenous histone deacetylase inhibitors [153]. A reduction in these microbial regulators aligns theoretically with a loss of epigenetic control, mirrored by the inferred downregulation of ARID1A within the SAD cohort. Recent experimental models support this link, demonstrating that transplanting the gut microbiota of patients with SAD into healthy subjects induces severe social fear behaviors [154].

Ultimately, relying exclusively on conventional treatments that target downstream neural receptors may overlook this systemic pathology. Taken together, these findings raise the possibility that disruptions along the microbiota–gut–brain axis contribute to the systemic immune and metabolic abnormalities observed across psychiatric disorders. Although the present blood transcriptomic data do not directly measure the microbiome, our identified networks align with emerging work linking gut-derived signals to neural, inflammatory, and metabolic pathways. Future multi-omics studies integrating metagenomic profiles with transcriptomic and neurobiological measures will be essential to explicitly determine the extent to which gut–brain interactions shape psychiatric pathology.

### 4.5. Transdiagnostic Clinical Relevance and Therapeutics

The multi-systemic dysregulation mapped in these networks highlights a potential limitation in conventional psychiatric care. Current pharmacotherapies primarily target downstream neurotransmission to manage acute symptoms [140,141,142,143,144]. While effective for symptom management, these interventions often struggle to prevent progressive cognitive deterioration [145]. Our computational models suggest this occurs because standard treatments do not fully address the underlying systemic alterations. The predicted peripheral upregulation of innate immune effectors and structural modulators, specifically IL1B and MMP9 from the Green and Yellow modules, reflects a dysregulated inflammatory state. This state putatively operates alongside or independently of monoaminergic pathways. Speculatively, this peripheral immune activation might be exacerbated by systemic triggers like a compromised intestinal barrier within the microbiota–gut–brain axis [146,147,148]. Without targeted immunomodulatory strategies, MMP9-driven degradation of perineuronal nets may theoretically continue to impair synaptic plasticity regardless of local dopamine concentrations [55]. Standard receptor antagonists also do not directly target the significant metabolic shifts captured within the pink module. This computationally inferred energetic dysfunction provides a plausible biological context for the difficulty of standard treatments to correct persistent cognitive deficits [145]. This limitation is particularly relevant in SZ, where ribosomal machinery appears substantially downregulated. This suppression could hypothetically be influenced by broader systemic vulnerabilities, such as tryptophan shunting linked to gut dysbiosis [134,151].

Addressing psychiatric cognitive decline may ultimately benefit from stabilizing this underlying immune and metabolic machinery rather than focusing solely on synaptic neurotransmitters. Computationally predicted epigenetic and post-transcriptional networks present primary upstream targets theoretically capable of modifying the disease course [143,144]. Intervening at the level of the Black module, specifically targeting chromatin remodelers like ARID1A, could hypothetically help restore the neuroprotective environment required to mitigate persistent memory deficits. Replenishing essential microbial metabolites like short-chain fatty acids might offer a complementary peripheral approach to support this epigenetic restoration [153]. Realizing this therapeutic potential depends heavily on the associated miRNA regulatory landscape. Modulating disrupted biological regulators like miR-15a-5p could putatively normalize BDNF expression to mitigate synaptic density loss [104]. Modulating miR-34a-5p provides an inferred upstream method to regulate COMT and correct dopamine catabolism [102,104]. This modulation may simultaneously restrain the inflammatory axes predicted to drive cellular metabolic stress. Shifting the therapeutic focus toward these integrated systemic networks offers a theoretical transdiagnostic strategy. It provides a computationally derived rationale for treating psychiatric vulnerability by bridging the theoretical gap between peripheral immune activation, metabolic shifts, and progressive neuronal damage.

### 4.6. Blood vs. Brain Transcriptomic Analysis

The inaccessibility of living brain tissue has historically limited the molecular study of psychiatric pathology, prompting the use of peripheral blood as a dynamic proxy. While the correlation between peripheral and central molecular changes remains complex, recent multi-omics investigations provide supportive evidence for this approach. For example, comparative epigenetic analyses demonstrate that while single-site DNA methylation often exhibits tissue specificity, composite systemic signatures related to chronic inflammation and biological aging show significant cross-tissue correlation between peripheral blood and postmortem brain samples [155]. Furthermore, genetic vulnerabilities known to drive CNS alterations, such as the 17q21.31 haplotype, have been shown to manifest directly as distinctly detectable regulatory and epigenetic footprints in peripheral blood [156].

Beyond epigenetic factors, large-scale predictive transcriptomic models indicate that a substantial proportion of genetically regulated gene expression, including shared eQTLs, is conserved between whole blood and various brain regions [157]. This shared architecture supports the perspective that conditions such as MDD, SZ, BP, and SAD present as multi-systemic disorders. Under states of chronic stress, metabolic dysregulation, and systemic inflammation, peripheral cytokines may interact with or cross a compromised blood–brain barrier. This interaction is hypothesized to influence microglial activation, allowing circulating immune profiles to reflect CNS neuroinflammation and capturing how systemic metabolic factors correlate with the expression of key neurodegenerative biomarkers in the blood [158,159].

Consequently, the molecular shifts captured in circulation putatively reflect a systemic environment associated with localized neuronal damage. Identifying hub genes like MMP9 and IL1B within these peripheral modules aligns with this systemic-central axis [55,84]. In postmortem tissues, these targets correlate with structural deterioration, which points toward a potential mechanistic link between circulating immune signals and the breakdown of perineuronal nets [55,58,91]. This overlap extends to metabolic pathways. The downregulated ribosomal hubs suggest a systemic impairment in protein translation, providing an accessible proxy for CNS dysfunction [63,64,65,66,67,68]. Notably, this peripheral signature parallels the transcriptomic alterations found in deep-layer excitatory neurons of SZ brains through single-nucleus RNA sequencing [134]. While postmortem sequencing provides superior cellular resolution for localized pathology, blood transcriptomics offers a dynamic window into the systemic factors potentially influencing those changes [30]. Broader mappings of the blood-brain transcriptomic and inflammatory axis further support this concept [160]. Hence, capturing pleiotropic master regulators like AKT1 and PTGS2 helps bridge the gap between systemic inflammation and neuronal survival.

### 4.7. Limitations and Recommendations

While blood profiling captures this broad systemic pathology, the method has clear limitations. Foremost, this study relies entirely on peripheral blood RNA-seq, and any conclusions referring to brain-specific processes such as neuroinflammation, synaptic plasticity, or dendritic pruning must be interpreted cautiously. Although peripheral-to-central inference is common in psychiatric transcriptomics, peripheral markers provide an indirect reflection of central nervous system mechanisms, and the molecular networks described here represent inferential models rather than direct observations of brain tissue. Analyzing bulk blood obscures the complex microenvironment of the brain itself. It cannot pinpoint the exact cellular changes happening within specific cortical circuits. To bridge this gap, future investigations must directly compare these peripheral co-expression networks with large-scale, region-specific brain transcriptomic datasets, such as those generated by the PsychENCODE consortium. However, it should be noted that the primary transdiagnostic master regulators identified in this peripheral analysis, such as MMP9, TLR2, and IL1B, have been extensively validated in existing postmortem brain tissue and animal model literature, providing independent biological plausibility to our computational findings. Integrating these accessible peripheral compartments with post-mortem central nervous system data will be essential to establish a more comprehensive and mechanistically coherent understanding of how systemic immune and metabolic dysregulation permeates the blood–brain barrier.

Furthermore, a fundamental limitation of this study is the lack of external experimental validation. The findings are derived entirely from in silico analyses of four publicly available transcriptomic datasets. While our stringent inclusion criteria ensured high data quality and minimized batch effects, it consequently restricted our analysis to a single representative RNA-seq dataset per disorder. Additionally, our approach to defining transdiagnostic conservation carries inherent methodological constraints. We utilized a strict Z-summary > 10 to identify highly preserved modules, but we classified networks as conserved if they met this threshold across at least two comparisons. Grouping these modules into a single transdiagnostic signature potentially oversimplifies the pathology, as it assumes universal conservation and obscures the reality that certain networks may only be shared between specific disease pairs rather than uniformly across all four conditions. Although evaluating module preservation across these distinct cohorts provides a degree of internal statistical robustness, the absence of an independent, identically structured RNA-sequencing validation cohort limits the definitive strength and generalizability of the conclusions. The proposed gene networks and miRNA regulatory axes lack direct experimental validation, such as quantitative PCR (qPCR), Western blotting, functional in vitro assays, or molecular testing in highly standardized animal models. For instance, because the miRNA networks mapped here rely heavily on computational predictions, their roles in neuroinflammation and structural remodeling remain largely theoretical. Data from the Yellow module show that 61% of interactions lack empirical validation, while the red module reaches a 98.57% prediction rate. Highly compelling candidates like the miR-548 family require formal validation in a laboratory using human stem cell models or standardized in vivo models before their biological efficacy can be confirmed. Beyond computational constraints, the presence of RNA does not inherently guarantee actual protein activity. Incorporating large-scale proteomic profiling and targeted functional assays would be valuable for further dissecting these networks, as protein-level dynamics could validate the transcriptomic disruptions we identified and reveal post-transcriptional or signaling-level failures that are not fully captured at the RNA level.

Our datasets also rely on a cross-sectional design, meaning they only capture a single snapshot of these patients in time. This makes it difficult to definitively separate the molecular origins of the disease from the confounding side effects of long-term medications, which fundamentally alter systemic immune profiles and cellular metabolism. A recognized limitation of utilizing legacy transcriptomic cohorts is this pharmacological variance, such as the prominent lithium induced expression signature documented in the BP cohort (GSE124326). While covariate regression was utilized for continuous variables like cell-type proxies, the publicly available pharmacological metadata for this cohort lacks critical pharmacokinetic resolution (e.g., dosage, duration of exposure, and polypharmacy), making targeted mathematical regression of a binary ‘lithium status’ statistically unreliable. Moreover, the transdiagnostic module preservation methodology utilized in this study inherently mitigates cohort isolated pharmacological artifacts. Because our primary neuroimmune networks (e.g., the green and red modules) demonstrated robust topological preservation (Z summary > 10) across the SZ, MDD, and SAD cohorts, which are populations lacking lithium exposure, we can infer that these conserved molecular architectures are driven by the shared psychiatric phenotypes rather than isolated medication effects. Nonetheless, unmeasured lifestyle factors like diet, smoking status, and body mass index still heavily skew peripheral inflammatory markers. Future psychiatric transcriptomics must track medication of naive patients longitudinally to completely isolate pathogenic drivers from background noise. Pairing these blood profiles with matched cerebrospinal fluid would also offer a much sharper map of the immune brain interface, an essential step in translating these broad computational networks into actionable diagnostic tools.

## 5. Conclusions

Mapping the shared transcriptomic architecture of SZ, BP, MDD, and SAD reinforces a crucial biological reality. These conditions extend beyond strictly localized brain pathologies to operate as deeply interconnected multi-systemic disorders. Network analysis of peripheral blood datasets exposes a highly conserved molecular framework. Altered innate immune signaling and computationally inferred metabolic stress define this underlying architecture. Rather than merely correlating with central mechanisms, these captured peripheral signatures theoretically mirror the systemic environment associated with the localized remodeling of central synaptic networks. Identifying these dynamic molecular shifts highlights the immense clinical utility of accessible systemic biomarkers. The existence of these shared inflammatory and metabolic networks challenges rigid diagnostic boundaries. It provides a computationally derived rationale for treating psychiatric vulnerability as a transdiagnostic spectrum. Recognizing that structural neuronal vulnerability may be closely linked to continuous systemic immune activation highlights a clear limitation in conventional pharmacotherapy. Medications designed exclusively to adjust downstream neurotransmitter receptors cannot fully address these broader systemic alterations. Future therapeutic development would benefit from pivoting toward systemic interventions. Actively targeting these shared immune and metabolic pathways offers a highly comprehensive treatment strategy. Adopting this broader approach is essential, as it represents a viable path to mitigate progressive cognitive deterioration and support foundational neuronal resilience.

## Figures and Tables

**Table 1:** RNA-seq data summary of psychiatric disorders used in the study.

Accession No.	GSE124326 [24]	GSE243841 [25]	GSE251778 [26]	GSE228702 [27]
Condition	BP	SZ	MDD	SAD
Type	Expression Profiling by High Throughput Sequencing			
Source	Peripheral blood-derived samples			
Total Sample Size	480	181	169	160

**Table 2:** Module preservation Z summary thresholds.

Z Summary Score	Interpretation
Z summary > 10	High preservation evidence
2 < Z summary ≤ 10	Moderate preservation evidence
Z summary ≤ 2	No preservation evidence

**Table 3:** Adapted tier system of DEGs.

Tier	Criteria	Reference
Tier 1—High Confidence	FDR/*p* ≤ 0.05	Prevalent parameter in psychiatric studies. Utilized by Garcia et al. [35] and Krebs et al. [24].
Tier 2—Moderate Confidence	Nominal *p* < 0.05	The basis is on log2FC. This method is seen in the paper of Gandal et al. [40] and Akula et al. [41].
Tier 3—Cross Disease	Nominal *p* < 0.05 in ≥2 diseases/datasets, regardless of FDR	Representation of the replication across datasets, leveraging consistency over statistical stringency. This approach was seen in the paper of Castro-Martinez et al. [42] and Gandal et al. [43].

**Table 4:** Preservation pattern across psychiatric disorders in different modules with SAD as the reference dataset. Color patterns refer to high (green), moderate (yellow) and no preservation (red).

	BP	SZ	MDD
Black			
Blue			
Brown			
Cyan			
Gold			
Green			
Green-yellow			
Magenta			
Pink			
Purple			
Red			
Salmon			
Tan			
Turquoise			
Yellow			

**Table 5:** Summary of the identified gene co-expression modules, top hub genes, and their associated biological functions, signaling pathways and key regulating miRNAs.

Module	Hub Genes	Biological Function	Key Signaling Pathways	Key Regulating miRNAs	Reference
Green	MMP9, FCER1G, TYROBP, TNFRSF1A	Chronic neuroinflammation, neuronal apoptosis, and structural/synaptic remodeling	TNF/NF-κB signaling axis	miR-34a-5p, let-7b-5p, miR-2113	[55,56,57,58]
Red	TLR2, FCGR3B, ITGAM, SPI1	Neuroinflammation, microglial activation	MyD88-dependent TLR signaling axis and lipid-mediated inflammatory pathways	miR-34a-5p, miR-26a-5p, miR-186-5p	[59,60,61,62]
Pink	EEF1B2, RPL27, RPS27A, RPS3, RPL9	Protein translation and biosynthesis, mitochondrial energy homeostasis, and neuroinflammatory regulation	mTOR signaling, Oxidative Phosphorylation, and PSMD12/NF-κB axis	miR-34a-5p, miR-590-3p, miR-7-5p	[63,64,65,66,67,68]
Green-Yellow	HCK, FGR, PXN, LCP2	Neuroinflammation, alternative splicing and synaptic plasticity	AP1 signaling, SIRT1/PGC-1α axis, and NMDA/BDNF-paxillin signaling	miR-34a-5p, miR-196a-5p, miR-548c-3p	[69,70,71,72]
Black	STAT3, CREBBP, IGF1R, ARID1A	Neurogenesis, synaptic plasticity, and neuroimmune homeostasis	PI3K/Akt, and JAK-STAT signaling	miR-34a-5p, let-7b-5p, miR-548c-3p	[73,74,75,76,77,78]
Yellow	JUN, AKT1, IL1B, RHOA, FOS	Stress-Induced Synaptic Plasticity, Structural Remodeling, and neuroinflammatory regulation	ROCK2/LIMK/cofilin pathway, PI3K-Akt/GSK3 and MAPK/JNK signaling cascade	miR-34a-5p, miR-15a-5p, miR-548 family	[79,80,81,82,83,84,85]
Cyan	GBP1, IFI35, OAS1, RSAD2	Innate immune response and neuroinflammatory regulation	JAK-STAT and DAMPs signaling	miR-27a-5p, miR-21-3p, miR-590-3p	[86,87,88,89,90]

**Table 6:** Summary of experimentally validated and miRNA-gene interactions and their associated biological functions across the identified co-expression modules.

Biological Theme	Associated Module	Target Hub Genes	Key Validated Regulatory miRNAs	Key Predicted Regulatory miRNAs
Neuroimmune and Inflammatory Response	Green	MMP9, FCER1G, TNFRSF1A	miR-34a-5p, let-7b-5p, miR-15a-5p, miR-423-5p, miR-16-5p	miR-520a-5p, miR-302b-5p, miR-2113
	Red	TLR2, FCGR3B, ITGAM, SPI1	miR-34a-5p, miR-26a-5p, let-7a-5p, miR-136-5p	miR-186-5p, miR-370-3p, miR-4789-3p
	Yellow	JUN, AKT1, IL1B, RHOA, FOS	miR-34a-5p, miR-15a-5p, miR-424-5p, miR-423-5p, miR-103a-3p	miR-548c-3p, miR-4282, miR-340-5p, miR-548x-3p
	Cyan	GBP1, IFI35, OAS1, RSAD2	miR-27a-5p, miR-21-3p, miR-34a-5p, let-7a-5p	miR-590-3p, miR-548c-3p, miR-522-3p, miR-449a
	Green-Yellow	FGR, PXN, HCK	miR-34a-5p, miR-196a-5p, miR-423-5p, miR-106b-5p, miR-124-3p	miR-548c-3p, miR-3163, miR-607, miR-340-5p
Metabolism and Protein Translation	Pink	EEF1B2, RPL27, RPS27A, RPS20, RPS3, RPL9	miR-34a-5p, miR-20b-5p, miR-26a-5p, miR-17-5p	miR-590-3p, miR-7-5p, miR-548aa, miR-214-5p
Neurodevelopment and Synaptic Plasticity	Black	STAT3, CREBBP, IGF1R, ARID1A	miR-34a-5p, let-7b-5p, miR-15a-5p, miR-17-5p	miR-548c-3p, miR-590-3p, miR-524-5p, miR-520d-5p

## Data Availability

The transcriptomic datasets analyzed in this study are publicly available from the Gene Expression Omnibus (GEO) database (https://www.ncbi.nlm.nih.gov/geo/ (accessed on 3 January 2026)). The datasets include SZ (GSE243841), BP (GSE124326), MDD (GSE251778), and SAD (GSE228702).

## Supplementary Materials

The following supporting information can be downloaded at: https://www.mdpi.com/article/10.3390/biology15090673/s1, Figure S1: Dispersion Estimates of RNA-Seq Data in NPDs; Figure S2: Red module Functional Annotation and GO Enrichment analysis; Figure S3: Green-yellow module Functional Annotation and GO Enrichment analysis; Figures S4–S9: Top 15 hub genes (green, red, pink, green-yellow, yellow, cyan); Figures S10–S14: miRNA–gene interaction profiles (green-yellow, red, pink, yellow, cyan); Figures S15–S21: Hub gene-miRNA interaction profile (green, red, pink green-yellow, black, yellow, cyan); Figure S22. Gene Ontology (GO) enrichment analysis of core regulatory hub genes for microbiota–gut–brain axis interactions; Figure S23. KEGG pathway enrichment analysis of core regulatory hub genes highlighting host-microbiome interactions; Figure S24. Mean Leukocyte Proportions Estimated via ABIS Deconvolution; Figure S25. Empirical Proxy Scores for Non-Leukocyte Contamination Across Four Cohorts. Figures S26–S32. Top 15 hub genes (green, magenta, pink, purple, black, tan, yellow) from the scrubbed dataset. Table S1: Summary of Dataset Demographic and Disease Classification; Table S2: Cross-Disease Differential Expression of Candidate Genes in Psychiatric Disorders; Tables S3–S9: Biological Function and Psychiatric Relevance of Module Hub Genes and Direction (green, red, pink, green-yellow, black, yellow, cyan) [161,162,163,164,165,166,167,168,169,170,171,172,173,174,175,176,177,178,179,180,181,182,183,184,185,186,187,188,189,190,191,192,193,194]; Dataset S1: Summary of Preserved Modules in Different Permutations; Dataset S2: miRNA-Hub genes interaction; Dataset S3. Ranked miRNA interactions; Dataset S4: Hub genes GWAS results.

## Abbreviations

The following abbreviations are used in this manuscript:

NPDs	Neuropsychiatric Disorders
WGCNA	Weighted Gene Co-Expression Network Analysis
GWAS	Genome-wide Association Studies
MDD	Major Depressive Disorder
BP	Bipolar Disorder
SZ	Schizophrenia
SAD	Social Anxiety Disorder

## References

[1] Taslim S., Shadmani S., Saleem A.R., Kumar A., Brahma F., Blank N., Bashir M.A., Ansari D., Kumari K., Tanveer M. (2024). Neuropsychiatric Disorders: Bridging the Gap Between Neurology and Psychiatry. Cureus.

[2] Liu W., Zhang Y., Chen J., Li X., Huang Y., Zhao F., Chen F., Qu P., Li Y. (2025). Global Burden and Trends of Major Mental Disorders in Individuals under 24 years of Age from 1990 to 2021, with Projections to 2050: Insights from the Global Burden of Disease Study 2021. Front. Public Health.

[3] Tian H., Hu Z., Xu J., Wang C. (2022). The Molecular Pathophysiology of Depression and the New Therapeutics. Medcomm.

[4] Lee J.G., Woo Y.S., Park S.W., Seog D.-H., Seo M.K., Bahk W.-M. (2022). Neuromolecular Etiology of Bipolar Disorder: Possible Therapeutic Targets of Mood Stabilizers. Clin. Psychopharmacol. Neurosci..

[5] Luvsannyam E., Jain M.S., Pormento M.K.L., Siddiqui H., Balagtas A.R.A., Emuze B.O., Poprawski T. (2022). Neurobiology of Schizophrenia: A Comprehensive Review. Cureus.

[6] Stein D.J. (2015). Social Anxiety Disorder and the Psychobiology of Self-Consciousness. Front. Hum. Neurosci..

[7] Nunes P.I.G., Benjamin S.R., Brito R.d.S., de Aguiar M.R., Neves L.B., de Bruin V.M.S. (2025). Mitochondria, Oxidative Stress, and Psychiatric Disorders: An Integrative Perspective on Brain Bioenergetics. Clin. Bioenerg..

[8] Cui L., Li S., Wang S., Wu X., Liu Y., Yu W., Wang Y., Tang Y., Xia M., Li B. (2024). Major Depressive Disorder: Hypothesis, Mechanism, Prevention and Treatment. Signal Transduct. Target. Ther..

[9] Karlsgodt K.H., Sun D., Cannon T.D. (2010). Structural and Functional Brain Abnormalities in Schizophrenia. Curr. Dir. Psychol. Sci..

[10] Zhu Z., Zhao Y., Wen K., Li Q., Pan N., Fu S., Li F., Radua J., Vieta E., Kemp G.J. (2022). Cortical Thickness Abnormalities in Patients with Bipolar Disorder: A Systematic Review and Meta-Analysis. J. Affect. Disord..

[11] Fries G.R., Saldana V.A., Finnstein J., Rein T. (2023). Molecular Pathways of Major Depressive Disorder Converge on the Synapse. Mol. Psychiatry.

[12] Rana T., Behl T., Sehgal A., Singh S., Sharma N., Abdeen A., Ibrahim S.F., Mani V., Iqbal M.S., Bhatia S. (2022). Exploring the Role of Neuropeptides in Depression and Anxiety. Prog. Neuropsychopharmacol. Biol. Psychiatry.

[13] Martin E.I., Ressler K.J., Binder E., Nemeroff C.B. (2009). The Neurobiology of Anxiety Disorders: Brain Imaging, Genetics, and Psychoneuroendocrinology. Psychiatr. Clin. N. Am..

[14] James S.L., Abate D., Abate K.H., Abay S.M., Abbafati C., Abbasi N., Abbastabar H., Abd-Allah F., Abdela J., Abdelalim A. (2018). Global, Regional, and National Incidence, Prevalence, and Years Lived with Disability for 354 Diseases and Injuries for 195 Countries and Territories, 1990–2017: A Systematic Analysis for the Global Burden of Disease Study 2017. Lancet.

[15] Kim Y., Santos R., Gage F.H., Marchetto M.C. (2017). Molecular Mechanisms of Bipolar Disorder: Progress Made and Future Challenges. Front. Cell. Neurosci..

[16] Ren L., Fan Y., Wu W., Qian Y., He M., Li X., Wang Y., Yang Y., Wen X., Zhang R. (2024). Anxiety Disorders: Treatments, Models, and Circuitry Mechanisms. Eur. J. Pharmacol..

[17] Zhou Q., Lv X., Zhou S., Liu Q., Tian H., Zhang K., Wei J., Wang G., Chen Q., Zhu G. (2021). Inflammatory Cytokines, Cognition, and Response to Antidepressant Treatment in Patients with Major Depressive Disorder. Psychiatry Res..

[18] Riley B., Kendler K.S. (2006). Molecular Genetic Studies of Schizophrenia. Eur. J. Hum. Genet..

[19] Rittenhouse A.R., Ortiz-Miranda S., Jurczyk A. (2021). Mutations in *DISC1* Alter *IP3R* and Voltage-Gated Ca^2^+ Channel Functioning, Implications for Major Mental Illness. Neuronal Signal..

[20] Lee A.S., De Jesús-Cortés H., Kabir Z.D., Knobbe W., Orr M., Burgdorf C., Huntington P., McDaniel L., Britt J.K., Hoffmann F. (2016). The Neuropsychiatric Disease-Associated Gene *Cacna1c* Mediates Survival of Young Hippocampal Neurons. eNeuro.

[21] Tang W., Thevathasan J.V., Lin Q., Lim K.B., Kuroda K., Kaibuchi K., Bilger M., Soong T.W., Fivaz M. (2016). Stimulation of Synaptic Vesicle Exocytosis by the Mental Disease Gene *DISC1* Is Mediated by N-Type Voltage-Gated Calcium Channels. Front. Synaptic Neurosci..

[22] Merkouris E., Brasinika A., Patsiavoura M., Siniosoglou C., Tsiptsios D., Triantafyllis A.S., Mueller C., Mpikou I., Samara M.T., Christodoulou N. (2025). Molecular Basis of Anxiety: A Comprehensive Review of 2014–2024 Clinical and Preclinical Studies. Int. J. Mol. Sci..

[23] Clough E., Barrett T. (2016). The Gene Expression Omnibus Database. Statistical Genomics.

[24] Krebs C.E., Ori A.P.S., Vreeker A., Wu T., Cantor R.M., Boks M.P.M., Kahn R.S., Olde Loohuis L.M., Ophoff R.A. (2019). Whole Blood Transcriptome Analysis in Bipolar Disorder Reveals Strong Lithium Effect. Psychol. Med..

[25] Pan B., Li X., Weng J., Xu X., Yu P., Zhao Y., Yu D., Zhang X., Tang X. (2025). Identifying Periphery Biomarkers of First-Episode Drug-Naïve Patients with Schizophrenia Using Machine-Learning-Based Strategies. Prog. Neuropsychopharmacol. Biol. Psychiatry.

[26] Mokhtari A., Ibrahim E.C., Gloaguen A., Barrot C.-C., Cohen D., Derouin M., Vachon H., Charbonnier G., Loriod B., Decraene C. (2025). Using Multiomic Integration to Improve Blood Biomarkers of Major Depressive Disorder: A Case-Control Study. EBioMedicine.

[27] Edelmann S., Wiegand A., Hentrich T., Pasche S., Schulze-Hentrich J.M., Munk M.H.J., Fallgatter A.J., Kreifelts B., Nieratschker V. (2023). Blood Transcriptome Analysis Suggests an Indirect Molecular Association of Early Life Adversities and Adult Social Anxiety Disorder by Immune-Related Signal Transduction. Front. Psychiatry.

[28] Love M.I., Huber W., Anders S. (2014). Moderated Estimation of Fold Change and Dispersion for RNA-Seq Data with DESeq2. Genome Biol..

[29] Talubo N.D.D., Tsai P.-W., Tayo L.L. (2024). Comprehensive RNA-Seq Gene Co-Expression Analysis Reveals Consistent Molecular Pathways in Hepatocellular Carcinoma across Diverse Risk Factors. Biology.

[30] De La Cerna J.L.O., Talubo N.D.D., Villanueva B.H.A., Tsai P.-W., Tayo L.L. (2025). Conserved Blood Transcriptome Patterns Highlight MicroRNA and Hub Gene Drivers of Neurodegeneration. Genes.

[31] Langfelder P., Horvath S. (2008). WGCNA: An R Package for Weighted Correlation Network Analysis. BMC Bioinform..

[32] Huang Y., Liu Y., Wu Y., Tang Y., Liu S., Xiao L., Zhang M., Tao S., Xie M., Dai M. (2021). Patterns of Convergence and Divergence between Bipolar Disorder Type I and Type II: Evidence from Integrative Genomic Analyses. Front. Cell Dev. Biol..

[33] Mullins N., Forstner A.J., O’Connell K.S., Coombes B., Coleman J.R.I., Qiao Z., Als T.D., Bigdeli T.B., Børte S., Bryois J. (2021). Genome-Wide Association Study of More than 40,000 Bipolar Disorder Cases Provides New Insights into the Underlying Biology. Nat. Genet..

[34] Zhang Z.-Q., Wu W.-W., Chen J.-D., Zhang G.-Y., Lin J.-Y., Wu Y.-K., Zhang Y., Su Y.-A., Li J.-T., Si T.-M. (2021). Weighted Gene Coexpression Network Analysis Reveals Essential Genes and Pathways in Bipolar Disorder. Front. Psychiatry.

[35] Garcia J.P.T., Tayo L.L. (2024). Codes between Poles: Linking Transcriptomic Insights into the Neurobiology of Bipolar Disorder. Biology.

[36] Fu Y., Zhou Q.-Z., Zhang X.-L., Wang Z.-Z., Wang P. (2019). Identification of Hub Genes Using Co-Expression Network Analysis in Breast Cancer as a Tool to Predict Different Stages. Med. Sci. Monit..

[37] Szklarczyk D., Kirsch R., Koutrouli M., Nastou K., Mehryary F., Hachilif R., Gable A.L., Fang T., Doncheva N.T., Pyysalo S. (2023). The STRING Database in 2023: Protein–Protein Association Networks and Functional Enrichment Analyses for Any Sequenced Genome of Interest. Nucleic Acids Res..

[38] Shannon P., Markiel A., Ozier O., Baliga N.S., Wang J.T., Ramage D., Amin N., Schwikowski B., Ideker T. (2003). Cytoscape: A Software Environment for Integrated Models of Biomolecular Interaction Networks. Genome Res..

[39] Yuan C., Hu Z., Shu X., Wang X., Jie Z. (2026). Causal Relationship between Metabolic Syndrome and Gastric Cancer: Insights from Comprehensive Analysis and Biomarker Identification. Transl. Cancer Res..

[40] Gandal M.J., Haney J.R., Parikshak N.N., Leppa V., Ramaswami G., Hartl C., Schork A.J., Appadurai V., Buil A., Werge T.M. (2018). Shared Molecular Neuropathology across Major Psychiatric Disorders Parallels Polygenic Overlap. Science.

[41] Akula N., Marenco S., Johnson K., Feng N., Zhu K., Schulmann A., Corona W., Jiang X., Cross J., England B. (2021). Deep Transcriptome Sequencing of Subgenual Anterior Cingulate Cortex Reveals Cross-Diagnostic and Diagnosis-Specific RNA Expression Changes in Major Psychiatric Disorders. Neuropsychopharmacology.

[42] Castro-Martínez J.A., Vargas E., Díaz-Beltrán L., Esteban F.J. (2024). Enhancing Transcriptomic Insights into Neurological Disorders Through the Comparative Analysis of Shapley Values. Curr. Issues Mol. Biol..

[43] Gandal M.J., Zhang P., Hadjimichael E., Walker R.L., Chen C., Liu S., Won H., van Bakel H., Varghese M., Wang Y. (2018). Transcriptome-Wide Isoform-Level Dysregulation in ASD, Schizophrenia, and Bipolar Disorder. Science.

[44] Ghandikota S., Sharma M., Jegga A.G. (2021). Computational Workflow for Functional Characterization of COVID-19 through Secondary Data Analysis. STAR Protoc..

[45] Buniello A., Suveges D., Cruz-Castillo C., Llinares M.B., Cornu H., Lopez I., Tsukanov K., Roldán-Romero J.M., Mehta C., Fumis L. (2025). Open Targets Platform: Facilitating Therapeutic Hypotheses Building in Drug Discovery. Nucleic Acids Res..

[46] Wijesooriya K., Jadaan S.A., Perera K.L., Kaur T., Ziemann M. (2022). Urgent Need for Consistent Standards in Functional Enrichment Analysis. PLoS Comput. Biol..

[47] Stelzer G., Rosen N., Plaschkes I., Zimmerman S., Twik M., Fishilevich S., Stein T.I., Nudel R., Lieder I., Mazor Y. (2016). The GeneCards Suite: From Gene Data Mining to Disease Genome Sequence Analyses. Curr. Protoc. Bioinform..

[48] Huo J., Wang L., Tian Y., Sun W., Zhang G., Zhang Y., Liu Y., Zhang J., Yang X., Liu Y. (2021). Gene Co-Expression Analysis Identified Preserved and Survival-Related Modules in Severe Blunt Trauma, Burns, Sepsis, and Systemic Inflammatory Response Syndrome. Int. J. Gen. Med..

[49] Ermakov E.A., Mednova I.A., Boiko A.S., Buneva V.N., Ivanova S.A. (2023). Chemokine Dysregulation and Neuroinflammation in Schizophrenia: A Systematic Review. Int. J. Mol. Sci..

[50] Stuart M.J., Baune B.T. (2014). Chemokines and Chemokine Receptors in Mood Disorders, Schizophrenia, and Cognitive Impairment: A Systematic Review of Biomarker Studies. Neurosci. Biobehav. Rev..

[51] Duan W., Huang G., Sui Y., Wang K., Yu Y., Chu X., Cao X., Chen L., Liu J., Eichler E.E. (2024). Deficiency of *DDX3X* Results in Neurogenesis Defects and Abnormal Behaviors via Dysfunction of the Notch Signaling. Proc. Natl. Acad. Sci. USA.

[52] Wang X., Yu S., Gao Z., Xu F., Wang Y., Zhang T., Xie T., Jia X. (2025). The Alteration of IL-17 Signaling Pathway in Bipolar Disorder: A Preliminary Study with Transcriptomic Perspective. Front. Psychiatry.

[53] Garcia J.P.T., Tayo L.L. (2024). Theoretical Studies of DNA Microarray Present Potential Molecular and Cellular Interconnectivity of Signaling Pathways in Immune System Dysregulation. Genes.

[54] Cadungog M.E.G.T., Tayo L.L. (2025). Microinflammation-Driven Gene Expression Dynamics in the Pathogenesis of Metabolic Disorders and Cancer. Biology.

[55] Li H., Sheng Z., Khan S., Zhang R., Liu Y., Zhang Y., Yong V.W., Xue M. (2022). Matrix Metalloproteinase-9 as an Important Contributor to the Pathophysiology of Depression. Front. Neurol..

[56] Mendez-Victoriano G., Zhu Y., Neuhaus L., Shaik S., Middleton F., Kondo Y., Fayyazuddin A., Hoeppner D., Puvogel S., Alsema A. (2026). Increased Tumor Necrosis Factor Superfamily Members in Neuroinflammatory Schizophrenia and Bipolar Disorder Midbrains. Biol. Psychiatry Glob. Open Sci..

[57] Zhou X., Song H., He J., Han W., Li Q. (2024). Deciphering Microglial Activation and Neuronal Apoptosis Post-traumatic Brain Injury: The Role of TYROBP in Inflammation Regulation Networks. Mol. Med. Rep..

[58] de Baumont A., Maschietto M., Lima L., Carraro D.M., Olivieri E.H., Fiorini A., Barreta L.A.N., Palha J.A., Belmonte-de-Abreu P., Moreira Filho C.A. (2015). Innate Immune Response Is Differentially Dysregulated between Bipolar Disease and Schizophrenia. Schizophr. Res..

[59] Su K.-P., Huang S.-Y., Peng C.-Y., Lai H.-C., Huang C.-L., Chen Y.-C., Aitchison K.J., Pariante C.M. (2010). Phospholipase A2 and Cyclooxygenase 2 Genes Influence the Risk of Interferon-α–Induced Depression by Regulating Polyunsaturated Fatty Acids Levels. Biol. Psychiatry.

[60] Zhang J., Chang L., Pu Y., Hashimoto K. (2020). Abnormal Expression of Colony Stimulating Factor 1 Receptor (CSF1R) and Transcription Factor PU.1 (SPI1) in the Spleen from Patients with Major Psychiatric Disorders: A Role of Brain–Spleen Axis. J. Affect. Disord..

[61] Ghena N., Anderson S.R., Roberts J.M., Irvin E., Schwakopf J., Bosco A., Vetter M.L. (2025). CD11c-Expressing Microglia Are Transient, Driven by Interactions with Apoptotic Cells. Glia.

[62] Ab Rajab N.S., Yasin M.A.M., Ghazali W.S.W., Talib N.A., Taib W.R.W., Sulong S. (2024). Schizophrenia and Rheumatoid Arthritis Genetic Scenery: Potential Non-HLA Genes Involved in Both Diseases Relationship. Yale J. Biol. Med..

[63] Ma Y., Liu Y., Ruan X., Liu X., Zheng J., Teng H., Shao L., Yang C., Wang D., Xue Y. (2021). Gene Expression Signature of Traumatic Brain Injury. Front. Genet..

[64] Li X., Qiao M., Zhou Y., Peng Y., Wen G., Xie C., Zhang Y. (2024). Modulating the RPS27A/PSMD12/NF-ΚB Pathway to Control Immune Response in Mouse Brain Ischemia-Reperfusion Injury. Mol. Med..

[65] Watanabe M., Toyomura T., Wake H., Nishinaka T., Hatipoglu O.F., Takahashi H., Nishibori M., Mori S. (2022). Identification of Ribosomal Protein L9 as a Novel Regulator of Proinflammatory Damage-Associated Molecular Pattern Molecules. Mol. Biol. Rep..

[66] Hwang I.K., Yoo K., Kim D.W., Kim S.Y., Park J.H., Ryoo Z.Y., Kim J., Choi S.Y., Won M. (2008). Ischemia-induced Ribosomal Protein S3 Expressional Changes and the Neuroprotective Effect against Experimental Cerebral Ischemic Damage. J. Neurosci. Res..

[67] Ketenci-İşlek S., Ürel-Demir G., Utine G.E., Şimşek-Kiper P.Ö. (2026). Two Siblings with a Homozygous EEF1B2 Loss-of-Function Variant: Expanding the Phenotypic Spectrum of EEF1B2-Related Neurodevelopmental Disorder. Neurogenetics.

[68] Rebouças R., Araújo-Pereira M., Mota T.F., Scoppetta H.Q.S., Rodrigues M.M.S., Andrade B.B., Fukutani E.R., de Queiroz A.T.L. (2025). Disrupted Genes and Pathways in Schizophrenia: A Robust Analysis of the Brain and Blood. BMC Psychiatry.

[69] Kong X., Liao Y., Zhou L., Zhang Y., Cheng J., Yuan Z., Wang S. (2020). Hematopoietic Cell Kinase (HCK) Is Essential for NLRP3 Inflammasome Activation and Lipopolysaccharide-Induced Inflammatory Response In Vivo. Front. Pharmacol..

[70] Liu Y., Yang H., Luo N., Fu Y., Qiu F., Pan Z., Li X., Jian W., Yang X., Xue Q. (2023). An Fgr Kinase Inhibitor Attenuates Sepsis-Associated Encephalopathy by Ameliorating Mitochondrial Dysfunction, Oxidative Stress, and Neuroinflammation via the SIRT1/PGC-1α Signaling Pathway. J. Transl. Med..

[71] Chu C.-H., Pan G.-Z., Tsai C.-Y., Yu C.-H.A., Lin H.-N., Chiang H.-L., Chen C., Chen L.-C., Pham X.-D.T., Lin C.-L. (2025). Nuclear Paxillin Functions as a Molecular Switch for Alternative Splicing in Neurons during a Critical Period of Brain Development. EMBO J..

[72] Nutma E., Fancy N., Weinert M., Tsartsalis S., Marzin M.C., Muirhead R.C.J., Falk I., Breur M., de Bruin J., Hollaus D. (2023). Translocator Protein Is a Marker of Activated Microglia in Rodent Models but Not Human Neurodegenerative Diseases. Nat. Commun..

[73] Snyder M., Huang X.-Y., Zhang J.J. (2011). Stat3 Is Essential for Neuronal Differentiation through Direct Transcriptional Regulation of the Sox6 Gene. FEBS Lett..

[74] Ohayon S., Yitzhaky A., Hertzberg L. (2020). Gene Expression Meta-Analysis Reveals the up-Regulation of CREB1 and CREBBP in Brodmann Area 10 of Patients with Schizophrenia. Psychiatry Res..

[75] Xiong J., Ding Y., Wu X., Zhan J., Wan Q., Wan H., Wei B., Chen H., Yang Y. (2024). Association between Serum Insulin-like Growth Factor 1 Levels and the Improvements of Cognitive Impairments in a Subgroup of Schizophrenia: Preliminary Findings. Schizophr. Res..

[76] Gong M., Shi R., Liu Y., Ke J., Liu X., Du H., Liu C. (2022). Abnormal Microglial Polarization Induced by *Arid1a* Deletion Leads to Neuronal Differentiation Deficits. Cell Prolif..

[77] von Knebel K., Staab J., Gregus A., Remling L., Wirths O., Spitzer C., Herrmann-Lingen C., Reichardt H.M., Meyer T. (2025). Social Inhibition in Depressed Patients Is Associated with an Altered Activation Profile of the Interleukin-6-Inducible Transcription Factor STAT3. Brain Behav. Immun. Health.

[78] Joseph D’Ercole A., Ye P. (2008). Expanding the Mind: Insulin-Like Growth Factor I and Brain Development. Endocrinology.

[79] Li L., Wang Q., Sun X., Li Z., Liu S., Zhang X., Zhou J., Zhang R., Liu K., Wang P. (2023). Activation of RhoA Pathway Participated in the Changes of Emotion, Cognitive Function and Hippocampal Synaptic Plasticity in Juvenile Chronic Stress Rats. Int. J. Biol. Macromol..

[80] Raivich G., Behrens A. (2006). Role of the AP-1 Transcription Factor c-Jun in Developing, Adult and Injured Brain. Prog. Neurobiol..

[81] Babaei P., Faraji N., Eyvani K. (2025). C-Fos Expression Differentially Acts in the Healthy Brain Compared with Alzheimer’s Disease. Gene Expr..

[82] Thiselton D.L., Vladimirov V.I., Kuo P.-H., McClay J., Wormley B., Fanous A., O’Neill F.A., Walsh D., Van den Oord E.J.C.G., Kendler K.S. (2008). AKT1 Is Associated with Schizophrenia Across Multiple Symptom Dimensions in the Irish Study of High Density Schizophrenia Families. Biol. Psychiatry.

[83] Karege F., Perroud N., Schürhoff F., Méary A., Marillier G., Burkhardt S., Ballmann E., Fernandez R., Jamain S., Leboyer M. (2010). Association of *AKT1* Gene Variants and Protein Expression in Both Schizophrenia and Bipolar Disorder. Genes Brain Behav..

[84] Cheng X., Xie Y., Wang A., Zhu C., Yan F., Pei W., Zhang X. (2023). Correlation between Elevated Serum Interleukin-1β, Interleukin-16 Levels and Psychiatric Symptoms in Patients with Schizophrenia at Different Stages. BMC Psychiatry.

[85] Liu F., Yang Y., Fan X.-W., Zhang N., Wang S., Shi Y.-J., Hu W.-J., Wang C.-X. (2024). Impacts of Inflammatory Cytokines on Depression: A Cohort Study. BMC Psychiatry.

[86] Saetre P., Emilsson L., Axelsson E., Kreuger J., Lindholm E., Jazin E. (2007). Inflammation-Related Genes up-Regulated in Schizophrenia Brains. BMC Psychiatry.

[87] De Masi R., Orlando S. (2020). IFI35 as a Biomolecular Marker of Neuroinflammation and Treatment Response in Multiple Sclerosis. Life Sci..

[88] Magusali N., Graham A.C., Piers T.M., Panichnantakul P., Yaman U., Shoai M., Reynolds R.H., Botia J.A., Brookes K.J., Guetta-Baranes T. (2021). A Genetic Link between Risk for Alzheimer’s Disease and Severe COVID-19 Outcomes via the *OAS1* Gene. Brain.

[89] De Masi R., Orlando S., Bagordo F., Grassi T. (2021). IFP35 Is a Relevant Factor in Innate Immunity, Multiple Sclerosis, and Other Chronic Inflammatory Diseases: A Review. Biology.

[90] Björvang R.D., Vrettou M., Bujanda Cundin X., Del Prete E., Rüegg J., Lager S., di Bernardo D., Comasco E., Skalkidou A. (2025). Differentially Expressed Transcripts Associated with Depressive Symptoms during Pregnancy and Postpartum. Mol. Psychiatry.

[91] Guo J., Ji Y., Ding Y., Jiang W., Sun Y., Lu B., Nagappan G. (2016). BDNF Pro-Peptide Regulates Dendritic Spines via Caspase-3. Cell Death Dis..

[92] Wu Y., Wang Z., Hu H., Wu T., Alabed A.A.A., Sun Z., Wang Y., Cui G., Cong W., Li C. (2024). Identification of Immune-Related Gene Signature in Schizophrenia. Actas Esp. Psiquiatr..

[93] Qu L. (2012). Neuronal Fc Gamma Receptor I as a Novel Mediator for IgG Immune Complex-Induced Peripheral Sensitization. Neural Regen. Res..

[94] Bobińska K., Gałecka E., Szemraj J., Gałecki P., Talarowska M. (2017). Is There a Link between TNF Gene Expression and Cognitive Deficits in Depression?. Acta Biochim. Pol..

[95] Koskuvi M., Pörsti E., Hewitt T., Räsänen N., Wu Y.-C., Trontti K., McQuade A., Kalyanaraman S., Ojansuu I., Vaurio O. (2024). Genetic Contribution to Microglial Activation in Schizophrenia. Mol. Psychiatry.

[96] Polsek D., Cash D., Veronese M., Ilic K., Wood T.C., Milosevic M., Kalanj-Bognar S., Morrell M.J., Williams S.C.R., Gajovic S. (2020). The Innate Immune Toll-like-Receptor-2 Modulates the Depressogenic and Anorexiolytic Neuroinflammatory Response in Obstructive Sleep Apnoea. Sci. Rep..

[97] Park S.J., Lee J.Y., Kim S.J., Choi S.-Y., Yune T.Y., Ryu J.H. (2015). Toll-like Receptor-2 Deficiency Induces Schizophrenia-like Behaviors in Mice. Sci. Rep..

[98] Wong H., Levenga J., LaPlante L., Keller B., Cooper-Sansone A., Borski C., Milstead R., Ehringer M., Hoeffer C. (2020). Isoform-Specific Roles for AKT in Affective Behavior, Spatial Memory, and Extinction Related to Psychiatric Disorders. eLife.

[99] Mailem R.C., Tayo L.L. (2022). Drug Repurposing Using Gene Co-Expression and Module Preservation Analysis in Acute Respiratory Distress Syndrome (ARDS), Systemic Inflammatory Response Syndrome (SIRS), Sepsis, and COVID-19. Biology.

[100] Pasamba E.C., Orda M.A., Villanueva B.H.A., Tsai P.-W., Tayo L.L. (2024). Transcriptomic Analysis of Hub Genes Reveals Associated Inflammatory Pathways in Estrogen-Dependent Gynecological Diseases. Biology.

[101] Li S., Lei Z., Sun T. (2023). The Role of MicroRNAs in Neurodegenerative Diseases: A Review. Cell Biol. Toxicol..

[102] Adly N.M., Khalifa D., Abdel-Ghany S., Sabit H. (2026). MicroRNAs as Biomarkers and Molecular Mediators of Cognitive Dysfunction in Schizophrenia. J. Neural Transm..

[103] Gaudet A.D., Fonken L.K., Watkins L.R., Nelson R.J., Popovich P.G. (2018). MicroRNAs: Roles in Regulating Neuroinflammation. Neurosci..

[104] Abdolahi S., Zare-Chahoki A., Noorbakhsh F., Gorji A. (2022). A Review of Molecular Interplay between Neurotrophins and MiRNAs in Neuropsychological Disorders. Mol. Neurobiol..

[105] Ramírez A.E., Gil-Jaramillo N., Tapias M.A., González-Giraldo Y., Pinzón A., Puentes-Rozo P.J., Aristizábal-Pachón A.F., González J. (2022). MicroRNA: A Linking between Astrocyte Dysfunction, Mild Cognitive Impairment, and Neurodegenerative Diseases. Life.

[106] Sadeghi Z., Malekzadeh M., Sharifi M., Hashemibeni B. (2025). The Role of MiR-16 and MiR-34a Family in the Regulation of Cancers: A Review. Heliyon.

[107] Peregud D., Pavlova O., Spektor V., Solovieva M., Nebogina K., Pavlov K. (2025). Serum MiR-16-5p and MiR-486-5p Levels Are Associated with Aggression, Depression, and Anxiety in the Context of Psychiatric Disorders with Suicide Attempts. Middle East Curr. Psychiatry.

[108] Jin R., Xu S., Lin X., Shen M. (2017). MiR-136 Controls Neurocytes Apoptosis by Regulating Tissue Inhibitor of Metalloproteinases-3 in Spinal Cord Ischemic Injury. Biomed. Pharmacother..

[109] Zhao C., Wang X.-B., Zhang Y.-H., Zhou Y.-M., Yin Q., Yao W.-C. (2018). MicroRNA-424 Inhibits Cell Migration, Invasion and Epithelial-Mesenchymal Transition in Human Glioma by Targeting KIF23 and Functions as a Novel Prognostic Predictor. Eur. Rev. Med. Pharmacol. Sci..

[110] Vuokila N., Aronica E., Korotkov A., van Vliet E.A., Nuzhat S., Puhakka N., Pitkänen A. (2020). Chronic Regulation of MiR-124-3p in the Perilesional Cortex after Experimental and Human TBI. Int. J. Mol. Sci..

[111] Tripathi A., Volsko C., Garcia J.P., Agirre E., Allan K.C., Tesar P.J., Trapp B.D., Castelo-Branco G., Sim F.J., Dutta R. (2019). Oligodendrocyte Intrinsic MiR-27a Controls Myelination and Remyelination. Cell Rep..

[112] Li K.-X., Li J.-R., Zuo S.-J., Li X., Chen X.-T., Xiao P.-Y., Li H.-T., Sun L., Qian T., Zhang H.-M. (2024). Identification of MiR-20b-5p as an Inhibitory Regulator in Cardiac Differentiation via TET2 and DNA Hydroxymethylation. Clin. Epigenet..

[113] Xu D., Guo Q. (2024). MiR-26a Improves Microglial Activation and Neuronal Apoptosis in a Rat Model of Cerebral Infarction by Regulating the TREM1-TLR4/MyD88/NF-ΚB Axis. Dev. Neurosci..

[114] Liu X., Zhao P., Du X., Hou J., Zhang G., Zhang W., Yang L., Chen Y. (2024). Let-7b-5p Promotes Triptolide-Induced Growth-Inhibiting Effects in Glioma by Targeting IGF1R. Naunyn. Schmiedebergs. Arch. Pharmacol..

[115] Schoof M., Launspach M., Holdhof D., Nguyen L., Engel V., Filser S., Peters F., Immenschuh J., Hellwig M., Niesen J. (2019). The Transcriptional Coactivator and Histone Acetyltransferase CBP Regulates Neural Precursor Cell Development and Migration. Acta Neuropathol. Commun..

[116] Hu X., Li J., Fu M., Zhao X., Wang W. (2021). The JAK/STAT Signaling Pathway: From Bench to Clinic. Signal Transduct. Target. Ther..

[117] Liu X., Dai S., Liu P., Liu C. (2021). Arid1a Regulates Neural Stem/Progenitor Cell Proliferation and Differentiation during Cortical Development. Cell Prolif..

[118] Andrews S.J., Das D., Anstey K.J., Easteal S. (2017). Association of *AKAP6* and *MIR2113* with Cognitive Performance in a Population-based Sample of Older Adults. Genes Brain Behav..

[119] Ahmadi M., Morshedzadeh F., Ghaderian S.M.H., Ghafouri-Fard S. (2024). Emerging Role of MiR-520a in Human Diseases. Pathol. Res. Pract..

[120] Zhou W., Huang G., Ye J., Jiang J., Xu Q. (2020). Protective Effect of MiR-340-5p against Brain Injury after Intracerebral Hemorrhage by Targeting PDCD4. Cerebrovasc. Dis..

[121] Lu J., Zhang M., Yang X., Cui T., Dai J. (2017). MicroRNA-548c-3p Inhibits T98G Glioma Cell Proliferation and Migration by Downregulating c-Myb. Oncol. Lett..

[122] Rodrigues B., Leitão R.A., Santos M., Trofimov A., Silva M., Inácio Â.S., Abreu M., Nobre R.J., Costa J., Cardoso A.L. (2025). MiR-186-5p Inhibition Restores Synaptic Transmission and Neuronal Network Activity in a Model of Chronic Stress. Mol. Psychiatry.

[123] Liu Q., Bao H., Zhang S., Li C., Sun G., Sun X., Fu T., Wang Y., Liang P. (2024). MicroRNA-522-3p Promotes Brain Metastasis in Non-Small Cell Lung Cancer by Targeting Tensin 1 and Modulating Blood-Brain Barrier Permeability. Exp. Cell Res..

[124] Chen X., Deng S., Lei Q., He Q., Ren Y., Zhang Y., Nie J., Lu W. (2020). MiR-7-5p Affects Brain Edema After Intracerebral Hemorrhage and Its Possible Mechanism. Front. Cell Dev. Biol..

[125] Korleski J., Sall S., Luly K.M., Johnson M.K., Johnson A.L., Khela H., Lal B., Taylor T., Ashby J.M., Alonso H. (2025). Multipronged SMAD Pathway Targeting by Lipophilic Poly(β-Amino Ester) MiR-590-3p NanomiRs Inhibits Mesenchymal Glioblastoma Growth and Prolongs Survival. Signal Transduct. Target. Ther..

[126] Hu G., Shi Z., Shao W., Xu B. (2022). MicroRNA-214–5p Involves in the Protection Effect of Dexmedetomidine against Neurological Injury in Alzheimer’s Disease via Targeting the Suppressor of Zest 12. Brain Res. Bull..

[127] Zhi T., Jiang K., Xu X., Yu T., Wu W., Nie E., Zhou X., Jin X., Zhang J., Wang Y. (2017). MicroRNA-520d-5p Inhibits Human Glioma Cell Proliferation and Induces Cell Cycle Arrest by Directly Targeting PTTG1. Am. J. Transl. Res..

[128] Chen L., Zhang W., Yan W., Han L., Zhang K., Shi Z., Zhang J., Wang Y., Li Y., Yu S. (2012). The Putative Tumor Suppressor MiR-524–5p Directly Targets Jagged-1 and Hes-1 in Glioma. Carcinogenesis.

[129] Fusar-Poli P., Solmi M., Brondino N., Davies C., Chae C., Politi P., Borgwardt S., Lawrie S.M., Parnas J., McGuire P. (2019). Transdiagnostic Psychiatry: A Systematic Review. World Psychiatry.

[130] Dalgleish T., Black M., Johnston D., Bevan A. (2020). Transdiagnostic Approaches to Mental Health Problems: Current Status and Future Directions. J. Consult. Clin. Psychol..

[131] Thylur D.S., Goldsmith D.R. (2022). Brick by Brick: Building a Transdiagnostic Understanding of Inflammation in Psychiatry. Harv. Rev. Psychiatry.

[132] Grotzinger A.D. (2021). Shared Genetic Architecture across Psychiatric Disorders. Psychol. Med..

[133] Magioncalda P., Martino M. (2022). A Unified Model of the Pathophysiology of Bipolar Disorder. Mol. Psychiatry.

[134] Nakamura T., Takata A. (2023). The Molecular Pathology of Schizophrenia: An Overview of Existing Knowledge and New Directions for Future Research. Mol. Psychiatry.

[135] Manuel M.T.A., Tayo L.L. (2023). Navigating the Gene Co-Expression Network and Drug Repurposing Opportunities for Brain Disorders Associated with Neurocognitive Impairment. Brain Sci..

[136] Sanfilippo C., Pinzone M.R., Cambria D., Longo A., Palumbo M., Di Marco R., Condorelli F., Nunnari G., Malaguarnera L., Di Rosa M. (2018). OAS Gene Family Expression Is Associated with HIV-Related Neurocognitive Disorders. Mol. Neurobiol..

[137] Barretto A.J.B., Orda M.A., Tsai P., Tayo L.L. (2024). Analysis of Modular Hub Genes and Therapeutic Targets across Stages of Non-Small Cell Lung Cancer Transcriptome. Genes.

[138] Orda M.A., Fowler P.M.P.T., Tayo L.L. (2024). Modular Hub Genes in DNA Microarray Suggest Potential Signaling Pathway Interconnectivity in Various Glioma Grades. Biology.

[139] Elsaid S., Rubin-Kahana D.S., Kloiber S., Kennedy S.H., Chavez S., Le Foll B. (2022). Neurochemical Alterations in Social Anxiety Disorder (SAD): A Systematic Review of Proton Magnetic Resonance Spectroscopic Studies. Int. J. Mol. Sci..

[140] Japanese Society of Neuropsychopharmacology, Japanese Society of Clinical Neuropsychopharmacology (2025). Guideline for Pharmacological Treatment of Schizophrenia 2022. Neuropsychopharmacol. Rep..

[141] Gao K., Oruc E.B., Koparal B. (2025). Pharmacological Monotherapy for Depressive Disorders: Current and Future—A Narrative Review. Medicina.

[142] Andreoli G., Kasch C., Lindsay C.E., Hofmann S.G. (2026). Pharmacological Strategies for Treating Social Anxiety Disorder in Adults: A Systematic Review of Studies Published since 2015. Expert Rev. Neurother..

[143] Fellendorf F.T., Caboni E., Paribello P., Pinna M., D’Aloja E., Carucci S., Pinna F., Reininghaus E.Z., Carpiniello B., Manchia M. (2023). Pharmacological Treatment of Bipolar Depression: A Review of Observational Studies. Pharmaceuticals.

[144] Geddes J.R., Miklowitz D.J. (2013). Treatment of Bipolar Disorder. Lancet.

[145] Shukla R., Hirpara D., Sagar K. (2024). Aman Rajpura Promising Advances in Schizophrenia Treatment. J. Pharma Insights Res..

[146] Iqbal A., Bokhari S.F.H., Rehman M.U., Faizan Sattar S.M., Bakht D., Dost W., Basit A. (2025). Gut-Brain Connection in Schizophrenia: A Narrative Review. World J. Psychiatry.

[147] Mehta I., Juneja K., Nimmakayala T., Bansal L., Pulekar S., Duggineni D., Ghori H.K., Modi N., Younas S. (2025). Gut Microbiota and Mental Health: A Comprehensive Review of Gut-Brain Interactions in Mood Disorders. Cureus.

[148] Mou Y., Du Y., Zhou L., Yue J., Hu X., Liu Y., Chen S., Lin X., Zhang G., Xiao H. (2022). Gut Microbiota Interact with the Brain Through Systemic Chronic Inflammation: Implications on Neuroinflammation, Neurodegeneration, and Aging. Front. Immunol..

[149] Villanueva B.H.A., Huang H.-Y., Tyan Y.-C., Lin P.-J., Li C.-W., Minh H., Tayo L.L., Chuang K.-P. (2024). Immune MRNA Expression and Fecal Microbiome Composition Change Induced by Djulis (*Chenopodium formosanum* Koidz.) Supplementation in Aged Mice: A Pilot Study. Medicina.

[150] Su B.-W., Li Y., Yang L.-Y., Yang H.-X., Wang W.-H., Ren H.-W., Bao Y.-N., Lao J.-Y., Luan Z.-L. (2025). The Role of the Microbiota-Gut-Brain Axis in Schizophrenia: An Immunological Perspective. Front. Immunol..

[151] Gao K., Mu C., Farzi A., Zhu W. (2020). Tryptophan Metabolism: A Link Between the Gut Microbiota and Brain. Adv. Nutr..

[152] Wang Y., Yuan X., Kang Y., Song X. (2021). Tryptophan-Kynurenine Pathway as a Novel Link between Gut Microbiota and Schizophrenia: A Review. Trop. J. Pharm. Res..

[153] Cheng J., Hu H., Ju Y., Liu J., Wang M., Liu B., Zhang Y. (2024). Gut Microbiota-Derived Short-Chain Fatty Acids and Depression: Deep Insight into Biological Mechanisms and Potential Applications. Gen. Psychiatr..

[154] Ritz N.L., Brocka M., Butler M.I., Cowan C.S.M., Barrera-Bugueño C., Turkington C.J.R., Draper L.A., Bastiaanssen T.F.S., Turpin V., Morales L. (2024). Social Anxiety Disorder-Associated Gut Microbiota Increases Social Fear. Proc. Natl. Acad. Sci. USA.

[155] Stevenson A.J., McCartney D.L., Gadd D.A., Shireby G., Hillary R.F., King D., Tzioras M., Wrobel N., McCafferty S., Murphy L. (2022). A Comparison of Blood and Brain-derived Ageing and Inflammation-related DNA Methylation Signatures and Their Association with Microglial Burdens. Eur. J. Neurosci..

[156] Li Y., Chen J.A., Sears R.L., Gao F., Klein E.D., Karydas A., Geschwind M.D., Rosen H.J., Boxer A.L., Guo W. (2014). An Epigenetic Signature in Peripheral Blood Associated with the Haplotype on 17q21.31, a Risk Factor for Neurodegenerative Tauopathy. PLoS Genet..

[157] Irmady K., Hale C.R., Qadri R., Fak J., Simelane S., Carroll T., Przedborski S., Darnell R.B. (2023). Blood Transcriptomic Signatures Associated with Molecular Changes in the Brain and Clinical Outcomes in Parkinson’s Disease. Nat. Commun..

[158] Janigro D., Bailey D.M., Lehmann S., Badaut J., O’Flynn R., Hirtz C., Marchi N. (2021). Peripheral Blood and Salivary Biomarkers of Blood–Brain Barrier Permeability and Neuronal Damage: Clinical and Applied Concepts. Front. Neurol..

[159] Inamdar A., Bugadannavar P., Palled M., Umarani S., Salve P., Gurupadayya B., Patil P., Sharma H. (2025). Biological Determinants of Blood-Based Biomarker Levels in Alzheimer’s Disease: Role of Nutrition, Inflammation, and Metabolic Factors. Front. Aging Neurosci..

[160] Malpetti M., Swann P., Tsvetanov K.A., Chouliaras L., Strauss A., Chikaura T., Murley A.G., Ashton N.J., Barker P., Jones P.S. (2025). Blood Inflammation Relates to Neuroinflammation and Survival in Frontotemporal Lobar Degeneration. Brain.

[161] Hoseth E.Z., Ueland T., Dieset I., Birnbaum R., Shin J.H., Kleinman J.E., Hyde T.M., Mørch R.H., Hope S., Lekva T. (2017). A Study of TNF Pathway Activation in Schizophrenia and Bipolar Disorder in Plasma and Brain Tissue. Schizophr. Bull..

[162] Yang L., Wang M., Guo Y.Y., Sun T., Li Y.J., Yang Q., Zhang K., Liu S.B., Zhao M.G., Wu Y.M. (2016). Systemic Inflammation Induces Anxiety Disorder through CXCL12/CXCR4 Pathway. Brain Behav. Immun..

[163] Milenkovic V.M., Stanton E.H., Nothdurfter C., Rupprecht R., Wetzel C.H. (2019). The Role of Chemokines in the Pathophysiology of Major Depressive Disorder. Int. J. Mol. Sci..

[164] Kim S.J., Lee H.J., Koo H.G., Kim J.W., Song J.Y., Kim M.K., Shin D.H., Jin S.Y., Hong M.S., Park H.J. (2004). Impact of IL-1 Receptor Antagonist Gene Polymorphism on Schizophrenia and Bipolar Disorder. Psychiatr. Genet..

[165] Zhang B., Hu Y.-B., Li G., Yu H.-X., Cui C., Han Y.-Y., Li H.-X., Li G. (2024). Itga5-PTEN Signaling Regulates Striatal Synaptic Strength and Motor Coordination in Parkinson’s Disease. Int. J. Biol. Sci..

[166] Miguel-Hidalgo J.J., Overholser J.C., Jurjus G.J., Meltzer H.Y., Dieter L., Konick L., Stockmeier C.A., Rajkowska G. (2011). Vascular and Extravascular Immunoreactivity for Intercellular Adhesion Molecule 1 in the Orbitofrontal Cortex of Subjects with Major Depression: Age-Dependent Changes. J. Affect. Disord..

[167] Chen H.-J.C., Spiers J.G., Lerskiatiphanich T., Parker S.E., Lavidis N.A., Fung J.N., Woodruff T.M., Lee J.D. (2024). Complement C5a Receptor Signaling Alters Stress Responsiveness and Modulates Microglia Following Chronic Stress Exposure. Biol. Psychiatry Glob. Open Sci..

[168] Gao H., Zhang Z., Deng J., Song Y. (2025). Cathepsin S: Molecular Mechanisms in Inflammatory and Immunological Processes. Front. Immunol..

[169] Shen X., Wen Z., Deng S., Qiu Y., Ma W., Dong X., Gong J., Zhang Y., Liu D., Xu B. (2025). Regulation of Hindbrain Vascular Development by *Rps20* in Zebrafish. Cells.

[170] Blazejewski S.M., Bennison S.A., Ha N.T., Liu X., Smith T.H., Dougherty K.J., Toyo-Oka K. (2022). Rpsa Signaling Regulates Cortical Neuronal Morphogenesis via Its Ligand, PEDF, and Plasma Membrane Interaction Partner, Itga6. Cereb. Cortex.

[171] Pickard M.R., Mourtada-Maarabouni M., Williams G.T. (2011). Candidate Tumour Suppressor Fau Regulates Apoptosis in Human Cells: An Essential Role for Bcl-G. Biochim. Biophys. Acta (BBA)—Mol. Basis Dis..

[172] Smagin D.A., Kovalenko I.L., Galyamina A.G., Bragin A.O., Orlov Y.L., Kudryavtseva N.N. (2016). Dysfunction in Ribosomal Gene Expression in the Hypothalamus and Hippocampus Following Chronic Social Defeat Stress in Male Mice as Revealed by RNA-Seq. Neural Plast..

[173] Li Y.-H., Wang J., Liu Y., Qiu L., Li J.-Z., Hu H.-G., Hu Z.-L., Zhang W., Lu B., Zhang J.-P. (2018). Esculentoside A Specifically Binds to Ribosomal Protein S3a and Impairs LPS-Induced Signaling in Macrophages. Int. Immunopharmacol..

[174] Sundaresh A., Meistermann D., Lampela R., Yang Z., Woldegebriel R., Ganna A., Puigdevall Costa P., Kilpinen H. (2025). Joint Profiling of Cell Morphology and Gene Expression during in Vitro Neurodevelopment. eLife.

[175] Kim T.-H., Leslie P., Zhang Y. (2014). Ribosomal Proteins as Unrevealed Caretakers for Cellular Stress and Genomic Instability. Oncotarget.

[176] Martins de Almeida J.F., Contestabile M., Tonazzini I., De Cesari C., Baroncelli L., Martini C., Daniele S. (2025). Dysfunction of the Autophagy System and MDM2–P53 Axis Leads to the Accumulation of Amyloidogenic Proteins in Angelman Syndrome Models. Int. J. Mol. Sci..

[177] Fancello L., Kampen K.R., Hofman I.J.F., Verbeeck J., De Keersmaecker K. (2017). The Ribosomal Protein Gene RPL5 Is a Haploinsufficient Tumor Suppressor in Multiple Cancer Types. Oncotarget.

[178] Roman K.M., Jenkins A.K., Lewis D.A., Volk D.W. (2021). Involvement of the Nuclear Factor-ΚB Transcriptional Complex in Prefrontal Cortex Immune Activation in Bipolar Disorder. Transl. Psychiatry.

[179] Yang A., He G., Song Y., Wen Y., Xia H., Gu S. (2025). Nfkbia-Driven Neuroinflammatory Pathways Mediate Depression Following Spinal Cord Injury. Front. Mol. Neurosci..

[180] Pandey G.N., Rizavi H.S., Bhaumik R., Zhang H. (2021). Chemokines Gene Expression in the Prefrontal Cortex of Depressed Suicide Victims and Normal Control Subjects. Brain Behav. Immun..

[181] Fuchsova B., Alvarez Juliá A., Rizavi H.S., Frasch A.C., Pandey G.N. (2016). Expression of P21-Activated Kinases 1 and 3 Is Altered in the Brain of Subjects with Depression. Neuroscience.

[182] Ashbrook D.G., Cahill S., Hager R. (2019). A Cross-Species Systems Genetics Analysis Links APBB1IP as a Candidate for Schizophrenia and Prepulse Inhibition. Front. Behav. Neurosci..

[183] Wang P.-S., Chou F.-S., Ramachandran S., Xia S., Chen H.-Y., Guo F., Suraneni P., Maher B.J., Li R. (2016). Crucial Roles of the Arp2/3 Complex during Mammalian Corticogenesis. Development.

[184] Alp A., Özçelik Eroğlu E., Yıldız M.İ., Ceylan A.C., Demir B., Özer S. (2023). c.4168G>A(p.Ala 1390Thr) Variation in KMT2D Gene Detected in an Ultra-treatment-resistant Schizophrenia Patient: A Case Report and Literature Review. Arch. Neuropsychiatry.

[185] Lepri F., Cocciadiferro D., Augello B., Alfieri P., Pes V., Vancini A., Caciolo C., Squeo G., Malerba N., Adipietro I. (2017). Clinical and Neurobehavioral Features of Three Novel Kabuki Syndrome Patients with Mosaic KMT2D Mutations and a Review of Literature. Int. J. Mol. Sci..

[186] Halder S.K., Rafi M.O., Shahriar E.B., Albogami S., El-Shehawi A.M., Daullah S.M.M.U., Himel M.K., Emran T. (2022). Bin Identification of the Most Damaging NsSNPs in the Human CFL1 Gene and Their Functional and Structural Impacts on Cofilin-1 Protein. Gene.

[187] Dong L., Li Y., An H., Wang Y., Chen S., Qian Y., Wang K., Zhen J., Fan Z., Gong X. (2016). The E3 Ubiquitin Ligase C-Cbl Inhibits Microglia-Mediated CNSInflammation by Regulating PI3K/AktNF-*κ*B Pathway. CNS Neurosci. Ther..

[188] Sheikh M.A., O’Connell K.S., Lekva T., Szabo A., Akkouh I.A., Osete J.R., Agartz I., Engh J.A., Andreou D., Boye B. (2023). Systemic Cell Adhesion Molecules in Severe Mental Illness: Potential Role of Intercellular CAM-1 in Linking Peripheral and Neuroinflammation. Biol. Psychiatry.

[189] Ni C., Chen H., Chen Q., Liao Y., Wang Y., Ye L., Wu X., Ni H., Jiang T., Li S. (2025). Allele-Specific Regulation of PAXIP1-AS1 by SMC3/CEBPB at Rs112651172 in Psychiatric Disorders Drives Synaptic and Behavioral Dysfunctions in Mice. Adv. Sci..

[190] Ni C., Jiang W., Wang Z., Wang Z., Zhang J., Zheng X., Liu Z., Ou H., Jiang T., Liang W. (2021). LncRNA-AC006129.1 Reactivates a SOCS3-Mediated Anti-Inflammatory Response through DNA Methylation-Mediated CIC Downregulation in Schizophrenia. Mol. Psychiatry.

[191] Yu S., Gan C., Li W., Zhang Q., Cai Y., Xu J., Huang R., Yao S., Cheng L., Cheng H. (2025). Depression Decreases Immunity and PD-L1 Inhibitor Efficacy via the Hypothalamic–Pituitary–Adrenal (HPA) Axis in Triple-Negative Breast Cancer. Biochim. Biophys. Acta (BBA)—Mol. Basis Dis..

[192] Sannino G., Pasqualini L., Ricciardelli E., Montilla P., Soverchia L., Ruggeri B., Falcinelli S., Renzi A., Ludka C., Kirchner T. (2016). Acute Stress Enhances the Expression of Neuroprotection- and Neurogenesis-Associated Genes in the Hippocampus of a Mouse Restraint Model. Oncotarget.

[193] Tripathi A., Whitehead C., Surrao K., Pillai A., Madeshiya A., Li Y., Khodadadi H., Ahmed A.O., Turecki G., Baban B. (2021). Type 1 Interferon Mediates Chronic Stress-Induced Neuroinflammation and Behavioral Deficits via Complement Component 3-Dependent Pathway. Mol. Psychiatry.

[194] Fries G.R., Khan S., Stamatovich S., Dyukova E., Walss-Bass C., Lane S.D., Schmitz J.M., Wardle M.C. (2018). Anhedonia in Cocaine Use Disorder Is Associated with Inflammatory Gene Expression. PLoS ONE.

