# Supplementary material for: Peripheral Transcriptomic Signatures Reveal Convergent Neuroinflammatory, Metabolic, and miRNA Dysregulation in Major Psychiatric Disorders
Source: Biology (Basel). 2026 Apr 24;15(9):673. doi: 10.3390/biology15090673 (PMC13162896; doi:10.3390/biology15090673)
Supplement: Supplementary file 1 [file biology-15-00673-s001.zip › Supplementary Files/Supplementary Figures.pdf]

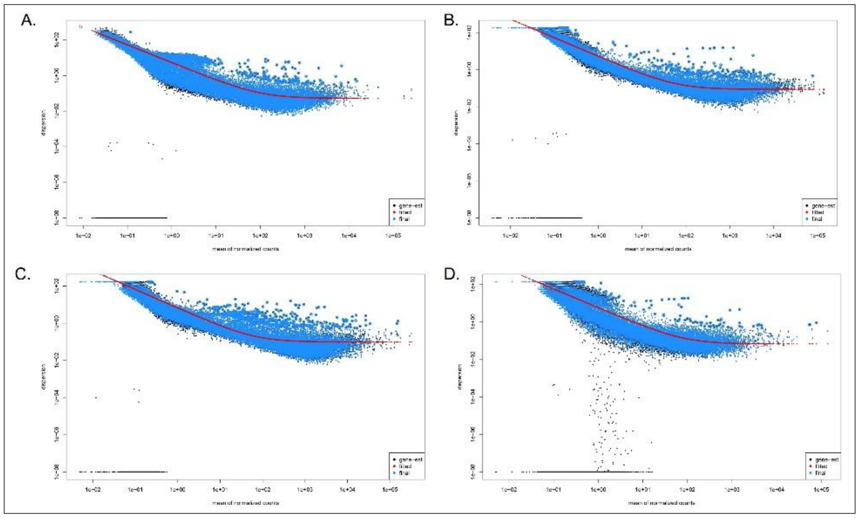

Figure S1. *Dispersion Estimates of RNA-Seq Data in neuropsychiatric disorders: (**A**)BP (**B**)SZ (**C**)SAD (**D**).*

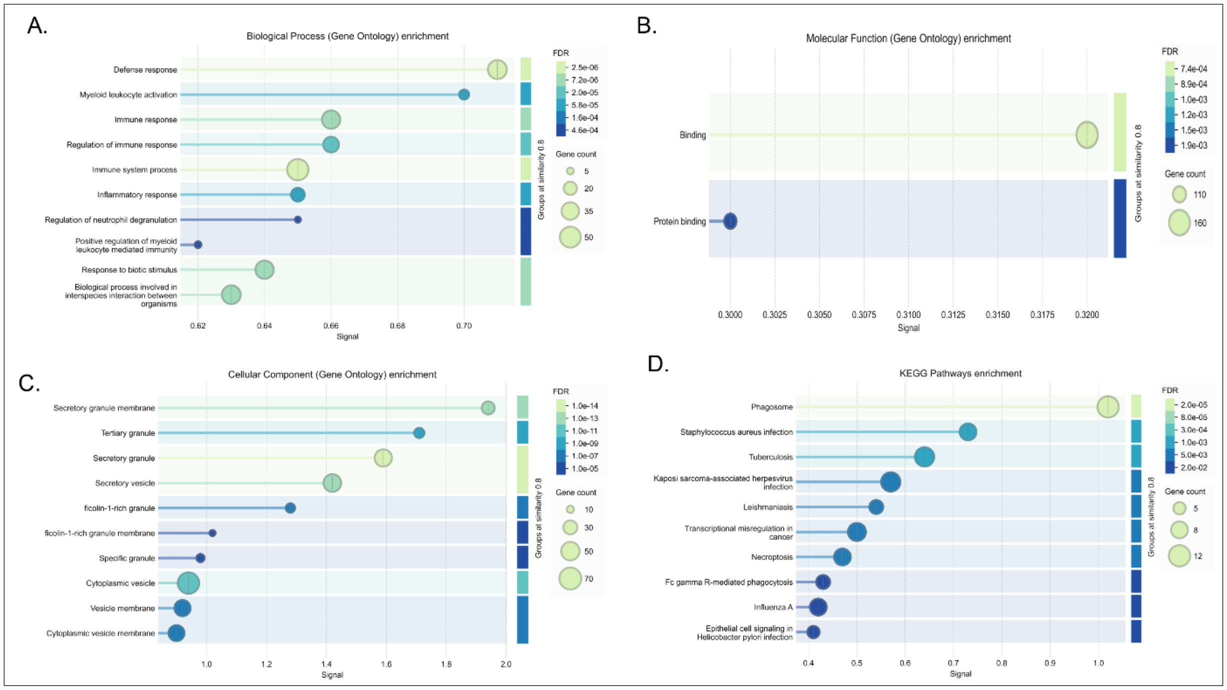

Figure S2. *Red module functional annotation and GO enrichment analysis. (**A**) GO BP, (**B**) GO MF, (**C**) GO CC and (**D**) KEGG.*

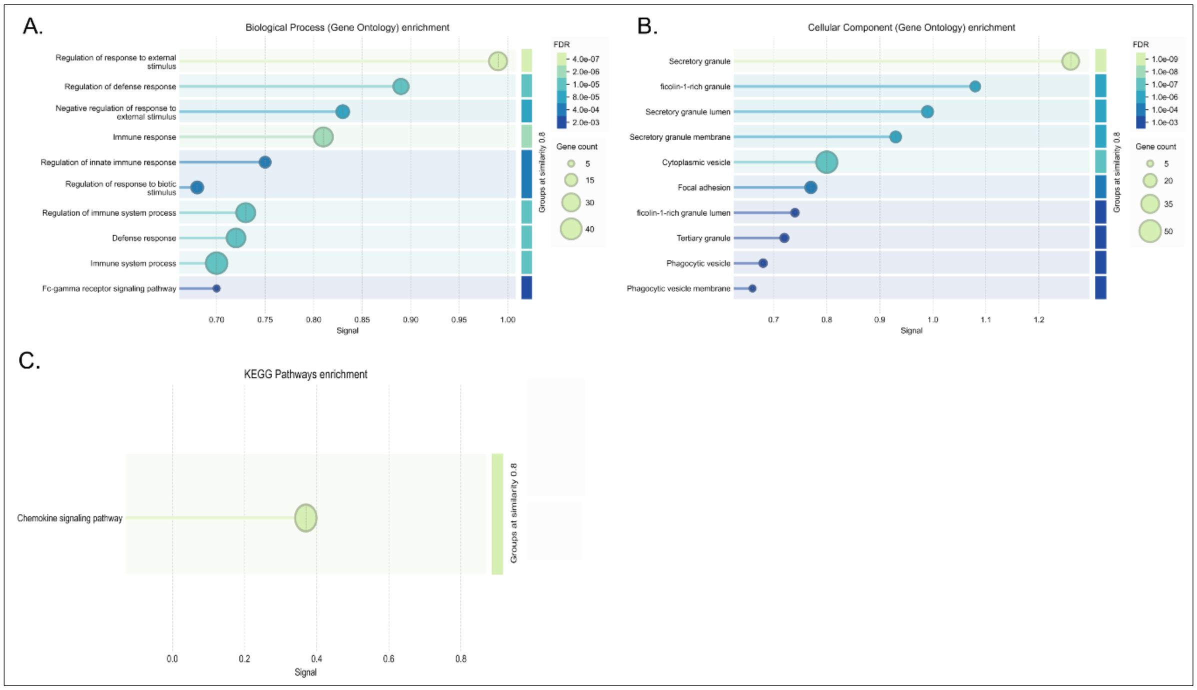

Figure S3. *Green-yellow module functional annotation and GO enrichment analysis. (**A**) GO BP, (**B**) GO CC and (**C**) KEGG.*

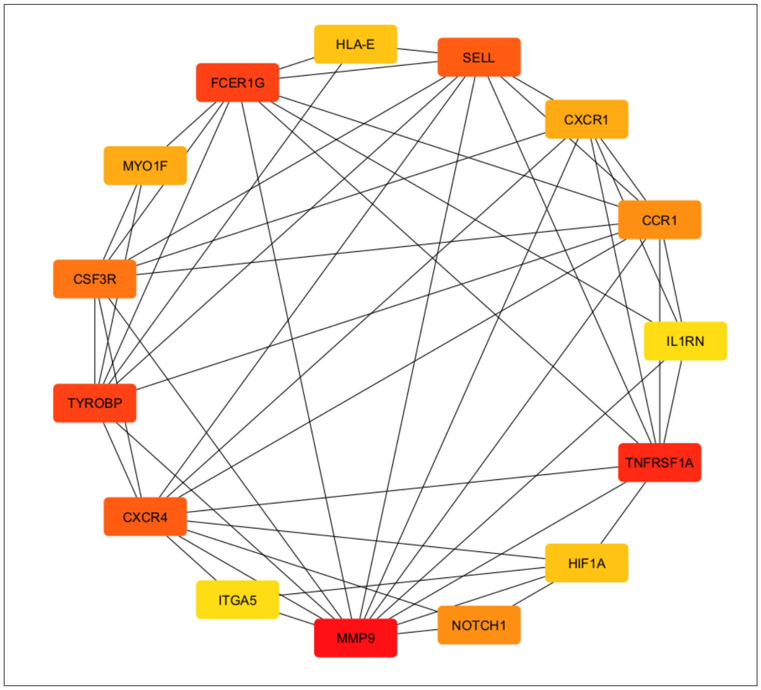

Figure S4. *Top 15 hub genes in green module.*

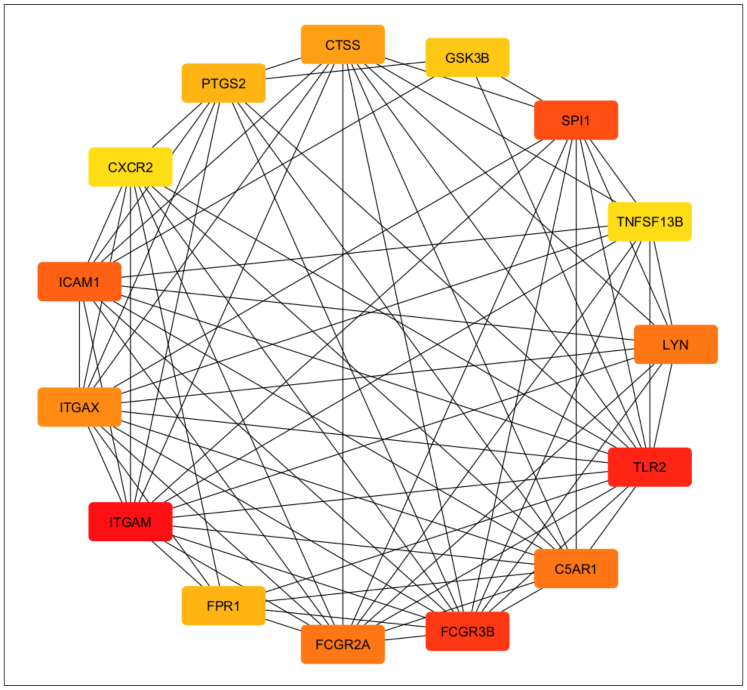

Figure S5. *Top 15 hub genes in Red module.*

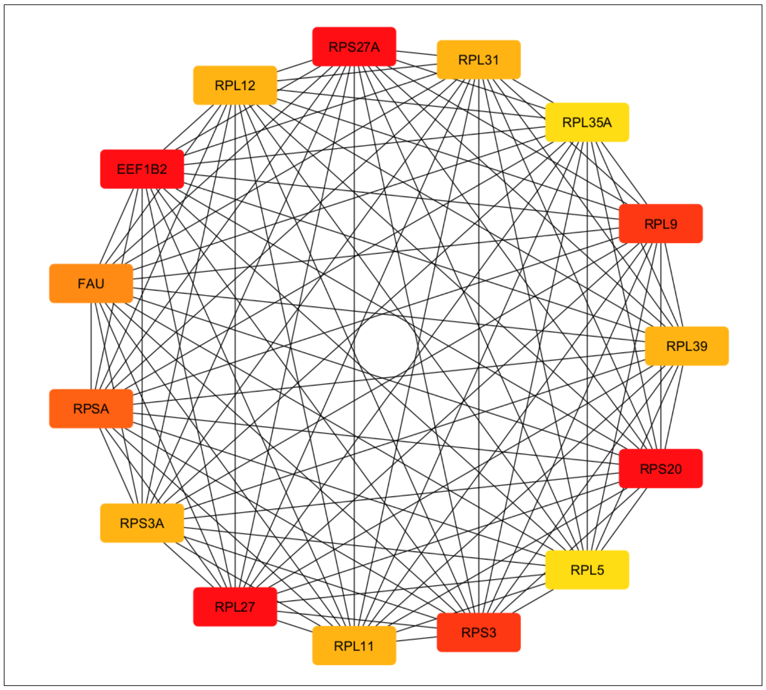

Figure S6. *Top 15 hub genes in Pink module.*

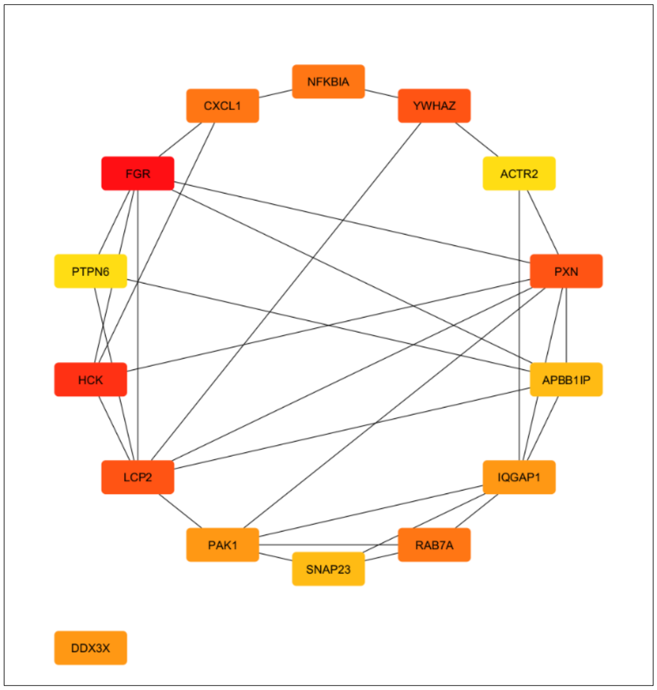

Figure S7. *Top 15 hub genes in Green-yellow module*

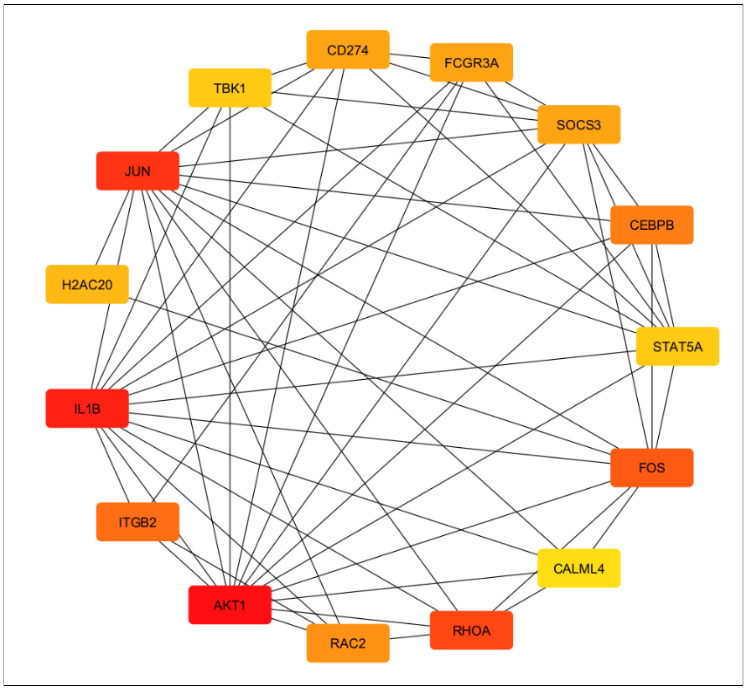

Figure S8*. Top 15 hub genes in yellow module.*

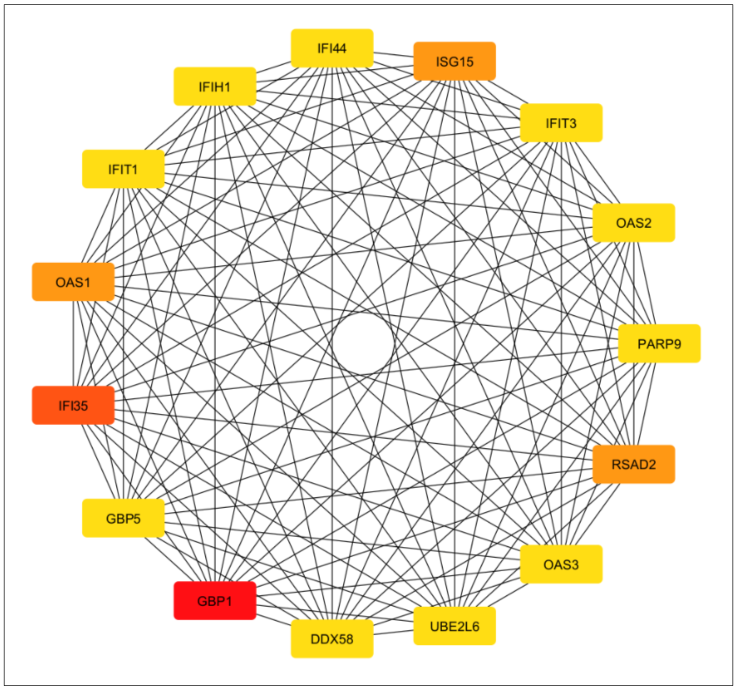

Figure S9. *Top 15 hub genes in Cyan module.*

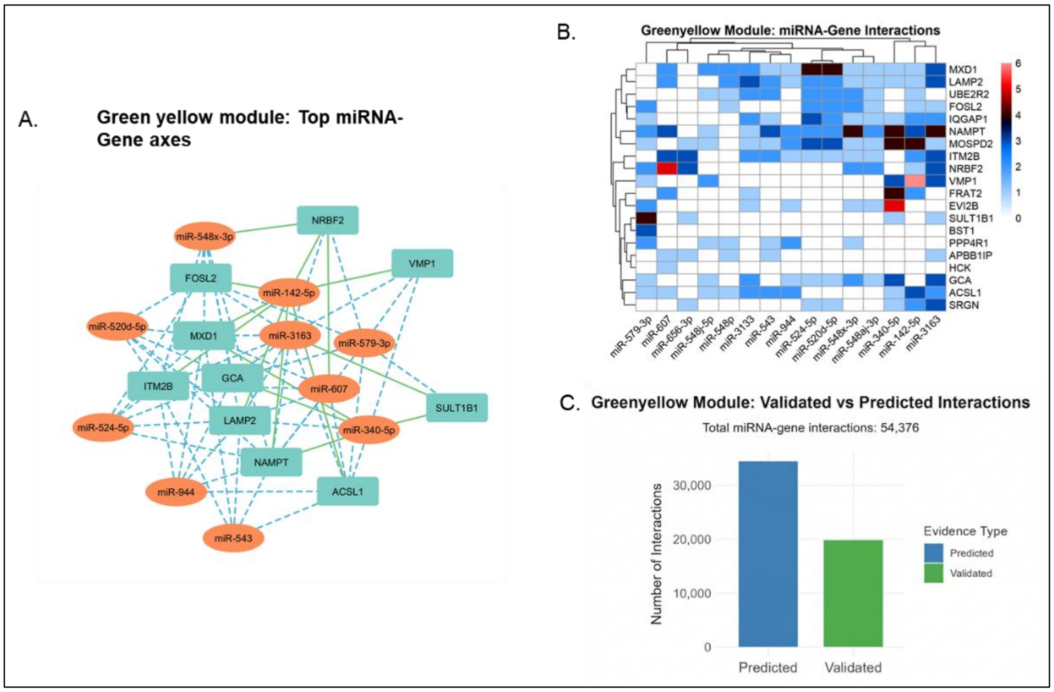

Figure S10. *miRNA–gene interaction profiles of the green-yellow module: (**A**) Network visualization depicting the top miRNA–gene regulatory axes. To explicitly separate evidence types, experimentally validated interactions are denoted by solid green edges, while computationally predicted interactions are denoted by dashed blue edges. (**B**) Heatmap of the top-ranked miRNAs and their interactions with green yellow module genes, illustrating relative connectivity patterns across the module. (**C**) Distribution of validated versus predicted interactions, depciting the relative contributions of experimentally supported and computationally inferred edges. Note: Interactions denoted as predicted (blue edges) are derived computationally from the multiMiR database and currently lack direct experimental validation; these axes should be interpreted with appropriate caution as inferred post-transcriptional models*

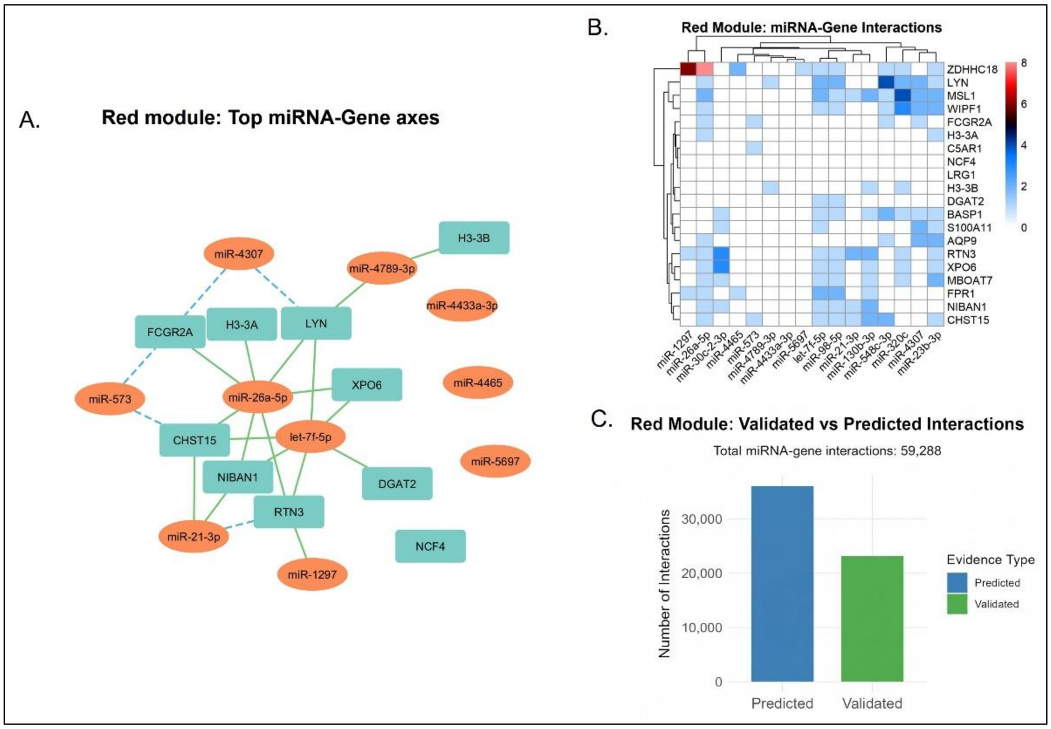

Figure S11. *miRNA–gene interaction profiles of the red module: (**A**) Network visualization depicting the top miRNA–gene regulatory axes. To explicitly separate evidence types, experimentally validated interactions are denoted by solid green edges, while computationally predicted interactions are denoted by dashed blue edges. (**B**) Heatmap of the top-ranked miRNAs and their interactions with red module genes, illustrating relative connectivity patterns across the module. (**C**) Distribution of validated versus predicted interactions, depciting the relative contributions of experimentally supported and computationally inferred edges. Note: Interactions denoted as predicted (blue edges) are derived computationally from the multiMiR database and currently lack direct experimental validation; these axes should be interpreted with appropriate caution as inferred post-transcriptional models*

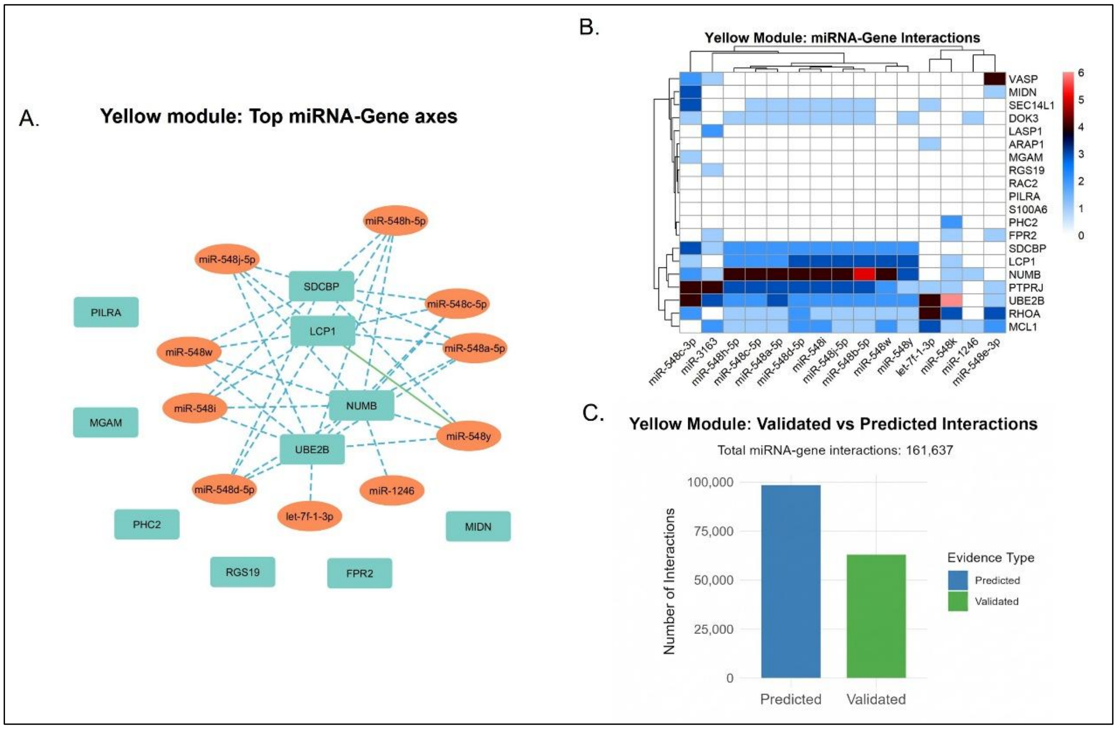

Figure S12. *miRNA–gene interaction profiles of the yellow module: (**A**) Network visualization depicting the top miRNA–gene regulatory axes. To explicitly separate evidence types, experimentally validated interactions are denoted by solid green edges, while computationally predicted interactions are denoted by dashed blue edges. (**B**) Heatmap of the top-ranked miRNAs and their interactions with yellow module genes, illustrating relative connectivity patterns across the module. (**C**) Distribution of validated versus predicted interactions, depciting the relative contributions of experimentally supported and computationally inferred edges. Note: Interactions denoted as predicted (blue edges) are derived computationally from the multiMiR database and currently lack direct experimental validation; these axes should be interpreted with appropriate caution as inferred post-transcriptional models*

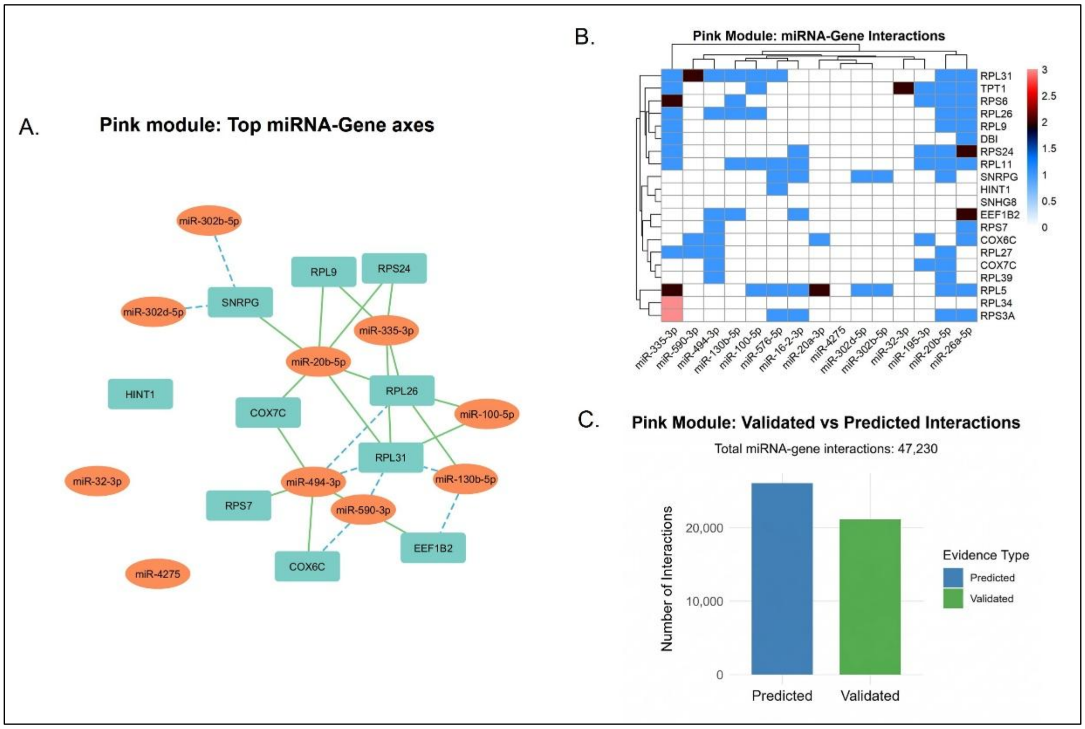

Figure S13. *miRNA–gene interaction profiles of the pink module: (**A**) Network visualization depicting the top miRNA–gene regulatory axes. To explicitly separate evidence types, experimentally validated interactions are denoted by solid green edges, while computationally predicted interactions are denoted by dashed blue edges. (**B**) Heatmap of the top-ranked miRNAs and their interactions with pink module genes, illustrating relative connectivity patterns across the module. (**C**) Distribution of validated versus predicted interactions, depciting the relative contributions of experimentally supported and computationally inferred edges. Note: Interactions denoted as predicted (blue edges) are derived computationally from the multiMiR database and currently lack direct experimental validation; these axes should be interpreted with appropriate caution as inferred post-transcriptional models*

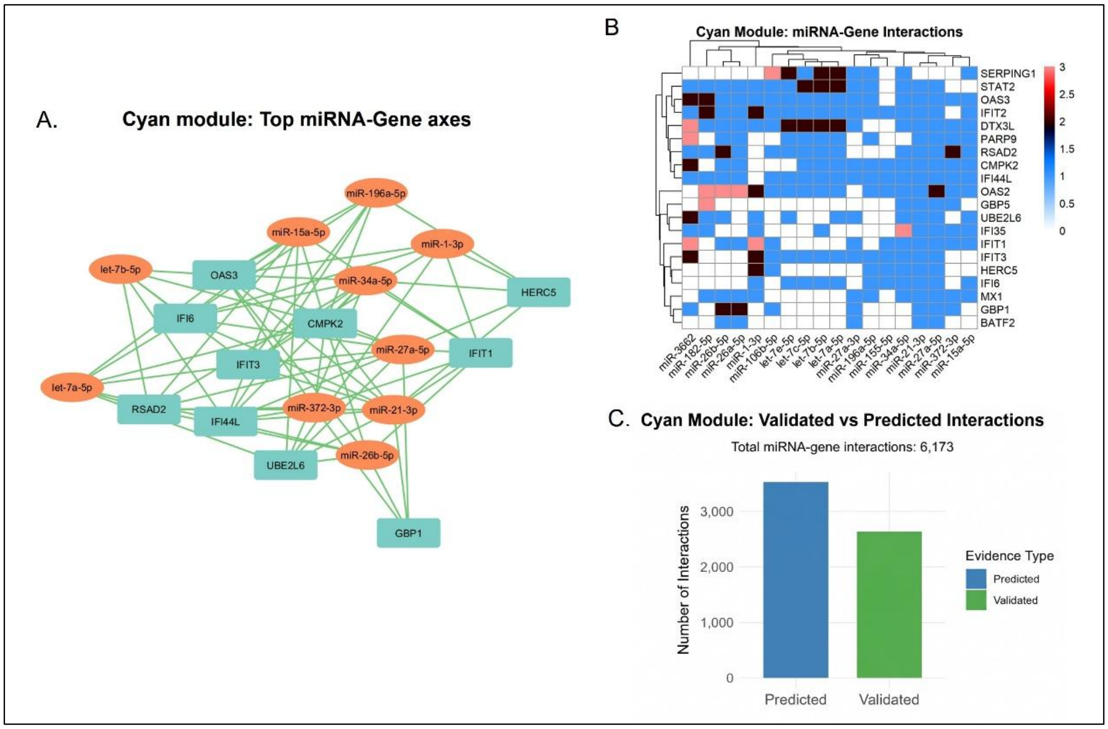

Figure S14. *miRNA–gene interaction profiles of the cyan module: (**A**) Network visualization depicting the top miRNA–gene regulatory axes. To explicitly separate evidence types, experimentally validated interactions are denoted by solid green edges, while computationally predicted interactions are denoted by dashed blue edges. (**B**) Heatmap of the top-ranked miRNAs and their interactions with cyan module genes, illustrating relative connectivity patterns across the module. (**C**) Distribution of validated versus predicted interactions, depciting the relative contributions of experimentally supported and computationally inferred edges. Note: Interactions denoted as predicted (blue edges) are derived computationally from the multiMiR database and currently lack direct experimental validation; these axes should be interpreted with appropriate caution as inferred post-transcriptional models*

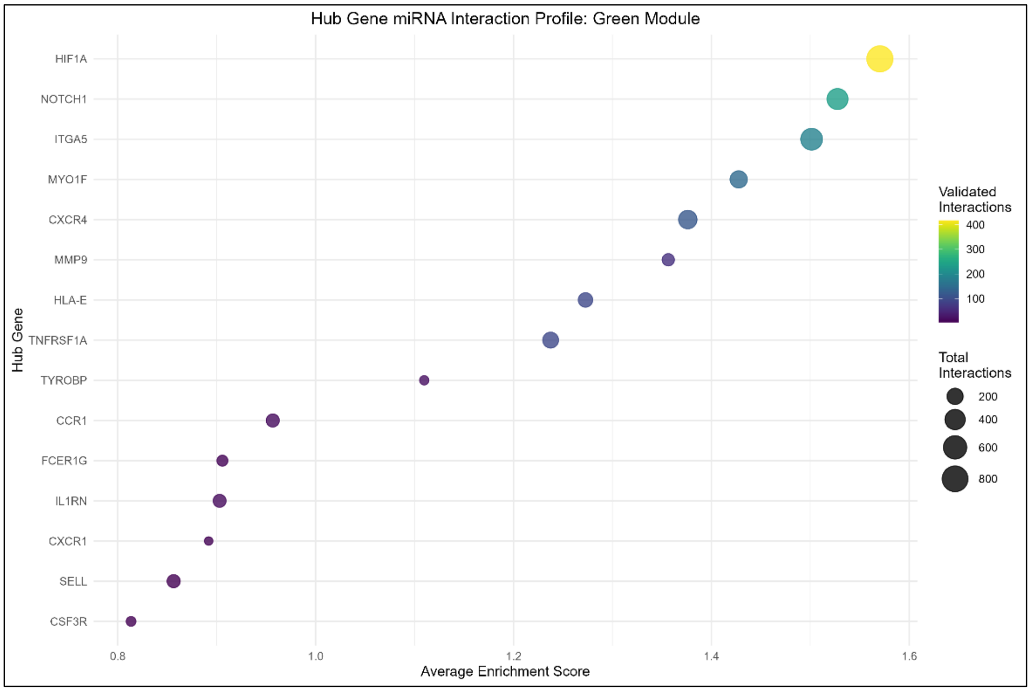

Figure S15. *Hub gene-miRNA interaction profile for the green module.*

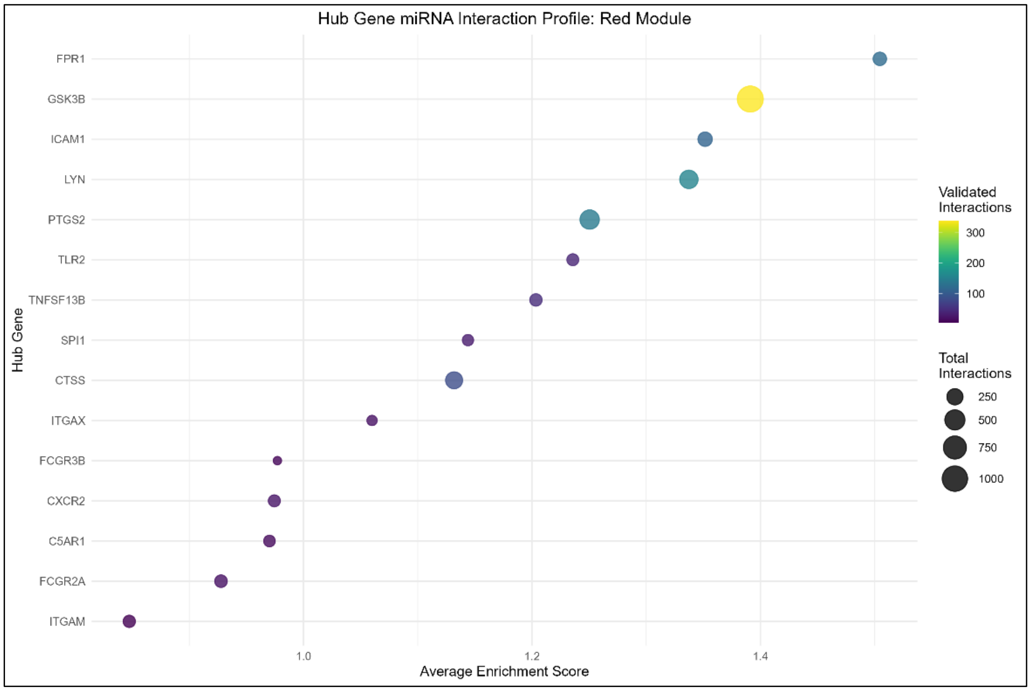

Figure S16. *Hub gene-miRNA interaction profile for the red module.*

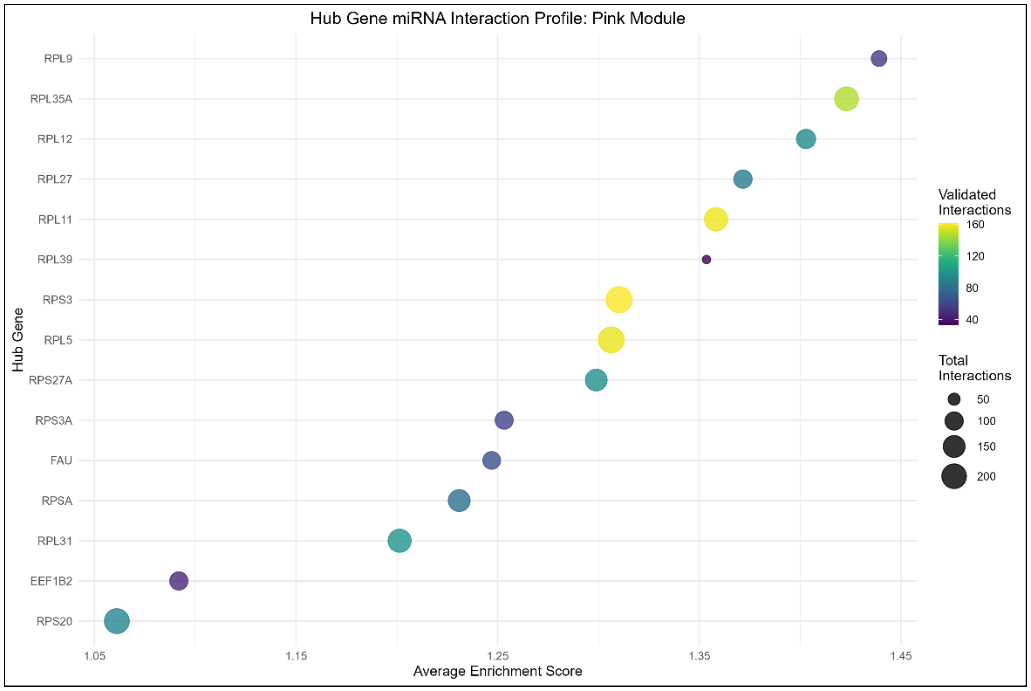

Figure S17. *Hub gene-miRNA interaction profile for the pink module.*

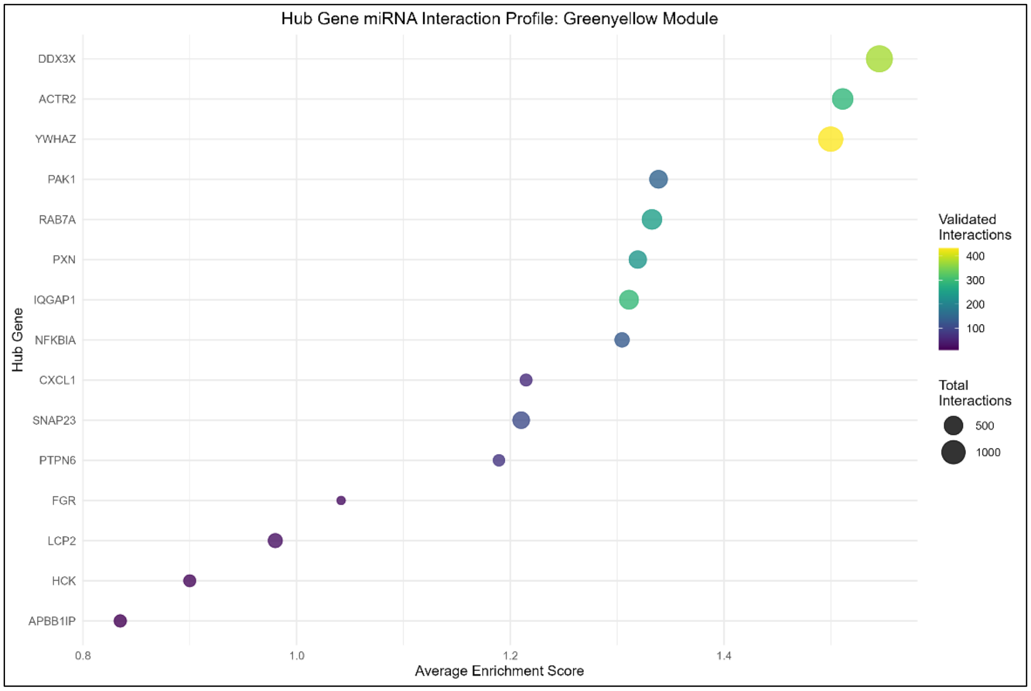

Figure S18. *Hub gene-miRNA interaction profile for the green-yellow module.*

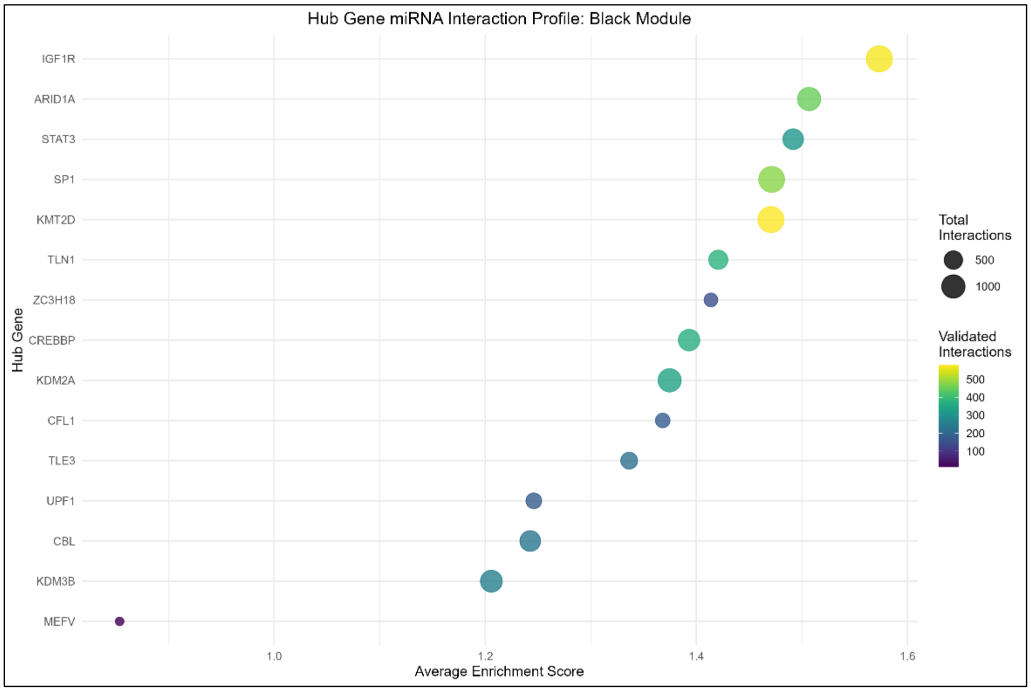

Figure S19. *Hub gene-miRNA interaction profile for the black module.*

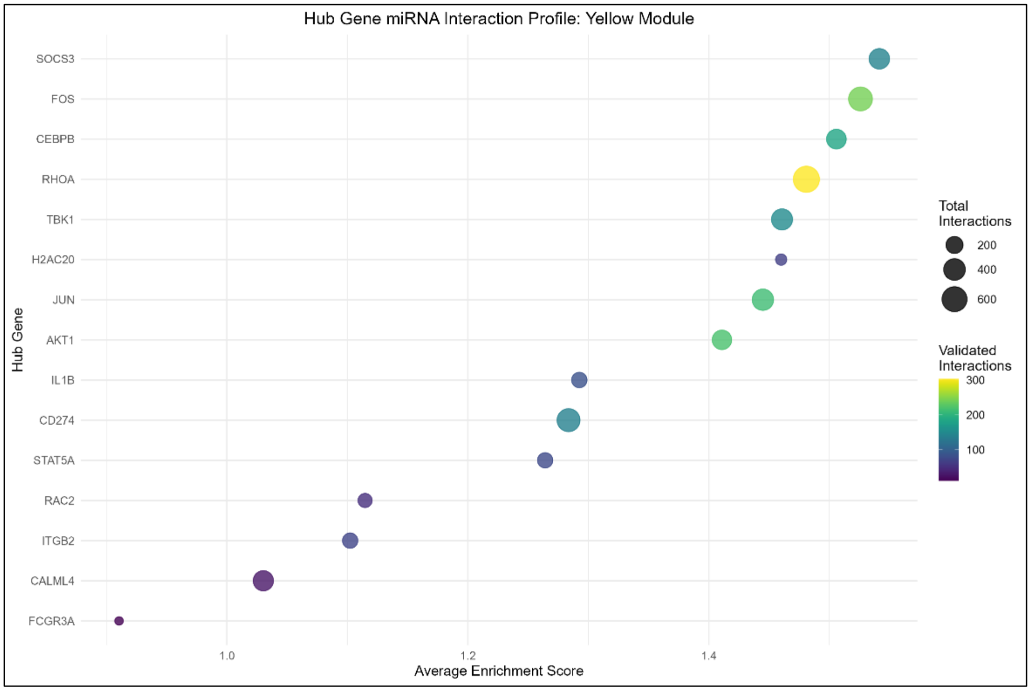

Figure S20. *Hub gene-miRNA interaction profile for the yellow module.*

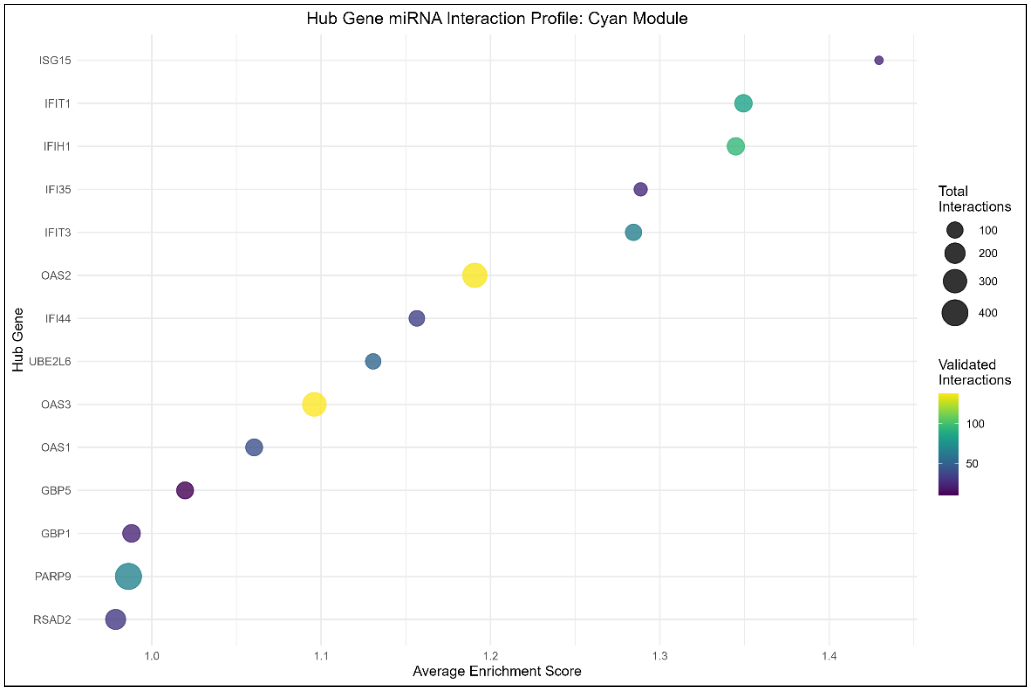

Figure S21. *Hub gene-miRNA interaction profile for the cyan module.*

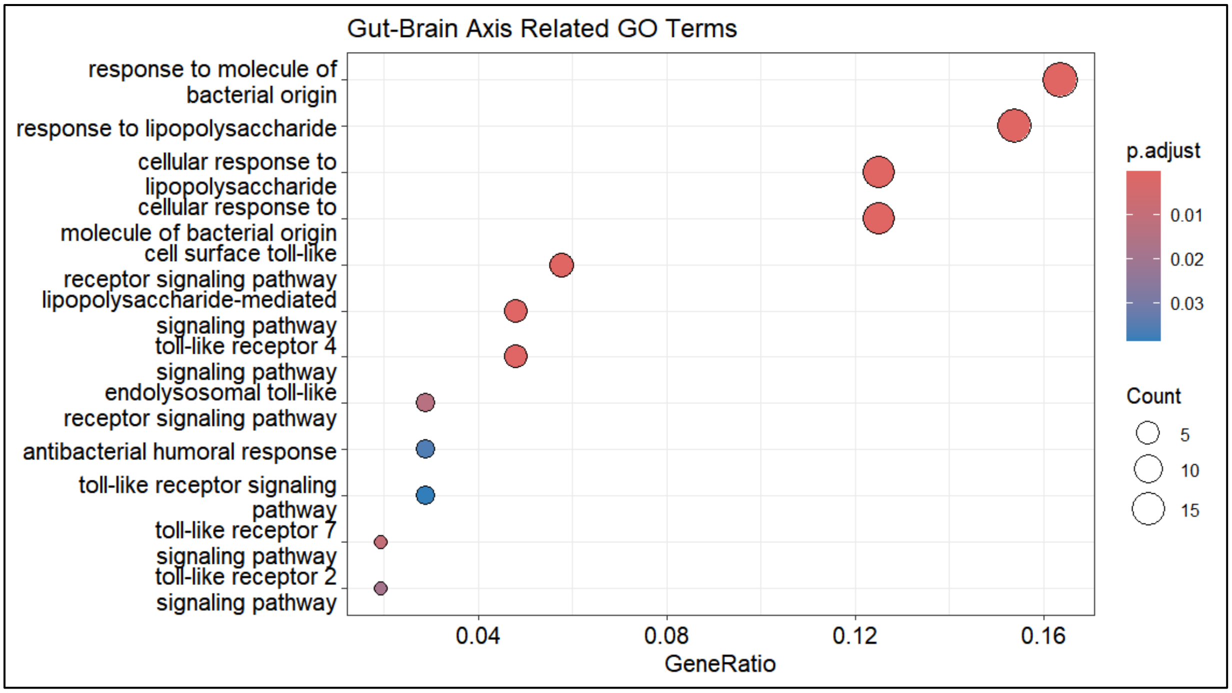

Figure S22. *Gene Ontology (GO) enrichment analysis of core regulatory hub genes for microbiota-gut-brain axis interactions. The dot plot illustrates significantly enriched Biological Processes related to systemic microbial and endotoxin responses. The x-axis*

represents the GeneRatio, dot size corresponds to the number of associated hub genes (Count), and dot color indicates the Benjamini-Hochberg adjusted p-value (p.adjust < 0.05).

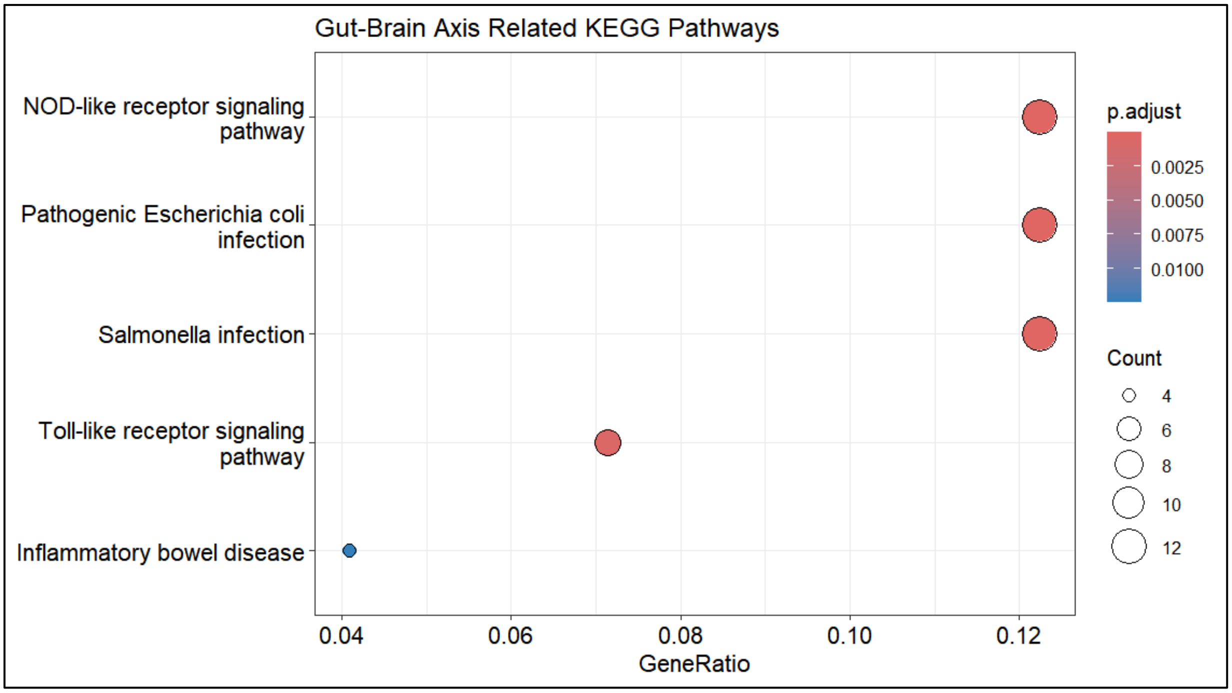

Figure S23. *KEGG pathway enrichment analysis of core regulatory hub genes highlighting host-microbiome interactions. The dot plot displays significantly enriched pathways associated with innate immune receptor signaling (NOD-like and Toll-like) and gut barrier permeability conditions. The x-axis represents the GeneRatio, dot size corresponds to the number of associated hub genes (Count), and dot color indicates the Benjamini-Hochberg adjusted p-value (p.adjust < 0.05)*

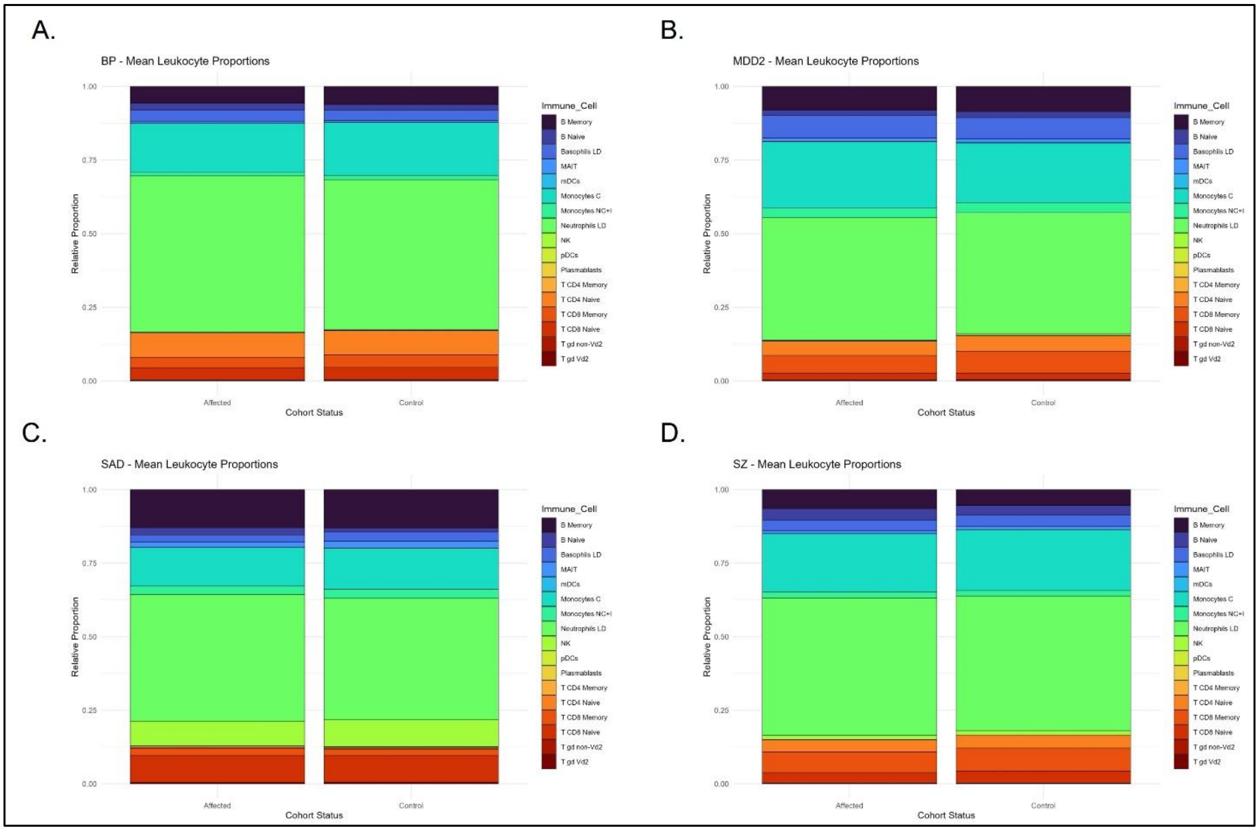

Figure S24. Mean Leukocyte Proportions Estimated via ABIS Deconvolution. *Stacked bar charts depicting the mean relative fractions of 17 distinct systemic immune cell populations within the whole-blood RNA-sequencing data across the BP, MDD, SAD, and SZ cohorts. Deconvolution was performed utilizing the CIBERSORT algorithm integrated with the ABIS (Agnostic Background Image Signature) matrix. The visualizations confirm stable and robust leukocyte architecture across all groups, validating that the sequence reads were successfully unmixed and that the captured transcriptomic profiles are predominantly driven by the leukocyte fraction.*

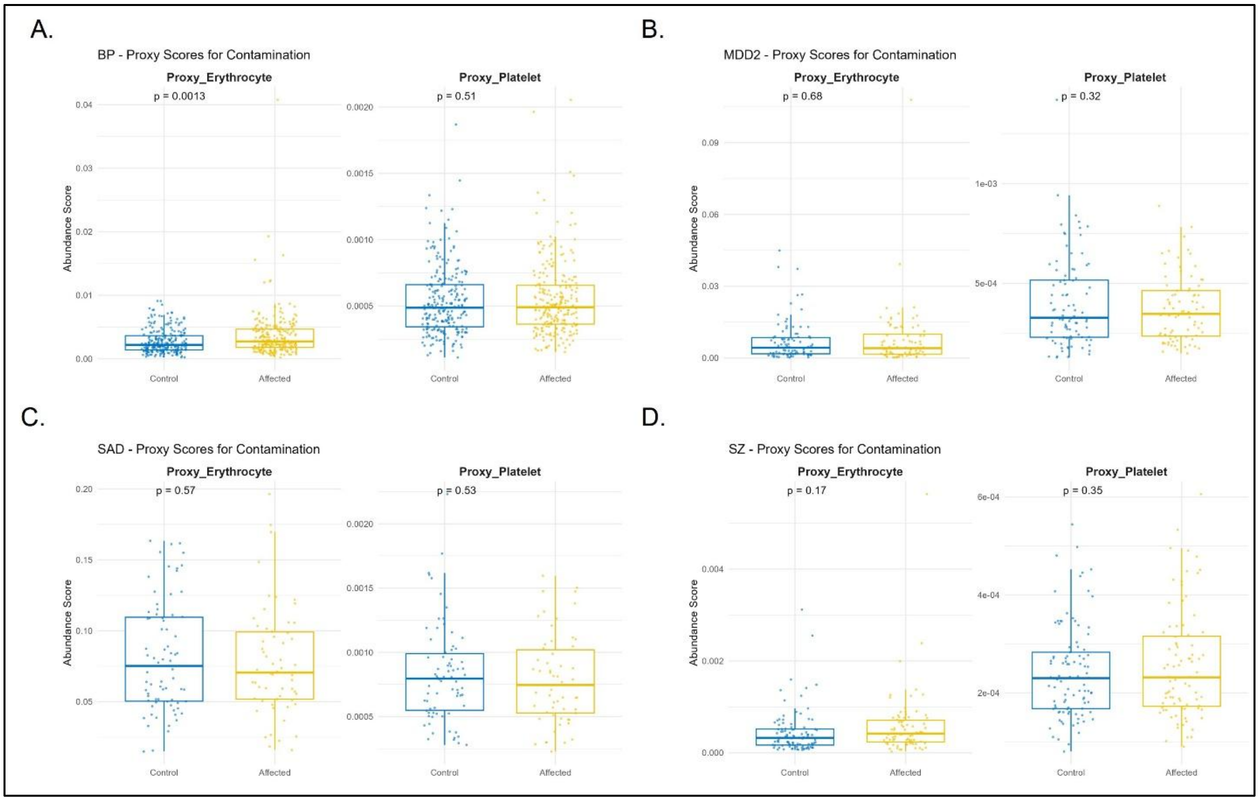

Figure S25. Empirical Proxy Scores for Non-Leukocyte Contamination Across Four Cohorts. *Boxplots illustrating the transcriptomic abundance of lineage-exclusive platelet and erythrocyte/reticulocyte marker genes, comparing Affected patients to healthy Controls across the BP, MDD, SAD, and SZ datasets. Proxy scores were calculated as the proportion of total library reads mapping to established MSigDB marker sets (e.g., PF4, PPBP for platelets; HBA1, HBB for erythrocytes). Statistical significance was determined using Student's t-tests. With the exception of the erythrocyte fraction in the BP cohort, no significant differences in non-leukocyte abundance were observed between cases and controls (all p > 0.05), indicating a uniform distribution of whole-blood background variance.*

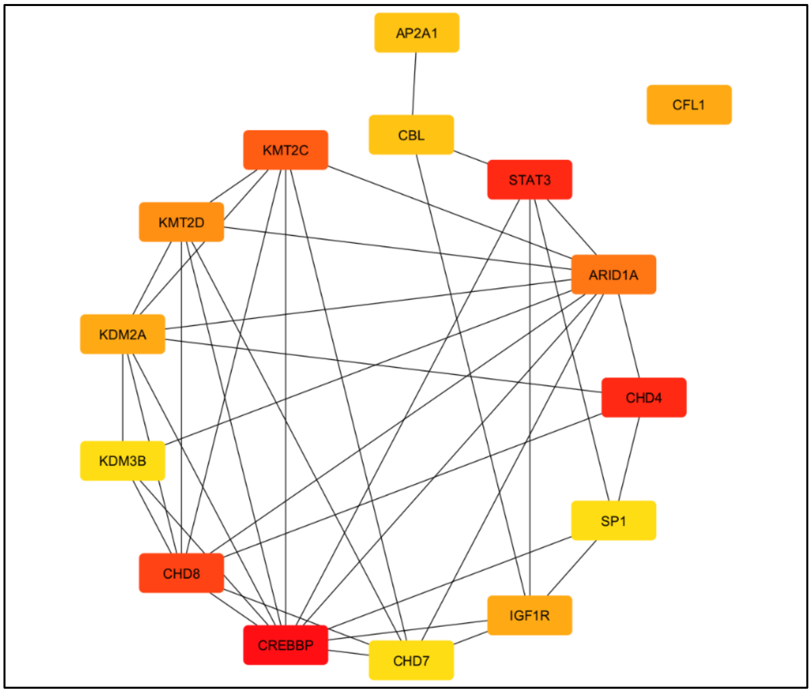

Figure S26. *Top 15 hub genes in Black module from the scrubbed dataset.*

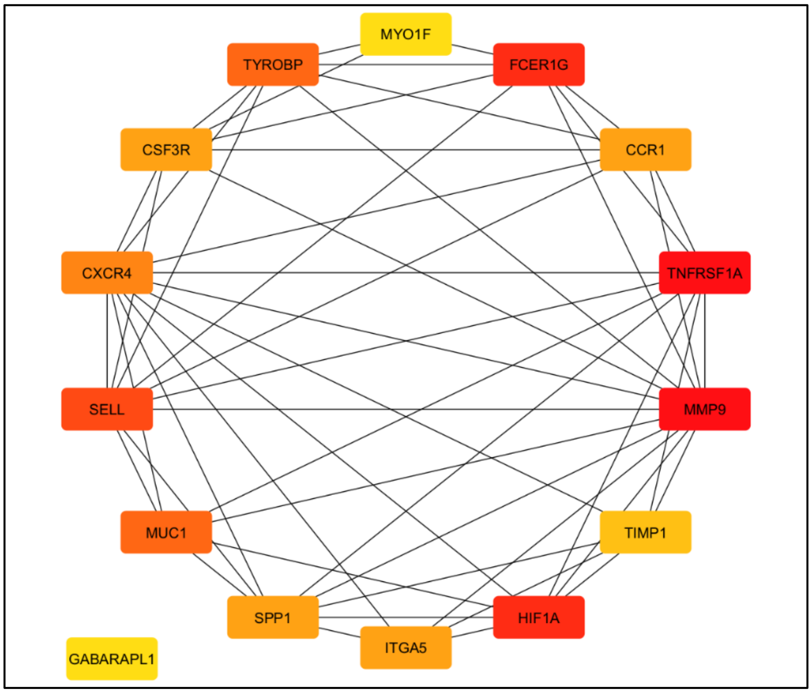

Figure S27. *Top 15 hub genes in Green module from the scrubbed dataset.*

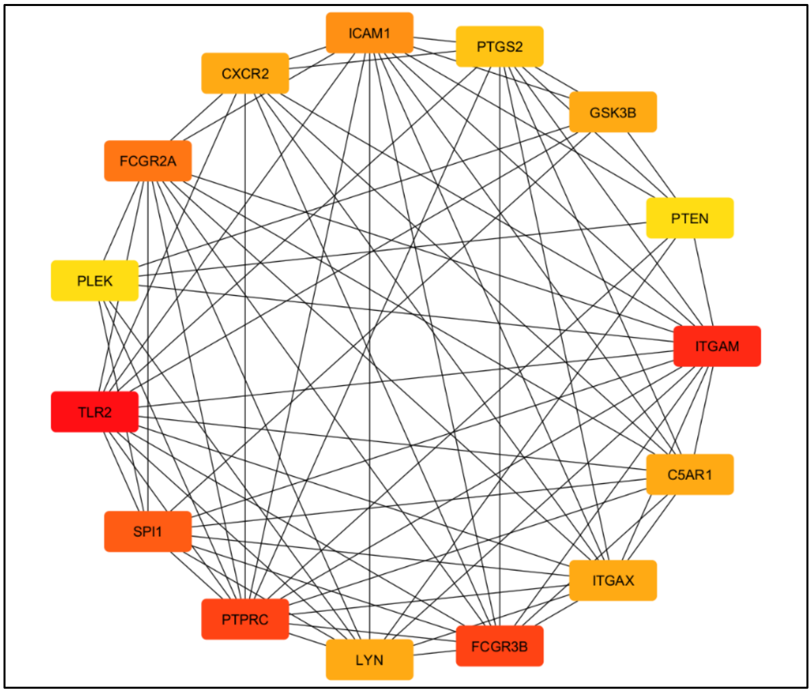

Figure S28. *Top 15 hub genes in Magenta module from the scrubbed dataset.*

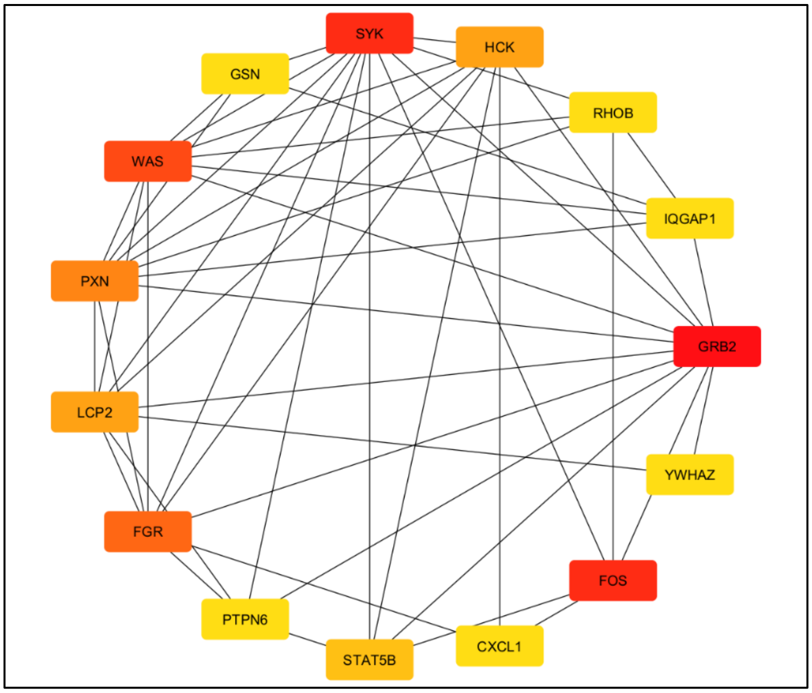

Figure S29. *Top 15 hub genes in Pink module from the scrubbed dataset.*

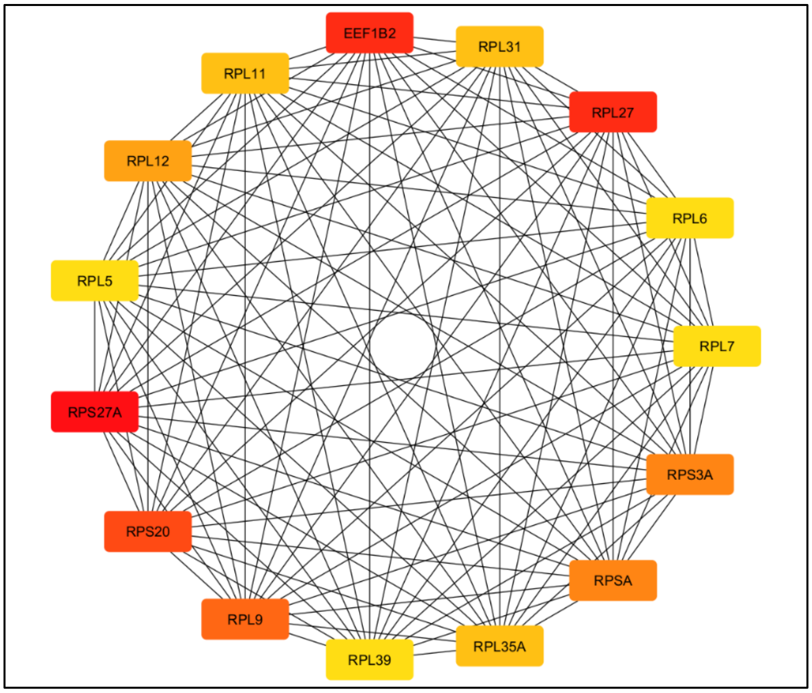

Figure S30. *Top 15 hub genes in Purple module from the scrubbed dataset.*

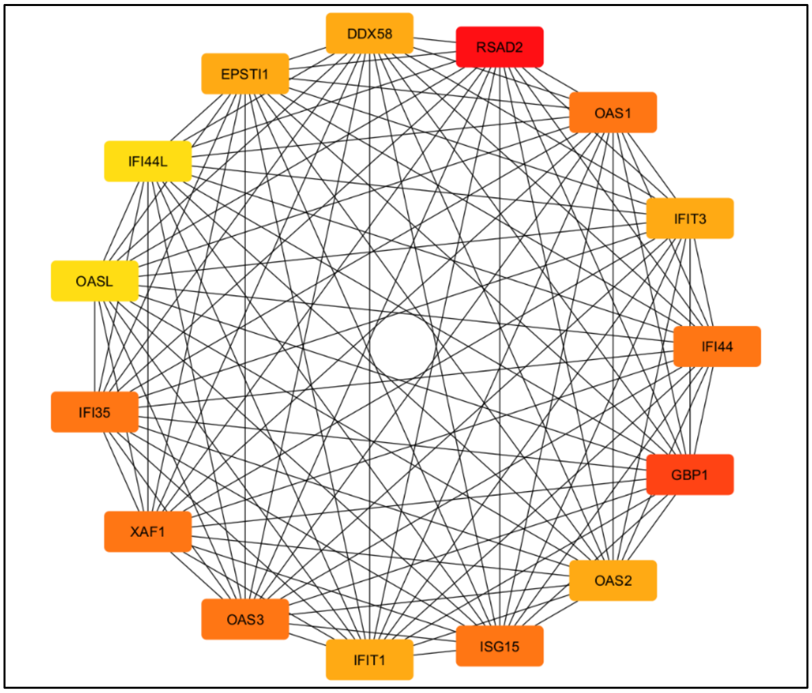

Figure S31. *Top 15 hub genes in Tan module from the scrubbed dataset.*

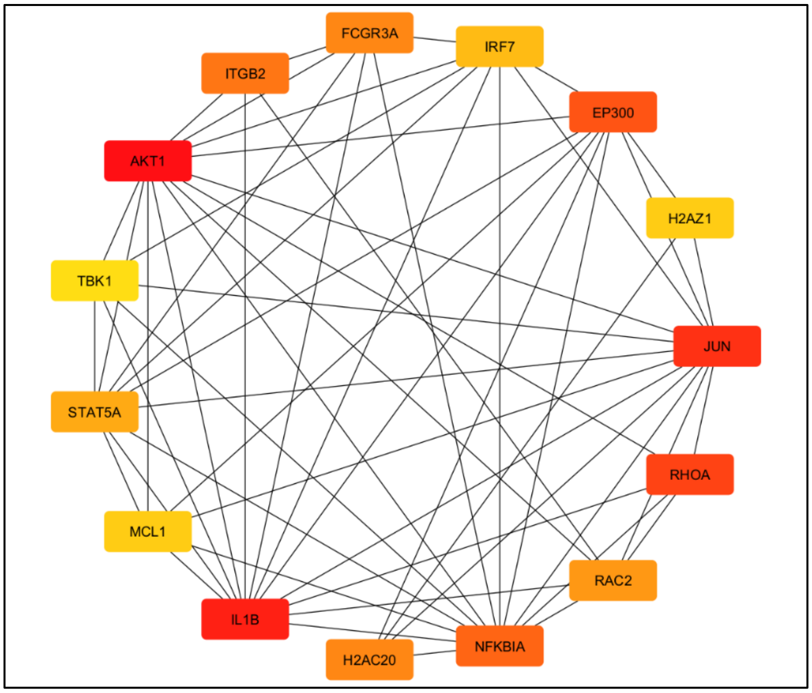

Figure S32. *Top 15 hub genes in Yellow module from the scrubbed dataset.*
