# Supplementary material for: Peripheral Transcriptomic Signatures Reveal Convergent Neuroinflammatory, Metabolic, and miRNA Dysregulation in Major Psychiatric Disorders
Source: Biology (Basel). 2026 Apr 24;15(9):673. doi: 10.3390/biology15090673 (PMC13162896; doi:10.3390/biology15090673)
Supplement: Supplementary file 1 [file biology-15-00673-s001.zip › Supplementary Files/Supplementary Tables.pdf]

Table S1. *Summary of Dataset Demographic and Disease Classification.*

Dataset	Total Samples	Disease group	Control Group	Age information	Sex information
MDD	169	MDD (80)	Normal (89)	Not Available	Female (105)<br>Male (64)
SZ	181	SZ (84)	Normal (97)	Not Available	Not Available
BP	480	BP I (226)<br>BP II (14)	Normal (240)	18 - 86	Female (265)<br>Male (213)
SAD	132	SAD (58)	Normal (74)	19 - 50	Females (91)<br>Males (41)

Table S2. *Cross-Disease Differential Expression of Candidate Genes in Psychiatric Disorders*

Gene	BP	SZ	MDD	SAD	Cross-Disease Consensus	Tier
**MMP9**			Up(p=0.054, LFC=0.543)		Nominally up in MDD	2
**TNFRSF1A**	Up(p=0.023, LFC=0.122)				Nominally up in BP	1
**FCER1G**	Up(p=0.008, LFC=0.180)		Up(p=0.154, LFC=0.1762)		BP, MDD	3
**TYROBP**	Up (p=0.012, LFC=0.150)				Nominally up in BP	1
**CXCR4**	Down (p=0.002, LFC=-0.128)	Down (p=0.120, LFC=-0.309)	Up (p=0.239, LFC=0.126)		BP, SZ, MDD	3
**CCR1**				Up (p=0.999, LFC=0.120)	Nominally up in SAD	2
**IL1RN**			Up (p=0.115, LFC=0.231)		Nominally up in MDD	2

**ITGA5**	Up (p=0.001, LFC=0.145)	Up (p=0.218, LFC=0.137)			BP, SZ	3
**ITGAM**			Up (p=0.398, LFC=0.124)		Nominally up in MDD	2
**SPI1**	Up (p=0.001, LFC=0.206)				Nominally up in BP	1
**ICAM1**			Up (p=0.034, LFC=0.338)		Nominally up in MDD	1
**C5AR1**	Up (p=9.148e-5, LFC=0.246)		Up (p=0.108, LFC=0.230)	Up (p=0.999, LFC=0.191)	BP, MDD, SAD	3
**FCGR2A**	Up (p=0.012, LFC=0.145)				Nominally up in BP	1
**ITGAX**		Up (p=0.254, LFC=0.164)			Nominally up in SZ	2
**CTSS**			Up (p=0.220, LFC=0.158)		Nominally up in MDD	2
**PTGS2**			Up (p=6.901e-5, LFC=0.912)		Nominally up in MDD	1
**EEF1B2**		Down (p=0.001, LFC=-0.315)	Up (p=0.022, LFC=0.242)		SZ, MDD	3
**RPL27**		Down (p=0.007, LFC=-0.287)			Nominally down in SZ	1
**RPS27A**		Down (p=0.0002, LFC=-0.287)	Up (p=0.007, LFC=0.2103)		SZ, MDD	3

**RPS20**		Down (p=0.0003, LFC=-0.413)	Nominally down in SZ	1
**RPS3**		Down (p=0.004, LFC=-0.311)	Nominally down in SZ	1
**RPL9**		Down (p=0.002, LFC=-0.387)	Nominally down in SZ	1
**RPSA**		Down (p=0.002, LFC=-0.304)	Nominally down in SZ	1
**FAU**		Down (p=0.018, LFC=-0.256)	Nominally down in SZ	1
**RPL12**		Down (p=0.115, LFC=-0.170)	Nominally down in SZ	2
**RPS3A**		Down (p=0.003, LFC=-0.343)	Nominally down in SZ	1
**RPL31**	Up (p=0.006, LFC=0.355)	Down (p=0.002, LFC=-0.340)	BP, SZ	3
**RPL39**	Up (p=0.014, LFC=0.326)	Down (p=0.005, LFC=-0.335)	BP, SZ	3
**RPL11**	Up (p=0.009, LFC=0.267)	Down (p=0.058, LFC=-0.214)	BP, SZ	3

**RPL35A**	Up (p=0.006, LFC=0.239)	Down (p=0.006, LFC=-0.307)			BP, SZ	3
**RPL5**		Down (p=0.002, LFC=-0.254)			Nominally down in SZ	1
**HCK**	Up (p=0.006, LFC=0.239)				Nominally up in BP	1
**LCP2**		Up (p=0.261, LFC=0.0973)			Nominally up in SZ	2
**NFKBIA**			Up (p=0.295, LFC=0.137)		Nominally up in MDD	2
**CXCL1**			Up (p=0.399, LFC=0.229)		Nominally up in MDD	2
**PAK1**	Up (p=0.01, LFC=0.120)				Nominally up in BP	1
**DDX3X**			Up (p=0.151, LFC=0.162)		Nominally up in MDD	2
**APBB1IP**				Up (p=0.999, LFC=0.094)	Nominally up in SAD	2
**ACTR2**				Up (p=0.999, LFC=0.0342)	Nominally up in SAD	2
**AKT1**				Down (p=0.999, LFC=-0.193)	Nominally up in SAD	2
**IL1B**	Up (p=0.005, LFC=0.180)				Nominally up in BP	1
**RHOA**	Up (p=0.021, LFC=0.086)	Down (p=0.221, LFC=-0.108)	Up (p=0.092, LFC=0.105)		BP, SZ, MDD	3
**ITGB2**			Up (p=0.354, LFC=0.119)		Nominally up in MDD	2

**CEBPB**	Up (p=0.016, LFC=0.131)			Up (p=0.999, LFC=0.130)	BP, SAD	3
**SOCS3**	Up (p=0.0089, LFC=0.221)				Nominally up in BP	1
**CD274**				Up (p=0.999, LFC=0.194)	Nominally up in SAD	2
**CALML4**				Up (p=0.999, LFC=0.0296)	Nominally up in SAD	2
**OAS1**	Down (p=0.004, LFC=-0.257)				Nominally down in BP	1
**RSAD2**				Up (p=0.515, LFC=0.909)	Nominally up in SAD	2
**OAS2**	Down (p=0.0002, LFC=-0.272)				Nominally down in BP	1
**IFI44**	Down (p=0.012, LFC=-0.294)				Nominally down in BP	1
**GBP5**	Down (p=0.024, LFC=-0.204)				Nominally down in BP	1
**ARID1A**				Down (p=0.999, LFC=-0.042)	Nominally down in SAD	2
**KMT2D**		Up (p=0.186, LFC=0.187)			Nominally up in SZ	2
**CFL1**			Up (p=0.343, LFC=0.087)	Down (p=0.999, LFC=-0.093)	MDD, SAD	3
**CBL**				Down (p=0.999, LFC=-0.074)	Nominally down in SAD	2

Table S3. *Biological Function and Psychiatric Relevance of Green Module Hub Genes and Direction.*

Gene	Biological Relevance	Relevance to Psychiatric Disorder

**MMP9**	Essential in extracellular matrix proteolysis and leukocyte migration. Particularly degrades type IV and V collagens.	Involved in demyelination, degradation of basement membrane leading to neuroinflammation, synaptic pruning and attenuated synaptic plasticity (MDD, BP) [57].
**TNFRSF1A**	Mediate TNF-α–induced signaling involved in inflammation, apoptosis, and cell survival, with both membrane-bound and soluble forms modulating inflammatory responses.	Proinflammatory imbalances, negatively correlated with cognitive performance and neurogenesis (MDD, SZ, BP, anxiety disorders) [96,163].
**FCER1G**	Adaptor protein involved in immunoreceptor transduction, allergic inflammation regulation, cytokine production and innate immune signaling	Immune response abnormalities (SZ) [94].
**TYROBP**	Mediate immune receptor signaling, inflammation, bone remodeling and brain myelination.	Brain homeostasis maintenance and affects microglia contributing to stress-related psychopathologies (SZ, BP) [60].
**CXCR4**	Chemokine receptor involved in cell migration, immune activation, neuronal survival, and MAPK and AKT pathways.	Implicated in abnormal development, proliferation and migration of neural progenitor cells for neurogenesis (MDD); Drives neuroinflammation (Anxiety Disorder, SZ) [50,164,165].
**CCR1**	Chemokine receptor that regulates immune cell recruitment and migration during inflammation. Promotes pro-inflammatory signaling including neuroinflammatory responses and cytokine release.	Implicated in inflammatory pathogenesis affecting neural processes [51].
**IL1RN**	Mediate excessive inflammation and immune dysregulation during innate immune responses.	Enhances IL-1B production yielding elevated inflammatory responses (SZ) [166].
**ITGA5**	Mediate cell adhesion, signaling and participates in inflammatory signaling	Stimulates spine development and synapse formation. Implicated in neurodegenerative diseases [167].

Table S4. *Biological Function and Psychiatric Relevance of Red Module Hub Genes and Direction.*

Gene	Biological Relevance	Relevance to Psychiatric Disorder
**ITGAM**	Mediate leukocyte adhesion, migration and phagocytosis of complement-opsonized particles. Regulates immune response such as neuroinflammation and neuronal apoptosis.	Linked with microglia activation for neuroinflammation regulation, neurogenesis and synaptogenesis (SZ) [97].
**SPI1**	Pioneer transcription factor that regulates hematopoietic cell fate.	Regulation of specialized microglial function. Highly expressed in MDD and SZ [62].
**ICAM1**	Cell adhesion molecule that mediates leukocyte adhesion and trans-endothelial migration	Interacts with integrin receptors in microglial cells affecting astrocyte-microglia interactions resulting in altered neuronal circuits (MDD) [168].
**C5AR1**	C5a receptor that binds to complement peptide for chemotaxis, granule release, calcium signaling and superoxide production in immune cells.	Modulates stress response via synaptic plasticity modulation and microglia/macrophage signaling leading to neuroinflammation (MDD) [169].
**FCGR2A**	Mediate phagocytosis and immune complexes clearance.	Dysregulation affects immunological responses, brain activity and neurodevelopmental processes (SZ). Linked with neuroinflammatory pathways [64].
**ITGAX**	Mediate neutrophil and monocyte adhesion and phagocytosis of complement-coated particles.	Alters microglial state to CD11c+ microglia responsible for neuronal apoptosis and myelinated rack repair [63].
**CTSS**	Cleaves invariant chain of MHC class II molecules for antigen presentation and acts as an elastase for extracellular matrix protein remodeling	Plays a role in microglial signaling and neuroinflammation [170].
**PTGS2**	Key enzyme in prostaglandin biosynthesis for inflammation and mitogenesis regulation.	polymorphism influences developing IFN-α induced depression and lowers DHA levels (MDD) [61].

Table S5. *Biological Function and Psychiatric Relevance of Pink Module Hub Genes and Direction*

Gene	Biological Relevance	Relevance to Psychiatric Disorder
**EEF1B2**	Translation elongation factor involved in the transfer of aminoacylated tRNAs to the ribosome.	Disrupted neurodevelopment implicated in intellectual disability, behavioral abnormalities and seizures [69].
**RPL27**	Large ribosomal subunit component required for proper rRNA processing and maturation.	Dysregulation is implicated in neurological and immunoinflammatory responses [65].
**RPS27A**	Ribosomal component	Activates NF-kB that promotes neuroinflammation and induce immune infiltration in brain tissues [66].
**RPS20**	Small ribosomal subunit component.	Prognostic marker for glioblastoma and medulloblastoma with roles in cerebrovascular development [171].
**RPS3**	Small ribosomal subunit component with endonuclease activity and DNA repair.	Potent neuroprotective effects for DNA and ischemic damage [68].
**RPL9**	Large ribosomal subunit component	Exhibits a proinflammatory response regulation [67].
**RPSA**	Key to 40s ribosomal subunit stability and late maturation. Acts as laminin receptors to mediate cell adhesion and signaling.	Modulates neuromorphogenesis, particularly dendrite orientation, extension, and formation. Also involved in neuronal migration [172].
**FAU**	40s ribosomal subunit component.	Regulates apoptosis, part of the immune response [173].
**RPL12**	Large ribosomal subunit component	Upregulation causes depressive symptoms [174].
**RPS3A**	Small ribosomal subunit component.	Activation of NF-kB and MAPK pathways, essential for proliferation and inflammatory response [175].
**RPL31**	Large ribosomal subunit component	Associated with metabolic and energy-related pathways. Implicated in SZ [70].
**RPL39**	RNA-binding component of the large ribosomal subunit	Dysregulation affects the proportion of inhibitory neurons [176].

**RPL11**	Ribosomal component	MDM2 regulator implicated in neuronal development and neuroinflammatory processes [177,178].
**RPL35A**	Large ribosomal subunit component with key roles in proliferation and viability of hematopoietic cells.	Upregulation causes depressive symptoms [174].
**RPL5**	Ribosomal component	Role in tumor suppression implicated in glioblastoma [179].

Table S6. *Biological Function and Psychiatric Relevance of Greenyellow Module Hub Genes and Direction.*

**Gene**	**Biological Relevance**	**Relevance to Psychiatric Disorder**
**HCK**	Tyrosine kinase that regulates innate immune responses, and mediate cytoskeletal remodeling and cell migration	Modulates NLRP3 inflammasome activity for macrophage and microglial inflammatory response. Associated with neurodegenerative diseases [71].
**LCP2**	Adapter protein that mediates T-cell receptor signaling and pro-inflammatory responses.	Affects microglia activation, linked to both neurodegenerative and neuroinflammatory diseases [74].
**NFKBIA**	Inhibition of NF-kB mediated inflammatory signaling.	Regulates inflammatory response with significant associations with depressive symptoms, BP, and SZ [53,180,181].
**CXCL1**	Chemokines for neutrophil recruitment and inflammatory response mediation.	Dysregulation affects inflammatory response and causes depressive behavior from neurotoxicity and or neuronal loss affecting neurogenesis and neurotransmitter functions (SZ, MDD) [51,182].
**PAK1**	P21 activated kinase that regulates cell morphology and motility. Influences synapse formation.	Involved in neurodevelopment, neuroplasticity and nervous system maturation. Implicated in depressive pathology (MDD) [183].
**DDX3X**	ATP-dependent RNA helicase involved in nuclear roles, and cytoplasmic translation signaling and viral replication.	Plays a role in neurogenesis and neuronal excitability and dysregulation leads to neurodevelopmental abnormalities and NPDs (SZ) [52].

**APBB1IP**	Linked with Ras activation to actin cytoskeletal remodeling.	Highly expressed in SZ, involved in immune response, neuronal development and cell motility [184].
**ACTR2**	ATP-binding subunit involved in cytoskeletal remodeling, cell motility and nuclear actin polymerization.	Involved in neurodevelopment notably, growth cone motility and axon guidance, dendritic spines and synapse development and memory decay [185].

Table S7. *Biological Function and Psychiatric Relevance of Black Module Hub Genes and Direction.*

**Gene**	**Biological Relevance**	**Relevance to Psychiatric Disorder**
**ARID1A**	Chromatin remodeler and facilitates neural stem cell self-renewal, neuronal differentiation and dendrite growth.	Regulator of neurogenic genes for neurogenesis. Dysregulation leads to anxiety like behavior [78].
**KMT2D**	Histone methyltransferase involved in beta-globin and estrogen receptor transcriptional regulation.	Irregularities affect synaptic development and activity dependent transcription. Heavily implicated in neuropsychiatric syndromes (SZ) and anxiety disorder [186,187].
**CFL1**	Actin-binding protein involved in cytoskeletal dynamics, cell morphology and epithelial organization. Supports neural tube morphogenesis and neural crest migration.	Dysregulation affects dendritic spine development, neural migration and long-term potentiation. Implicated in various neurodegenerative diseases, SZ and depression [188].
**CBL**	Negative regulator of various signaling pathways through ubiquitination of cell surface receptors.	Modulation of microglia-mediated neuroinflammation, and dendritic cell maturation. Implicated in neurodegenerative diseases [189].

Table S8. *Biological Function and Psychiatric Relevance of Yellow Module Hub Genes and Direction.*

**Gene**	**Biological Relevance**	**Relevance to Psychiatric Disorder**
**AKT1**	Regulates cell proliferation, survival metabolism and growth.	Critical for neural survival, neuronal excitability and synaptic plasticity. (MDD, SZ, BP, Anxiety) [84,85,100].

**IL1B**	Potent pro-inflammatory cytokine involved in cell proliferation, differentiation and apoptosis.	Affects inflammatory response neurogenesis, neuroplasticity and brain morphology. (MDD, SZ) [86,87].
**RHOA**	Promote actin cytoskeleton remodeling, cell shape regulation, attachment and motility.	Impaired dendritic spine density weakening synaptic plasticity affecting cognitive functions (SZ, Anxiety, MDD) [81].
**ITGB2**	Integrin component integral for cell adhesion and cell-surface mediated signaling.	Impaired cell signaling for anti-inflammatory regulators promoting neuroinflammation (SZ) [190].
**CEBPB**	Transcription factor that regulates genes involved in immune and inflammatory responses.	Highly expressed in SZ and BP resulting in neuroinflammation, microglia activation, pyroptosis and stress-induced depressive behaviors [191].
**SOCS3**	Regulates cytokine signal transduction.	Regulates immunological and inflammatory processes. Affects STAT3 signaling leading to neuroinflammation (SZ) [192].
**CD274**	Integral part of induction and maintenance of immune tolerance to self.	Implicated in immune dysregulation [193].
**CALML4**	Mediates epithelial brush border differentiation.	Modulates Ca2+/calmodulin-dependent protein kinase II pathway and dysregulation results in attenuated learning, memory, synaptic plasticity and LTP [194].

Table S9. *Biological Function and Psychiatric Relevance of Cyan Module Hub Genes and Direction*

**Gene**	**Biological Relevance**	**Relevance to Psychiatric Disorder**
**OAS1**	Key protein in innate cellular antiviral response and implicated in cell growth and apoptosis.	Mediate proinflammatory responses under elevated interferon levels. Contributes to synaptic damage, phagocytosis and neuronal and oligodendrocyte death [90].
**RSAD2**	Mediate cellular antiviral response and innate immune signaling.	Role in chemotaxis and inflammatory processes. Correlated with depressive symptoms [92].

**OAS2**	Similar antiviral role as OAS1	Responds under IFNs and mediate inflammatory cytokines and chemokines inducing inflammatory responses and linked with neurocognitive decline [138].
**IFI44**	Involved in immune response to virus and bacterium.	Affects IFNB expression leading to neuroinflammation [195].
**GBP5**	Activator of NLRP3 inflammasome assembly and part of innate immunity for bacterial, viral and protozoan pathogens.	Role in transcriptional regulation, inflammation and innate immunity with correlation to anhedonia, a symptom of MDD [196].
